# The Genomic and Transcriptomic Landscape of a HeLa Cell Line

**DOI:** 10.1534/g3.113.005777

**Published:** 2013-03-11

**Authors:** Jonathan J. M. Landry, Paul Theodor Pyl, Tobias Rausch, Thomas Zichner, Manu M. Tekkedil, Adrian M. Stütz, Anna Jauch, Raeka S. Aiyar, Gregoire Pau, Nicolas Delhomme, Julien Gagneur, Jan O. Korbel, Wolfgang Huber, Lars M. Steinmetz

**Affiliations:** * European Molecular Biology Laboratory, Genome Biology Unit, 69117 Heidelberg, Germany; † University Hospital Heidelberg, Institute of Human Genetics, 69120 Heidelberg, Germany

**Keywords:** genomics, transcriptomics, HeLa cell line, resource, variation

## Abstract

HeLa is the most widely used model cell line for studying human cellular and molecular biology. To date, no genomic reference for this cell line has been released, and experiments have relied on the human reference genome. Effective design and interpretation of molecular genetic studies performed using HeLa cells require accurate genomic information. Here we present a detailed genomic and transcriptomic characterization of a HeLa cell line. We performed DNA and RNA sequencing of a HeLa Kyoto cell line and analyzed its mutational portfolio and gene expression profile. Segmentation of the genome according to copy number revealed a remarkably high level of aneuploidy and numerous large structural variants at unprecedented resolution. Some of the extensive genomic rearrangements are indicative of catastrophic chromosome shattering, known as chromothripsis. Our analysis of the HeLa gene expression profile revealed that several pathways, including cell cycle and DNA repair, exhibit significantly different expression patterns from those in normal human tissues. Our results provide the first detailed account of genomic variants in the HeLa genome, yielding insight into their impact on gene expression and cellular function as well as their origins. This study underscores the importance of accounting for the strikingly aberrant characteristics of HeLa cells when designing and interpreting experiments, and has implications for the use of HeLa as a model of human biology.

HeLa was the first human cell line established in culture (Gey *et al.* 1952) and has since become the most widely used human cell line in biological research. Its application as a model organism has contributed to the characterization of important biological processes and more than 70,000 publications. The cell line originates from a cervical cancer tumor of a patient named Henrietta Lacks, who later died of her cancer in 1951 (Skloot 2010). One of the earliest uses of HeLa cells was to develop the vaccine against the polio virus (Scherer *et al.* 1953). Recently, two Nobel prizes have been awarded for discoveries where HeLa cells played a central role, namely the link between human papilloma virus and cervical cancer (2008, Harald zur Hausen) and the role of telomerase in preventing chromosome degradation (2011, Elizabeth Blackburn, Carol Greider, and Jack Szostak).

During the last 10 years, HeLa has been used to pioneer omics approaches such as microarray-based gene expression profiling (Chaudhry *et al.* 2002; Whitfield *et al.* 2002; Hnilicová *et al.* 2011) and to investigate responses to environmental (Murray *et al.* 2004; Ludwig *et al.* 2005) and genetic perturbations (Jaluria *et al.* 2007). RNA interference screens in HeLa have led to the discovery and functional classification of genes involved in mitosis/cytokinesis (Chaudhry *et al.* 2002; Kittler *et al.* 2004; Zhu *et al.* 2005; Kim *et al.* 2007; Neumann *et al.* 2010; Hnilicová *et al.* 2011), endocytosis (Pelkmans *et al.* 2005), and other cellular processes (Alekseev *et al.* 2009; Fuchs *et al.* 2010). The transcriptome of HeLa has been characterized with second-generation sequencing technologies, *e.g.*, poly(A)-RNA (Wu *et al.* 2008) and small RNAs (Affymetrix ENCODE Transcriptome Project & Cold Spring Harbor Laboratory ENCODE Transcriptome Project 2009), and HeLa has been used as a model system for a combined deep proteome and transcriptome analysis (Nagaraj *et al.* 2011).

Although such studies have led to breakthroughs in molecular biology, they were designed and analyzed without genomic sequence information for the HeLa cell line. Instead, researchers have used the human reference genome, despite its evident differences from that of a cancer cell line that has been evolving in the laboratory for several decades. Indeed, substantial chromosomal aberrations in the HeLa cell line have been revealed by cytogenetic methods (Chen 1988; Francke *et al.* 1973; Kraemer *et al.* 1974; Heneen 1976; Nelson-Rees *et al.* 1980; Stanbridge *et al.* 1981; Mincheva *et al.* 1987; Popescu & Dipaolo 1989; Ruess *et al.* 1993; Macville *et al.* 1999). A combination of these techniques [comparative genomic hybridization (CGH), fluorescence *in situ* hybridization (FISH), and spectral karyotyping (SKY)] has been used to determine the karyotype of a CCL2 HeLa cell line (Macville *et al.* 1999). This cell line contained two subclonal populations, which were both hypertriploid (3n+), with a variable total number of chromosomes (76−80) and a variable number of abnormal chromosomes (22−25) per cell. The comparison of their spectral karyotype with previously published G-banding karyotypes (Francke *et al.* 1973; Kraemer *et al.* 1974; Heneen 1976; Nelson-Rees *et al.* 1980; Stanbridge *et al.* 1981; Mincheva *et al.* 1987; Chen 1988; Popescu & Dipaolo 1989) and FISH (Ruess *et al.* 1993) indicated high concordance between independent measurements of chromosomal aberrations in HeLa. These well-documented genomic aberrations underscore the need for a HeLa reference genome.

In this study, we created a genomic and transcriptomic resource for a HeLa cell line based on deep DNA and RNA sequencing. We determined single-nucleotide variants (SNVs), structural variants (SVs), and copy number (CN) along the genome. We profiled the HeLa transcriptome and assessed differences in expression between our HeLa cell line and normal human tissues by comparing to publicly available RNA-Seq data from the Illumina Human BodyMap 2.0. Our data can inform the design of future experiments and allow for the reinterpretation of previously generated data. The specific cell line analyzed here [HeLa Kyoto H2B-mRFP and mEGFP-α-tubulin (Steigemann *et al.* 2009)] has previously been used in genome-wide RNA interference (RNAi) studies (Fuchs *et al.* 2010; Neumann *et al.* 2010) and is commercially available.

## Materials and Methods

The data and resources generated in this study, including the genome sequence (FASTA format), DNA and RNA sequence reads (FASTQ), structural variants (VCF), single nucleotide variants (VCF), copy number (tab-delimited text), SIFT predictions (tab-delimited text), a tool to perform genome coordinate translation, and the analysis scripts have been deposited with the database of Genotypes and Phenotypes (dbGaP, http://www.ncbi.nlm.nih.gov/gap) under accession no. phs000643.v1.p1.

### Cell line, culture conditions, and DNA/RNA preparation

The cell line, HeLa H2B-mRFP and mEGFP-α-tubulin, was derived from the HeLa Kyoto background (Steigemann *et al.* 2009; Neumann *et al.* 2010). The cells can be purchased from CLS Cell Lines Service GmbH (catalog number 300670). Cells were cultured for seven passages in Dulbecco’s modified Eagle medium (Gibco) containing 4.5 g/L glucose (Sigma-Aldrich), 10% heat-inactivated fetal bovine serum (Sigma-Aldrich), 2 mM glutamine (Sigma-Aldrich), 100 U/mL penicillin, and 100 μg/mL streptomycin (Sigma-Aldrich) and incubated at 37° and 5% CO_2_. Cells were harvested at 80% confluency. RNA-free genomic DNA was prepared using the QIAGEN DNeasy Blood and Tissue kit (QIAGEN). Total RNA was extracted using Trizol.

The cell line was confirmed to be free of mycoplasma contamination using the MycoAlert mycoplasma detection kit (Lonza). Short tandem repeat (STR) genotyping was performed to verify the identity of this cell line by comparing it to nine published marker profiles for HeLa. Polymerase chain reaction (PCR) was performed with the AmpFLSTR Identifiler kit from ABI/Life Technologies. This system assayed 16 different STR markers, including the nine markers ATCC and DSMZ published as standard reference profiles for their cell lines.

### Library preparations

#### DNA-Seq library preparation:

RNA-free genomic DNA was prepared using the QIAGEN DNeasy Blood and Tissue kit (QIAGEN). For short insert size paired-end libraries (PE), the genomic DNA was sheared using Covaris S2. The sequencing library was prepared following the manufacturer’s protocol (Illumina) (Bentley *et al.* 2008) using NEBNext DNA Sample Prep Master Mix Set 1 (NEB).

For long insert size mate pair libraries (MP), 10 μg of genomic DNA were sheared using the Hydroshear (GeneMachines), and libraries were prepared using the Illumina MP v2 reagents and protocol.

#### Strand-specific RNA-Seq library preparation:

Poly-A RNA isolation, RNA fragmentation, and complementary DNA (cDNA) synthesis protocols were performed as in Yoon and Brem (2010). The cDNA was processed for DNA library preparation according to Parkhomchuk *et al.* (2009). In summary, the protocol includes RNA fragmentation, first-strand synthesis, and second-strand synthesis using deoxyribonucleotide triphosphates (dNTPs) and deoxyuridine triphosphates (dUTPs). End repair, A-tailing, and ligation were then performed as well as size selection for fragments of 300−350 bp. The second strand was cleaved by hydrolysis of uracil in the dsDNA. The resulting strand-specific cDNA was amplified before sequencing. Three biological replicates were prepared.

### Sequencing and alignment

#### DNA-Seq:

The PE library was sequenced on eight lanes of HiSeq 2000 (Illumina) using the manufacturer’s recommended pipeline (v1.1). The resulting 101 nt paired-end reads (1.1 billion) were mapped with GSNAP (Wu and Nacu 2010) to the human reference genome (GRCh37). Only unique alignments for each read were reported (-n 1 -Q); otherwise, default parameters were used. A total of 86% of the total read number (946 million) was aligned.

The MP library was sequenced on one lane of HiSeq 2000 (Illumina) as described previously. A total of 81% of the total read number (383 million) was aligned, resulting in a 155× physical coverage (number of overlapping fragments) after redundancy filtering.

#### RNA-Seq:

The three libraries were sequenced on nine lanes of the Genome Analyzer II (Illumina) using the manufacturer’s recommended pipeline (v1.18). Two paired-end read lengths were generated: 76 nt and 105 nt. RNA sequencing reads (450 million) were mapped to our HeLa genome using the same method as for the DNA-Seq reads. 56% of the total read number (253 million) was aligned. Reads from the BodyMap 2.0, provided by Illumina (ENA number: ERP000546) were downloaded and aligned to the human reference genome (GRCh37) with GSNAP using the same parameters as described above.

### DNA-Seq analysis

From the fully preprocessed alignment (BAM file), we computed the depth of coverage of the HeLa genome in 10-kb bins. We applied a mappability correction by dividing the count for each bin by the proportion of mappable positions in that bin. If this proportion was <0.5, we discarded the counts for the bin and assigned it the value NA. A position in the genome was called mappable if a simulated read of length 101 nt (the length used in our DNAseq experiment) starting at that position had exactly one valid alignment when processed with our alignment pipeline as described above.

We also adjusted the coverage according to the GC-dependent bias. We used a local fit to describe the relation of GC-count to coverage per bin and the ratio between that fit and the desired coverage of 60 bp as the adjustment factor. Supporting Information, Figure S4 shows the effect of this adjustment.

To describe the extent of CN aberrations in HeLa, we created a track segmenting the genome according to integer CN, which was obtained with the R/Bioconductor package DNACopy (Venkatraman and Olshen 2007) followed by mixture model fitting.

To transform the data to a scale compatible with the DNACopy software, we applied the function $\log_{2}\frac{x}{x_{0}}$ to the GC-adjusted depth-of-coverage data *x*, where *x*_0_ is the median GC-adjusted coverage of a manually curated region of CN 2 on chromosome 4 (see Figure S4C).

#### CN segmentation with DNAcopy:

We used the R/Bioconductor package DNACopy to generate a segmentation of the 10-kb binned log2-ratio values (parameters: undo.splits=“sdundo” and undo.SD=2). From the segment averages s, we calculated copy number estimates 2^s+1^, to which we fitted a mixture model of m=8 normal distributions with means fixed to 1, \x{2026}, m and weights and standard deviations estimated from the data. Segments were then assigned to the mixture components by a Bayes classifier, using the mixture component weights as prior probabilities, and requiring posterior probability ≥0.95.

#### SNVs and small indels:

To call SNVs, we used the pipeline described in the section “Best practice Variant Detection with the GATK v4, for release 2.0” of the GATK webpage at http://www.broadinstitute.org/gatk/guide/. This pipeline consists of duplicate removal, indel realignment, and base quality score recalibration. We used a minimum confidence score threshold of 30 as a filtering parameter for the GATK UnifiedGenotyper tool (McKenna *et al.* 2010).

Short indels (1−50 bp) were called using the program PINDEL (Ye *et al.* 2009) with default parameters and an insert size of 302 nt, estimated from a sample of one million read pairs.

The zygosity track was based on the distribution of the allele frequencies of all SNVs called by GATK. To identify homozygous regions in the HeLa genome, we calculated the proportion of homozygous SNV calls in 100-kb bins and called a bin homozygous if the proportion of homozygous calls was >0.5. To identify large blocks of homozygous regions, we applied a segmentation algorithm to the binned proportions of homozygous calls (Bioconductor package DNAcopy) and then classified the segments based on the same criterion as above (homozygous proportion >0.5) to obtain a track that segments the genome into homozygous and heterozygous blocks. This approach was applied in the same way for our SNV calls as well as for the SNV calls obtained by the HapMap consortium (The International Hapmap Consortium 2003) on three individuals: NA12878, NA12891, and NA12892.

The mutational spectrum of a set of SNVs was determined by classifying all SNVs contained in the set by their type of mutation (C > A, C > G, C > T, T > A, T > C, T > G) and the sequence context (*i.e.*, the preceding and the following base). The resulting count matrix with dimensions 4 × 4 × 6 (with each cell representing a mutation of one base triplet into another) was then normalized for the observed frequency of each source base triplet in the genome that the calls were made against. An additional conversion into percentage was performed to allow for comparison of SNV sets with different sizes.

The stacked barplots were generated on the count matrix after normalization for source-triplet frequency by discarding the context information and summing all counts by their associated mutation type (*e.g.*, C > A).

We computed the mutational spectra of all called SNVs stratified by interesting subgroups [contained in the Single Nucleotide Polymorphism database (dbSNP), reported in the 1000 Genomes Project, homozygous, heterozygous, and HeLa-specific]. We plotted the distribution of mutations within those groups with and without sequence context (Figure S1). We observed a different behavior (smaller proportion of T > C) among the HeLa-specific SNV calls compared with those overlapping with known sites (dbSNP; 1000 Genomes Project).

To investigate the reason for this differing pattern, we stratified the HeLa-specific SNV calls by the local coverage to find effects caused by differences in available data. Figure S2 shows a heatmap and barplot of these stratified calls, showing that the mutation pattern in the context changes based on the local coverage and therefore the distribution of mutations also differs. We chose SNVs with a local coverage between 10 and 60 because they were the most similar to the expected distribution, which is represented by the column labeled dbSNP in the barplot.

#### Large SVs:

Structural rearrangements were detected using paired-end mapping (Korbel *et al.* 2007; Rausch *et al.* 2012a). The mate pair structural rearrangement calls were filtered using phase I 1000 Genomes Project (http://1000genomes.org) genome data as well as germline data of additional whole-genome sequencing samples (Jones *et al.* 2012) to distinguish cell-line-specific from common SVs as well as rearrangement calls caused by mapping artifacts. We only considered for further analysis those rearrangements that were present in at most 0.5% of the 1000 Genomes Project samples assessed and not in the additional germline samples. Two rearrangement calls were considered to be equivalent, hence constituting a likely common variant, if they displayed an overlap in terms of genomic coordinates. The paired-end structural rearrangement calls were required to have paired-end (>=2) and split-read support, with the split-read consensus sequence aligning to the reference at >=90% identity and overlapping sites (>=10% reciprocal overlap) removed. Deletions overlapping SINEs and LINEs were filtered out; for inversions, paired-end read support at both sides was required. Deletions were called homozygous if the median coverage in their interval was less than 1.

93 deletions, 52 tandem duplications, and 12 translocations were randomly selected and processed to validate by PCR, in addition to 3 manually selected inversions. PCR primers were designed for predicted SVs with Primer3 (parameters: T_m_ = 60°; T_min_ = 57°; T_max_ = 63°; optimal length = 25 bp; minlength = 18 bp; max-length = 26 bp; mingc = 40; maxgc = 60). Primers matching to repeat databases (Jurka *et al.* 2005) were excluded. The primers designed for deletion, inversion, and tandem duplication events follow the rules described in Figure S5. The primer pairs designed to validate translocations spanned the breakpoint-junction-sequences of predicted SVs. A total of 10 ng each of HeLa DNA were amplified in 30-µL PCRs using 0.3 µL of Phire Polymerase (F-122S; Thermo Scientific), 5X Phire reaction buffer, 200 µM dNTPs, and 0.5 µM of primers for 36 cycles. The PCR cycle included initial denaturation at 98° for 30 sec, denaturation at 98° for 10 sec, annealing at 60° for 10 sec, extension at 72° for 2 min, and final extension for 5 min at 72°. The products were run on 0.8–1% agarose to determine their sizes.

#### Multiplex FISH (M-FISH):

M-FISH was performed as described by Geigl *et al.* (2006). In brief, seven pools of flow-sorted whole chromosome painting probes were amplified and directly labeled using seven different fluorochromes (DEAC, FITC, Cy3, Cy3.5, Cy5, Cy5.5, and Cy7) using degenerative oligonucleotide-primed PCR (DOP-PCR). Metaphase chromosomes immobilized on glass slides were denatured in 70% formamide/2× saline sodium citrate (SSC), pH 7.0, at 72° for 2 min followed by dehydration in a degraded ethanol series. Hybridization mixture containing combinatorially labeled painting probes, an excess of unlabeled cot1 DNA, 50% formamide, 2× SSC, and 15% dextran sulfate was denatured for 7 min at 75°, preannealed at 37° for 20 min, and hybridized at 37° to the denaturated metaphase preparations. After 48 hr, the slides were washed in 2× SSC at room temperature for 3× 5 min followed by two washes in 0.2× SSC/0.2% Tween-20 at 56° for 7 min each. Metaphase spreads were counterstained with 4.6-diamidino-2-phenylindole (DAPI) and covered with antifade solution. Metaphase spreads were captured using a DM RXA epifluorescence microscope (Leica Microsystems, Bensheim, Germany) equipped with a Sensys CCD camera (Photometrics, Tucson, AZ). The camera and microscope were controlled by the Leica Q-FISH software and images were processed using the Leica MCK software and presented as multicolor karyograms (Leica Microsystems Imaging solutions, Cambridge, United Kingdom).

#### Virus integration detection:

We aligned the DNA-Seq reads obtained from HeLa to a genome consisting of the human reference (GRCh37) and a set of known whole virus genomes obtained from the viral genome resource (National Center for Biotechnology Information) (Bao *et al.* 2004). Potential virus insertions were found in read pairs with one read mapping to a chromosome from the human reference and one read mapping to a virus genome. We extracted all such read pairs and performed a clustering based on overlap to find clusters of read pairs indicating virus insertions. To account for similarities between virus genomes, we also clustered read-pairs together where the reads on the human genome overlapped and the reads on the virus genomes mapped to viruses from the same family (papillomavirus, herpesvirus, adeno-associated virus, adenovirus, lentivirus, poxvirus and retrovirus).

### RNA-Seq analysis

#### Expression level and CN:

The number of reads per gene were counted using HTSeq (Anders; http://www-huber.embl.de/users/anders/HTSeq/). The *log*_10_ of the count per gene divided by the length of the gene in kilobases was considered a proxy of expression level.

We called SNVs in the RNA sequencing data by using the same pipeline used for the DNA sequencing data following the best practice variant detection with GATK v4 (DePristo). Allelic SNV counts in genomic regions of CN 3 were extracted from the output of the GATK caller.

#### Comparing HeLa with the Illumina BodyMap 2.0:

The BodyMap 2.0 data (ENA number: ERP000546) were previously generated on a HiSeq 2000 (Illumina) from 16 human tissue types, including adrenal, adipose, brain, breast, colon, heart, kidney, liver, lung, lymph, ovary, prostate, skeletal muscle, testes, thyroid, and white blood cells. The 16-tissue reads were downloaded and aligned by GSNAP with the parameters used for the HeLa transcriptome sequencing reads. To explore which functions are specific to HeLa compared with other human tissues, the number of reads per gene were counted as described in the previous section for both samples, and compared using the DESeq package (Anders and Huber 2010). We used DESeq for normalization and preprocessing (size factor correction, variance stabilization) and estimated the physiological range of counts for each gene from the 16 BodyMap samples. We obtained z-scores (*z*) for our HeLa samples compared to the distribution of the 16 BodyMap samples. With the estimated standard deviation $\bar{\sigma }$ and mean $\bar{\mu }$ of the 16 BodyMap samples and *x* being the vector of means of the three normalized HeLa RNA-Seq counts, the z-scores were computed as follows:

$$\text{z}=\frac{x-\bar{\mu }}{\bar{\sigma }}$$

We used a cutoff of 3 to determine which genes were significantly overexpressed compared to the physiological range. We defined non-expressed genes as those with a mean of less than 1 count per kilobase.

We searched for enriched terms using model-based gene set analysis (MGSA) (Bauer *et al.* 2011) with 10 independent runs of the Markov chain of 10^9^ steps each. For each parameter, we used a regularly spaced grid with 11 points. Default search intervals for the model parameters proved inappropriate because the maximum of the posterior was often reached at the bounds, implying that the most likely fits were outside the search intervals. Thus, the search intervals for the parameters p, alpha, and beta were set to [0.001, 0.01], [0.001, 0.05], and [0.7, 0.9] respectively for the highly expressed genes, and [0.0001, 0.02], [0.001, 0.2], and [0.7, 0.95] for the non-expressed genes.

## Results

### Genomic landscape

**Figure 1:**
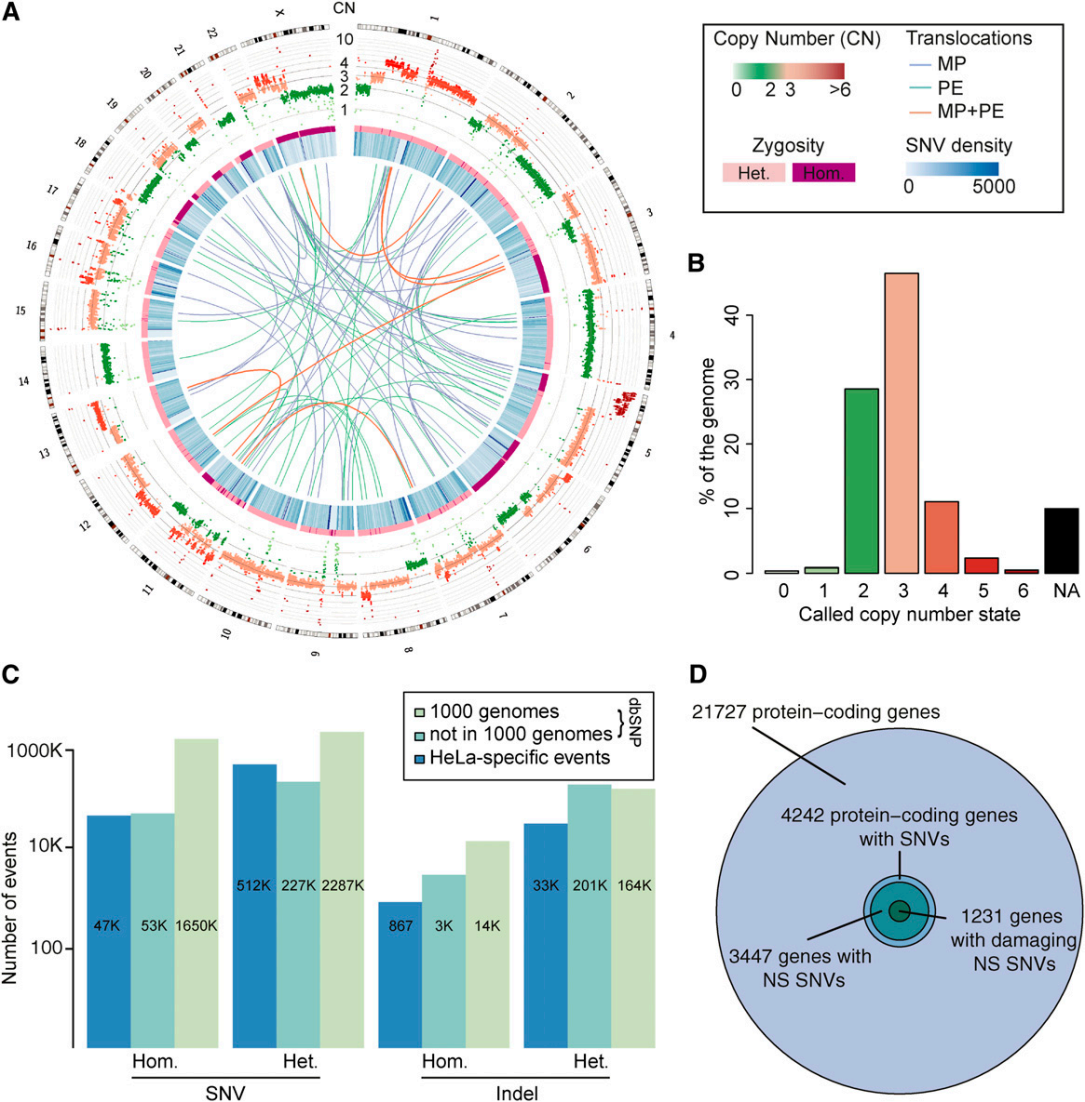
The genomic landscape of a HeLa cell line. (A) Circos plot (Krzywinski *et al.* 2009) of the HeLa genome with tracks representing read depth (100 kb-binned coverage), CN (color gradient from light green for CN1 to dark red for CN10), zygosity (pink: heterozygous; purple: homozygous), SNV density (1-Mb binned SNV count; darker blue for greater density), and translocation calls (colored arcs based on paired-end sequencing data: light blue; mate pair data: light green; both datasets: orange). (B) Histogram of called CN across the genome in percent. CN 0 corresponds to coverage less than half of the expected value for CN 1. A CN value of “NA” means no call could be made with confidence ≥0.95 (see *Materials and Methods*). (C) Overview of sequence variation in HeLa. Numbers of SNV and indel calls in HeLa, classified by overlap with dbSNP and the 1000 Genomes Project (dbSNP137). The y-axis shows the counts on a logarithmic scale. The four different classes of events represented on the x-axis are homozygous (“Hom.”) and heterozygous (“Het.”) SNVs and indels. (D) Variation observed in HeLa protein-coding genes relative to the human reference. Number of protein-coding genes containing SNVs, nonsynonymous SNVs, and damaging non-synonymous mutations [predicted by SIFT (Ng and Henikoff 2003)].

To confirm the identity of the analyzed cell line, we performed short-tandem repeat genotyping, which revealed a correspondence of >80% of the markers tested. With the identity of the cell line confirmed as HeLa, we proceeded to characterize the HeLa Kyoto genome. DNA sequencing produced about 1 billion reads of length 101 nt, of which 86% were aligned to the human reference (GRCh37). We identified extensive genetic variation, including SNVs and SVs, in the HeLa Kyoto genome compared with the human reference. A genome-wide representation of our results, including a Circos plot, is in Figure 1 (Krzywinski *et al.* 2009). Our analysis reveals the extent and nature of the differences between the human reference genome and the HeLa genome.

Numerous CN changes and sequence modifications were observed at the single nucleotide level and in larger structural rearrangements; these variants are detailed in the following sections. We report a compendium of genomic variation (CN, SNVs, and SVs) as well as the first HeLa genome draft, which are available as VCF and FASTA files, respectively. By integrating the set of homozygous variant calls with the greatest confidence (SNVs, small indels as well as large deletions and insertions; Table 1) into the human reference genome (GRCh37), we constructed a HeLa genome sequence. We retained the overall chromosome structure of the reference genome and encoded CN aberrations in a separate file. We provide a tool to perform the translation of coordinates between GRCh37 and our HeLa reference and report our variant calls in both coordinate systems.

**Table 1:** Homozygous variants

Classes	Homozygous calls used in HeLa genome
SNVs	1,733,577
Large Deletions	748
Short Deletions	15,034
Short Insertions	3446

Summary table of high-quality homozygous calls (SNVs: GATK; large deletions: DELLY; short deletions and insertions: PINDEL) integrated into the human reference genome to build the HeLa genome.

#### CN by read depth analysis:

By inferring CN using sequencing read depth, we observed extensive CN heterogeneity across the HeLa genome, with most loci present in three copies (Figure 1, A and B). These results corroborate previous observations that the genome contains an unbalanced number of chromosomes (3n+) (Macville *et al.* 1999) while providing a high-resolution (10 kb) survey of CN state.

#### SNVs and indels:

We used the detected SNVs to infer allelic variability and potential functional consequences. We also identified small indels up to 50 bp. We detected 1,750,535 SNVs and 18,411 indels that were homozygous in HeLa, of which 97.3% and 95.3% were already reported in dbSNP, respectively (release 137). Among the calls described in dbSNP, 96.9% of SNVs and 82.6% of indels were also in the 1000 Genomes Project dataset (1000 Genomes Project Consortium *et al*. 2012) (Figure 1C). Most variants in these HeLa cells thus represent common variants in the human population. The remaining 53,121 variant calls are either specific to Henrietta Lacks, somatic mutations of the tumor, or arose during transformation and propagation of the cell line. The lack of samples from the original cancerous and non-malignant tissue makes it difficult to distinguish these possibilities.

In addition, extensive allelic variability exists in HeLa. We detected 3,026,053 heterozygous SNVs and 397,969 heterozygous indels. A total of 83.1% and 91.8%, respectively, were already reported in dbSNP. Among these, 91.0% of heterozygous SNVs and 44.9% of heterozygous indels were contained in the 1000 Genomes Project dataset and thus represent common variants.

For the HeLa-specific SNVs we performed an additional quality control step to reduce false-positive calls. This step was based on the analysis of the mutation signatures stratified by local coverage (Figure S1). We extracted a high-confidence set of calls having a local coverage between 10 and 60 that contained 60% (336,006 of 559,384) of all HeLa-specific SNV calls. This range of coverage corresponds to that expected for a CN between one and six, accounting for approximately 80% of positions in the genome. Thus, the coverage filter removed a large subset of HeLa-specific SNV calls in low (<10) and high (>60) coverage regions, with many of these calls likely to be false positives. The high-confidence HeLa-specific calls were submitted to dbGaP.

To predict the impact of these variants on protein function, we used SIFT (Ng and Henikoff 2003) on the complete as well as the filtered high-confidence HeLa-specific call set. Among the 4,553,210 filtered SNVs, 29,213 were in coding regions, and within this subset, 4740 were nonsynonymous (NS) mutations, 1411 of which were predicted to alter protein function (Figure 1D). These potentially damaging SNVs were found in 1231 genes. The Gene Ontology (GO) class “sensory perception of chemical stimulus” was enriched in this subset of genes. We propose two possible reasons for this enrichment. First, sensory pathways involved in responses to changes in the environment could mutate without consequences given the constant medium composition in cell culture; second, the selective pressure for fast-growing cells may have led to a constitutive activation of sensory pathways independent of external signals, which is a common mechanism in cancer (Hanahan and Weinberg 2011). The list of mutations predicted to have an effect on protein function is available as a table. Of the 336,006 HeLa-specific SNVs, 1410 were localized in protein-coding sequences; 233 of these were predicted to be NS, of which 71 were predicted to impact the function of 66 proteins. No GO term enrichment could be detected in this subset of genes.

**Figure 2:**
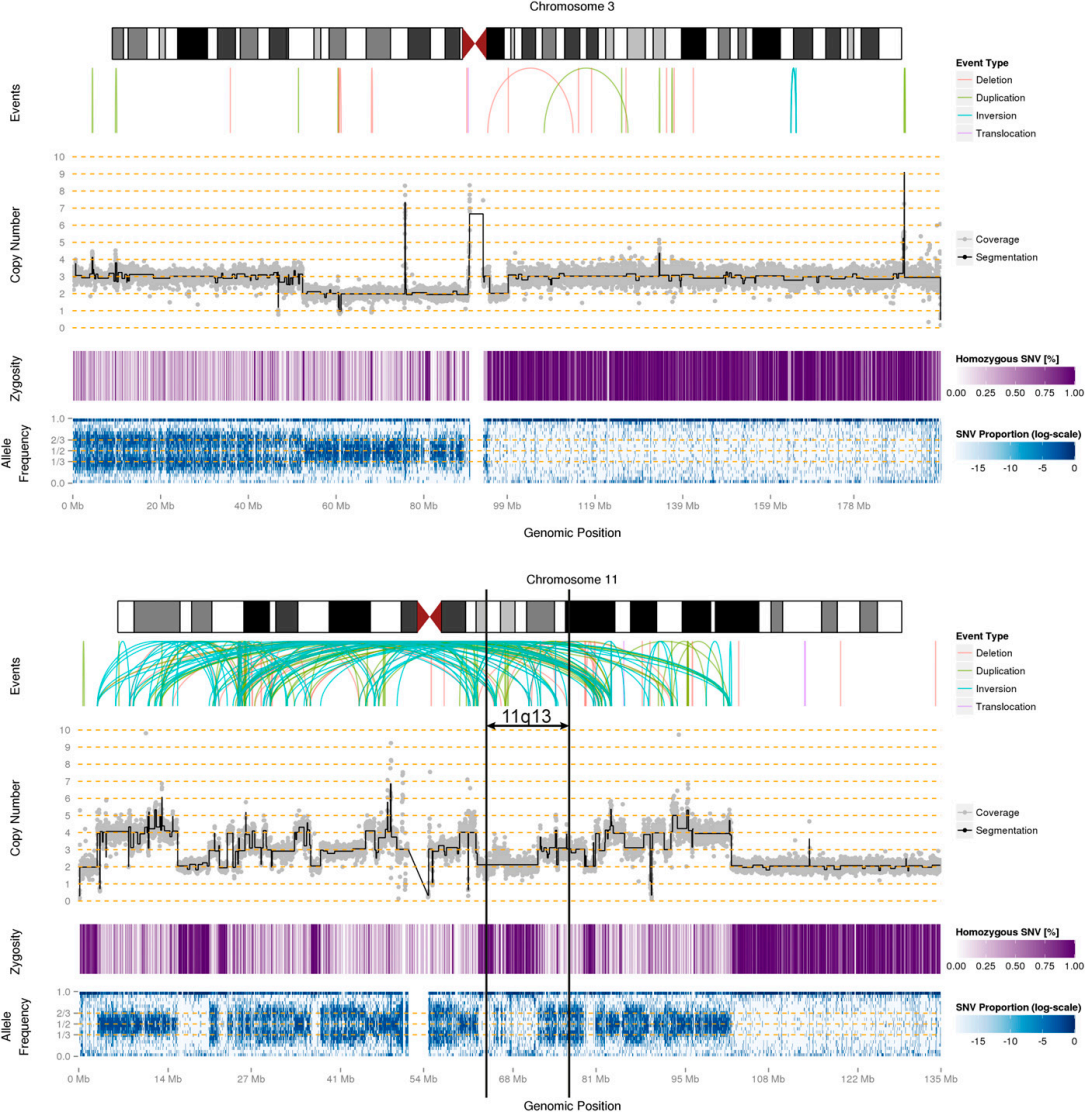
SVs, CN, zygosity and allele frequency along chromosomes 3 and 11. Arcs in the top panels labeled “Events” represent the predicted connections between fragments derived from SV calls based on read pair orientation and spacing. Different read pair signatures indicate the following event types: deletions, tandem duplications, inversions, and interchromosomal translocations. The center panel (“Copy Number”) represents the CN estimates in 10-kb bins (gray) overlaid with their segmentation (black). The associated CN is shown on the y-axis. The zygosity track shows the proportion of homozygous SNV calls in 10-kb bins; darker purple regions contain more homozygous calls (up to 100%) and indicate potential LOH. The bottom panel shows the allele frequency distribution as a heatmap in 10-kb bins on the chromosome axis and 5% bins on the allele frequency axis; darker blue indicates more SNVs with the given allele frequency in the corresponding 10 kb region. The color scale is according to the log of proportion of SNVs falling into the allele frequency bin (*e.g.*, 10–15%, *i.e.*, the row) in the 10 kb region (*i.e.*, the column). The chromosomal subregion 11q13, which is known to contain tumor-suppressor genes, is delineated with black bars.

Using the allele frequencies of our SNV calls, we created a classifier to identify homozygous regions of the genome (100-kb bins), which may have resulted from loss of heterozygosity (LOH). Overall, 23% of the genome was classified as homozygous (Figure 1A). It is important to note that less than 1% of this HeLa genome was classified as CN 1 (Figure 1B), and therefore the large majority of homozygous regions were present at CN ≥2. For comparison, the same analysis on individuals from the HapMap project (The International Hapmap Consortium 2003) did not reveal any homozygous regions larger than 100 kb in their autosomes. A large potential LOH region in HeLa chromosome 3 is depicted in detail along with allele frequencies, CN, coverage, SNVs, and rearrangements in Figure 2 (plots for all chromosomes are in Figure S2). Many of the homozygous segments in the HeLa genome correspond to previously reported LOH cervical cancer hotspots, namely on chromosomes 3p, 6p, 11q, and 18q (Mitra *et al.* 1994; Mullokandov *et al.* 1996; Rader *et al.* 1998; Koopman *et al.* 2000; Vermeulen *et al.* 2005; Corver *et al.* 2011). This finding suggests that these LOH events arose during the cervical cancer, prior to cultivation of the HeLa cell line.

Principal component analysis on the genotypes at known variant sites from 640 HapMap samples (phase 1−V3) representing eight different populations separated these samples by their population annotation and is consistent with the derivation of HeLa cells from an African-American individual (Figure S3). The African-American population (to which Henrietta Lacks belonged) is spread between the African and European clusters, with the HeLa sample overlapping both. This demonstrates that although the genomic landscape of HeLa is strikingly different from that of a normal human cell, the population-specific SNV patterns are still detectable.

#### Large SVs (>50 bp):

SV calls were made with the methods DELLY (Rausch *et al.* 2012a) (for deletions, inversions, tandem duplications, and translocations) and PINDEL (Ye *et al.* 2009) (for inversions and tandem duplications) using both paired-end (300-bp target insert size) and mate pair (4-kb target insert size) sequencing data. The two datatypes produce nucleotide sequences from both ends of a DNA fragment, the size of which differs depending on the technique used: paired-end reads effectively detect SVs as short as ∼100 bp, and mate pairs are suitable for identifying larger (> 1 kb) SVs.

We obtained breakpoints for 2891 SV calls, of which 2277 were supported by paired-end split reads and therefore at single-nucleotide resolution. A summary of all the SVs called using paired-end and mate pair data is found in Table 2.

**Table 2:** Extent of structural variation in HeLa

Classes	DELLY Calls Using PE Data		DELLY Calls Using MP Data		PINDEL Calls Using PE Data	
	No. Calls	Overlap, %	No. Calls	Overlap, %	No. Calls	Overlap, %
Deletions	1881	14.89	234	67.09	—	—
Duplications	312	38.78	191	69.63	591	22.00
Inversions	33	60.61	139	56.12	101	24.75
Translocations	51	9.80	50	10.00	—	—

Number of deletions, duplications, inversions, and translocations called by two methods (DELLY and PINDEL) using two different types of sequencing data: paired-end (PE) and mate pair (MP). For each category, the overlap is calculated as numbers of calls overlapping at least one of two other categories.

**Figure 3:**
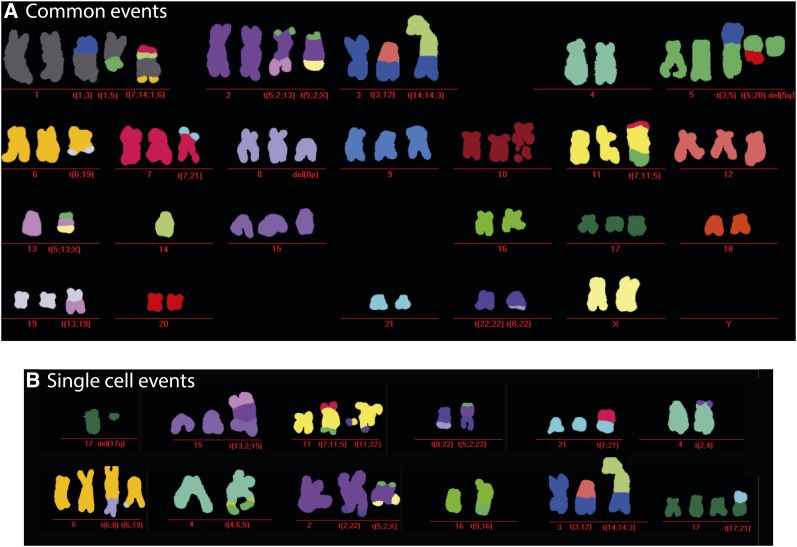
Colored HeLa karyotype by M-FISH. M-FISH results of 12 analyzed metaphase spreads identified a hypotriploid karyotype. The karyotype shown in (A) was derived from a single cell in which all aberrations were recurrent except for the one in chromosome 3. Single cell-specific events are shown in (B).

Among selected subsets of deletions (93), tandem duplications (52), inversions (3), and translocations (12), 35%, 50%, 100%, and 80% were validated by PCR, respectively. Multiplex fluorescent *in situ* hybridization (M-FISH) of 12 metaphase spreads revealed common rearrangements (Figure 3) as well as events that only occurred at the single-cell level. The average number of chromosomes per metaphase spread was 64, with a minimum of 62 and a maximum of 68. In addition, 20 large interchromosomal translocations were found in all 12 cells, 11 of which were also present in the translocation calls obtained from DNA sequencing data.

The results indicate that there is a core set of structural aberrations shared between the majority of cells in this population, as well as a set of rare events only observed in single cells. These rare events did not seem to manifest themselves in significant proportions of the population, since we did not find indications for the presence of subpopulations in the DNASeq data (*e.g.*, intermediate states in the depth-of-coverage or corresponding allele frequencies). A possible explanation is that *de novo* structural aberrations arise frequently, and in most cases disappear from the rapidly proliferating population because they confer a proliferative disadvantage.

#### Viral insertions:

Cervical cancer is often associated with genomic insertion of human papillomavirus, especially HPV16 and HPV18. We screened for potential viral insertion sites in the genome of this cell line and found an insertion of HPV18 on chromosome 8 (Table S1), which corroborates the previous characterization of the integration site of HPV18 (Macville *et al.* 1999). We detected 9 additional potential viral integration sites (Table S1).

#### Chromothripsis:

Massive rearrangements were observed on chromosomes 5, 19, X, and especially 11 (Figure 2). They displayed hallmarks of chromothripsis, including a high number of CN switches, alternations between a small number (2−3) of CN states, and high interconnectivity (*i.e.*, connections between regions which are usually far apart on a chromosome) (Stephens *et al.* 2011; Maher and Wilson 2012; Rausch *et al.* 2012b). Chromothripsis is a phenomenon observed in cancer cells, where parts of chromosomes are shattered and rearranged, seemingly at random. Chromothripsis has been associated with 2−3% of all cancers (Stephens *et al.* 2011) but to date has not been described in HeLa cells.

The complex intrachromosomal rearrangements on chromosome 11 have previously been observed at low resolution using cytogenetic analysis (DAPI and G-banding) (Macville *et al.* 1999). Furthermore, chromosome 11 presents indications of LOH according to our allelic distribution analysis (Figure 2). In previous studies where LOH on chromosome 11 was also observed in HeLa, the introduction of a functional copy of chromosome 11 suppressed the HeLa line’s characteristic aggressive proliferation phenotype (Oshimura *et al.* 1990). This indicates the presence of tumor suppressor genes on chromosome 11. LOH on this chromosome has also been observed in other cervical cancer cell lines (Stanbridge *et al.* 1981; Kaelbling & Klinger 1986; Saxon *et al.* 1986; Srivatsan *et al.* 1986; Koi *et al.* 1989). A potential cervical cancer-suppressor gene has been mapped to the 11q13 region (Srivatsan *et al.* 2002), which in HeLa displays rearrangements symptomatic of chromothripsis (Figure 2). Deletions and LOH within this region have also been associated with neuroblastoma as well as breast, head and neck, and nasopharynx cancers (Srivatsan *et al.* 1993; Zhuang *et al.* 1995; Chakrabarti *et al.* 1998; Tanaka *et al.* 1998; Venugopalan *et al.* 1998; Cheng *et al.* 2002). It is therefore possible that chromothripsis and LOH on chromosome 11 contributed to the development of Henrietta Lacks’ cervical cancer.

### Transcriptomic landscape

To characterize HeLa gene expression, we sequenced polyadenylated RNAs from HeLa cells, producing 450 million reads of lengths 76 or 105 nt. 56% of the total read number (253 million) were aligned to the HeLa genome sequence described above.

#### Expression level and CN:

**Figure 4:**
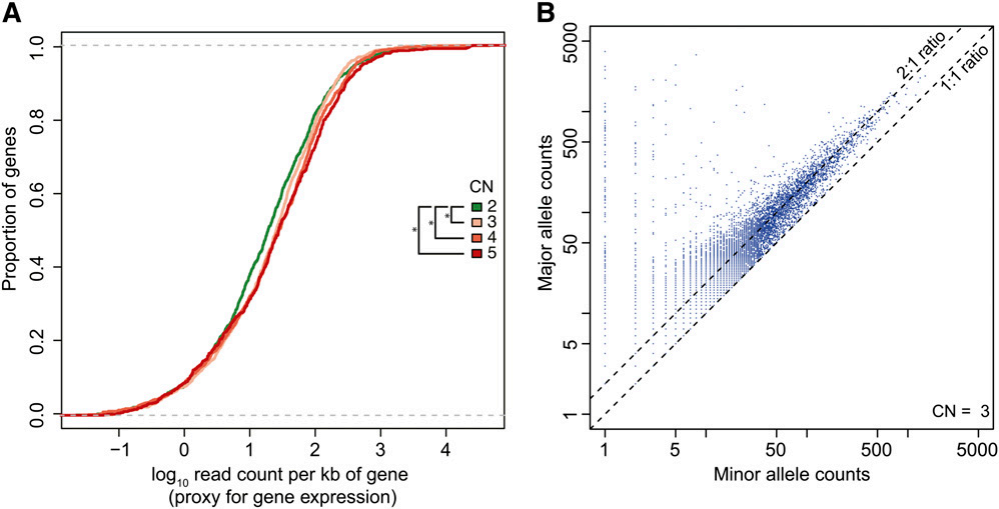
A general lack of dosage compensation observed in HeLa gene expression. (A) Correlation between CN and gene expression levels. Empirical cumulative distribution functions of gene expression values (for genes detected as expressed), grouped by CN state of the region containing the gene. The x-axis shows the logarithm (base 10) of read counts per kb of gene and the y-axis shows the corresponding cumulative distribution function. Significance (*) was calculated by the Wilcoxon test (*P* < 0.01). (B) Lack of allele-specific dosage compensation. For each SNV in genome segments of CN 3, the higher and lower RNA-Seq read count for both alleles are shown (higher count on the y-axis, lower count on the x-axis). The two dashed lines represent ratios 2:1 and 1:1. The observed ratios center around the 2:1 line, indicating an overall lack of allele-specific dosage compensation.

We investigated the relationship between gene expression levels and CN, observing a significant overall increase in expression levels as a function of gene CN (Figure 4; Wilcoxon test *P* < 0.01), especially among highly expressed genes (read counts per kb of gene ≥100). Using the variant calls overlapping regions of CN 3 obtained in the genomic analysis, we determined the allelic expression from the transcriptomic data. If dosage compensation were achieved by silencing one of the three copies of each gene, allelic expression ratios of 1:1 or 2:0 would be expected. However, we found that overall, an allelic ratio of 2:1 was maintained in the RNA expression data (Figure 4B). These data suggest that neither allele-specific nor non-allele-specific dosage compensation effects occur on a genome-wide scale, although certain genes may be subject to these effects.

#### Comparing the transcriptomes of HeLa, human tissues, and human cell lines:

We compared the gene expression profile of HeLa with other cell lines and tissues, using data from ENCODE cell lines (Djebali *et al.* 2012) (ENA number: SRP014320) and the Illumina Human BodyMap 2.0 (ENA number: ERP000546). By comparing the normalized gene expression levels of the 16 tissues from the BodyMap with those from our HeLa RNAseq library, we identified 1907 genes (of which 805 are protein-coding) that were more highly expressed in HeLa than in any tissue in the BodyMap. Using the MGSA algorithm (Bauer *et al.* 2011) to detect overrepresented functional categories, we identified GO terms (Ashburner *et al.* 2000) related to functions that are plausibly beneficial to this cell line, such as proliferation (cell cycle phase), transcription (RNA processing, rRNA transcription), and DNA repair (Table 3). Notably, the highly expressed DNA repair pathways each contained at least one component predicted to be nonfunctional by our SIFT analysis of variation in their protein sequences. These observations suggest that HeLa cells sense the damage occurring to their DNA and activate pathways to repair or minimize this damage, even though mutations in some components may render this response ineffective.

**Table 3:** Gene Ontology (GO) term enrichment for highly expressed genes in HeLa

GO ID	GO Term (Biological Process)	Total Genes	Number of Highly Expressed Genes	Posterior Probability	SD
GO:0006281	DNA repair	382	75	0.93	0.01
GO:0022403	cell cycle phase	807	163	0.73	0.03
GO:0006396	RNA processing	667	89	0.70	0.01
GO:0009303	rRNA transcription	19	5	0.52	0.00

Remarkably, 23,966 genes, of which 5593 are protein-coding, were not detected as expressed in HeLa. GO terms enriched in this subset were related to response to stimulus (defense response, immune system response, G-protein coupled receptor signaling pathway), protein cleavage (proteolysis, lipid catabolic process), and specific biological functions such as sexual reproduction, central nervous system development, and epidermis development (Table 4). That the expression of this large number of genes was not detected in HeLa could reflect their lack of expression in the original cervical tissue or loss of expression during either cancer progression or cell culture. Of these genes, 13392 (1722 protein-coding) were also not detectable in data from 14 cell lines used in the ENCODE project (Figure S6A). This suggests that these genes are not required for standard cell culture and their inactivation is unrelated to cancer or tissue-specificity, because these cell lines originate from various cancerous and normal human tissues. Clustering of the 15 cell lines based on their transcriptomic pattern showed separation of non-cancer and cancer cell lines, as well as strong similarity between the two HeLa cell lines—the ENCODE set included HeLa S3, which grows in suspension unlike Kyoto, an adherent cell line (Figure S6B). This finding indicates that despite their clear phenotypic differences, the two HeLa lines’ transcriptomes are more similar to each other than to other cell lines.

**Table 4:** Gene Ontology (GO) term enrichment for genes with undetected expression in HeLa

GO ID	GO Term (Biological Process)	Total genes	No. Undetected Genes	Posterior Probability	SD
GO:0007186	G-protein coupled receptor signaling pathway	1148	668	0.60	0.16
GO:0006952	defense response	1127	356	0.60	0.16
GO:0006955	immune response	1159	325	0.59	0.16
GO:0006508	proteolysis	964	220	0.59	0.16
GO:0016042	lipid catabolic process	229	64	0.56	0.15
GO:0019953	sexual reproduction	555	152	0.53	0.14
GO:0007417	central nervous system development	646	158	0.52	0.14
GO:0008544	epidermis development	270	80	0.51	0.14

#### Design and interpretation of RNAi:

Validating RNAi screen results is one potential application of the dataset provided in this study. Most RNAi designs, particularly the commercially available ones, are based on the human reference genome (Mazur *et al.* 2012). We used our data to reanalyze some of the results of the MitoCheck project, which performed high-throughput RNAi in the same HeLa cell line to screen for genes involved in chromosome segregation and cell division (Neumann *et al.* 2010). Knockdown of the gene *CABP7* (www.mitocheck.org; MCG_0016344) was shown by Neumann *et al.* (2010) to induce a mitotic defect phenotype. The specificity of this phenotype was validated by rescue with a mouse transgene. *CABP7* was targeted by four siRNAs, of which only two induced the defect. In our HeLa transcriptome data, we found that only these two siRNAs matched expressed sequence from the *CABP7* locus, whereas the two non-phenotype-inducing siRNAs did not match any reads. We anticipate that the genomic and transcriptomic data for HeLa might be useful for further, analogous reanalyses of data, and could inform RNAi design for future experiments.

## Discussion

Since the establishment of the HeLa cell line in 1951, it has been used as a model for numerous aspects of human biology with only minimal knowledge of its genomic properties. Here we provide the first detailed characterization of the genomic landscape of one HeLa line relative to the human reference genome. We integrated SNVs, deletions, inversions, tandem duplications, and CN changes along the genome to build a HeLa Kyoto genome. This provides a resource for the community, for instance, to inform primer or RNAi design. In addition, we provide high-resolution RNA-Seq data of the HeLa transcriptome and analyze them based on this cell line’s genome sequence.

We studied the relationship between CN variation and expression. CN is expected to impact gene expression levels in a proportional manner unless dosage compensation occurs (Aït Yahya-Graison *et al.* 2007; Deng and Disteche 2010). Our results showed that for genes present at the most prevalent CN state of 3, there is no general evidence of allele-specific dosage compensation and that general compensation, if active, is not strong. This finding corroborates observations that Schlattl *et al.* (2011) have made in lymphoblastoid cell lines assessing polymorphic deletions. A lack of dosage compensation could impact the function of genes in protein complexes, where the stoichiometry of complex members is affected by CN changes.

We identified approximately 4.5 million SNVs and 0.5 million indels, in addition to ~3000 SVs, including deletions, insertions, and interchromosomal translocations (Table 2). More than 80% of these SNVs and short indels are most likely common variants segregating in the human population, since they are also present in SNV catalogs such as dbSNP (Sherry *et al.* 2001) and the 1000 Genomes Project dataset (1000 Genomes Project Consortium *et al*. 2012). The remaining variants likely comprise rare, tumor-specific, or cell-line-specific variants.

A particularly striking genomic property that we discovered in HeLa cells is chromothripsis (Stephens *et al.* 2009) (Figure 2). Chromothripsis has been associated with 2–3% of all cancers, and examples have been described in many different cancer types (Bass *et al.* 2011; Berger *et al.* 2011; Kloosterman *et al.* 2011; Magrangeas *et al.* 2011; Rausch *et al.* 2012b). It could be present in HeLa for several reasons. One possibility is that this massive set of rearrangements itself triggered carcinogenesis (Maher and Wilson 2012). Another possibility is that chromothripsis occurred *in vitro* during cultivation of the cell line.

Our HeLa transcriptome data showed that close to 2000 genes are expressed higher than the physiological range of 16 human tissues. The functions enriched among these genes are related to proliferation, transcription, and DNA repair. The high expression of some DNA repair genes, some of which also carry potentially damaging NS mutations, suggests that even though HeLa displays high chromosomal instability, specific DNA repair mechanisms may be activated, perhaps irrespective of their effectiveness.

Our analysis is based on shotgun sequencing data of a HeLa cell line at moderate depth. Such data have specific limitations, in particular for phasing of distant variants (*i.e.*, identifying variants co-occurring on a single chromosome) and detection of SVs affecting repetitive regions. These limitations could be overcome by additional data derived from, for example, fosmid libraries, chromosome separation, or large-scale mate pair libraries, although these experiments would be more costly and time-consuming. Here we focused on localized variants that are detectable from shotgun data, which already provide wide-ranging insights into the genomic landscape of HeLa. We expect that in future, researchers working with cell lines will routinely characterize the genomes of their lines. When the genomes of cell lines are unstable, such as for HeLa, the characterization might need to be regularly updated. We envisage that approaches similar to the one taken here might help ensure the integrity of cell lines and the quality of the biological insights derived from them.

## Supplementary Material

Supporting Information

Publisher's Statement 26 March 2013

Landry_EMBL Statement_Data 19 March 2013
