# Supplementary material for: The Genomic and Transcriptomic Landscape of a HeLa Cell Line
Source: G3 (Bethesda). 2013 Mar 11;3(8):1213–24. doi: 10.1534/g3.113.005777 (PMC3737162; doi:10.1534/g3.113.005777)
Supplement: Supporting Information [file supp_3_8_1213__index.html]

The Genomic and Transcriptomic Landscape of a HeLa Cell Line — Supporting Information 

# The Genomic and Transcriptomic Landscape of a HeLa Cell Line

## Supporting Information for Landry *et al.*, 2013

**Files in this Data Supplement:**

- Supporting Information - Figures S1-S6 and Table S1 (PDF, 3.4 MB)
- Figure S1 - Mutational spectra in the HeLa Kyoto genome (PDF, 186 KB)
- Figure S2 - Structural variants, copy number and loss of heterozygosity for chromosomes 1 to 22 and X (PDF, 2.6 MB)
- Figure S3 - Principal component analysis of SNVs in HeLa Kyoto and 640 HapMap individuals from 8 different populations (PDF, 178 KB)
- Figure S4 - Effect of GC adjustment on sequencing coverage (PDF, 354 KB)
- Figure S5 - Primer design for detection of genomic rearrangements (PDF, 170 KB)
- Figure S6 - Comparison of HeLa transcriptome profile to Illumina Body Map tissues and ENCODE cell lines (PDF, 264 KB)
- Table S1 - Potential viral insertions (PDF, 86 KB)
