# Supplementary material for: The Genomic and Transcriptomic Landscape of a HeLa Cell Line
Source: G3 (Bethesda). 2013 Mar 11;3(8):1213–24. doi: 10.1534/g3.113.005777 (PMC3737162; doi:10.1534/g3.113.005777)
Supplement: Supporting Information [file supp_g3.113.005777_005777SI.pdf]

G3·Genes | Genomes | Genetics

The genomic and transcriptomic landscape of a HeLa cell line

Author list:

Jonathan Landry*[#], Paul Theodor Pyl*[#], Tobias Rausch*, Thomas Zichner*, Manu M. Tekkedil*, Adrian M. Stütz*, Anna Jauch§, Raeka S. Aiyar*, Gregoire Pau*[1], Nicolas Delhomme*[2], Julien Gagneur*[3], Jan O. Korbel*, Wolfgang Huber*, Lars M. Steinmetz*

Affiliations:

* European Molecular Biology Laboratory, Genome Biology Unit, Heidelberg, 69117 Germany

§ University Hospital Heidelberg, Institute of Human Genetics, Heidelberg, 69120 Germany

DOI: 10.1534/g3.113.005777

# These authors contributed equally to this work.
[1] Department of Bioinformatics and Computational Biology, Genentech Inc., South San Francisco, California, 94080
[2] Department of Plant Physiology, Umeå Plant Science Center, Umeå, Sweden, S-901 87
[3] Department of Chemistry and Biochemistry, Ludwig-Maximilians-Universität München, Munich, Germany, 81377

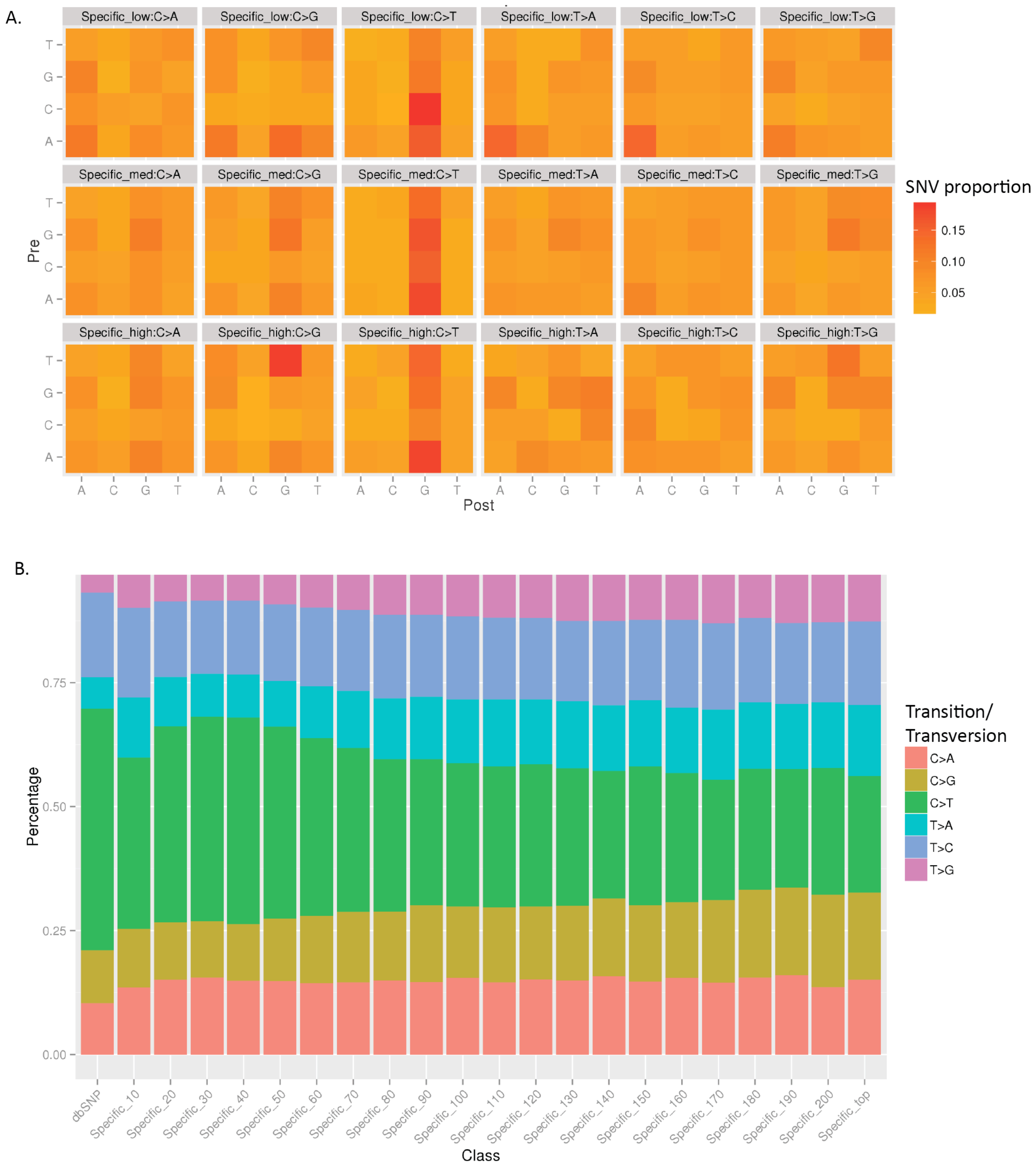

Figure S1 Mutational spectra in the HeLa Kyoto genome.
(A) Heatmap representation of mutational spectra observed in HeLa Kyoto specific calls stratified by local coverage (low: < 10; med: >=10 and < 60; high: >= 60). Each row corresponds to one group of SNVs and each column to a type of mutation (e.g.: C>A). Each heatmap shows the observed proportion of SNVs for all possible combinations of preceding ('Pre') and following ('Post') bases.
(B) Stacked barplots of the distribution of mutation types (e.g.: C>T) in observed HeLa specific SNVs, stratified by local coverage: Column "Specific_x" represents calls with coverage between x-10 and x. Column "dbSNP" shows called SNVs that have dbSNP ids, column "Specific_top" shows calls with local coverage greater than 200.

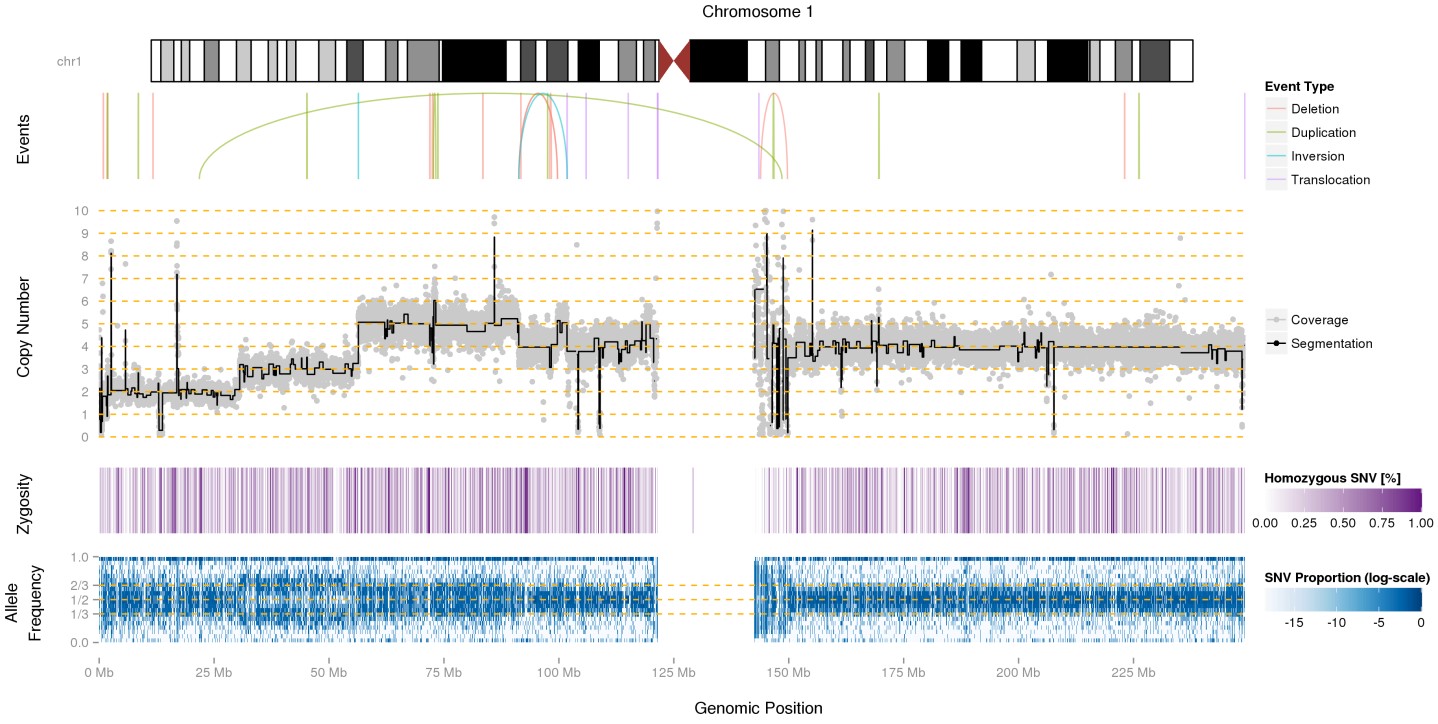
Chromosome 1
chr1
Events
Event Type
Deletion
Duplication
Inversion
Translocation
Copy Number
Coverage
Segmentation
Zygosity
Homozygous SNV [%]
0.00 0.25 0.50 0.75 1.00
Allele Frequency
SNV Proportion (log-scale)
-15 -10 -5 0
0 Mb 25 Mb 50 Mb 75 Mb 100 Mb 125 Mb 150 Mb 175 Mb 200 Mb 225 Mb
Genomic Position

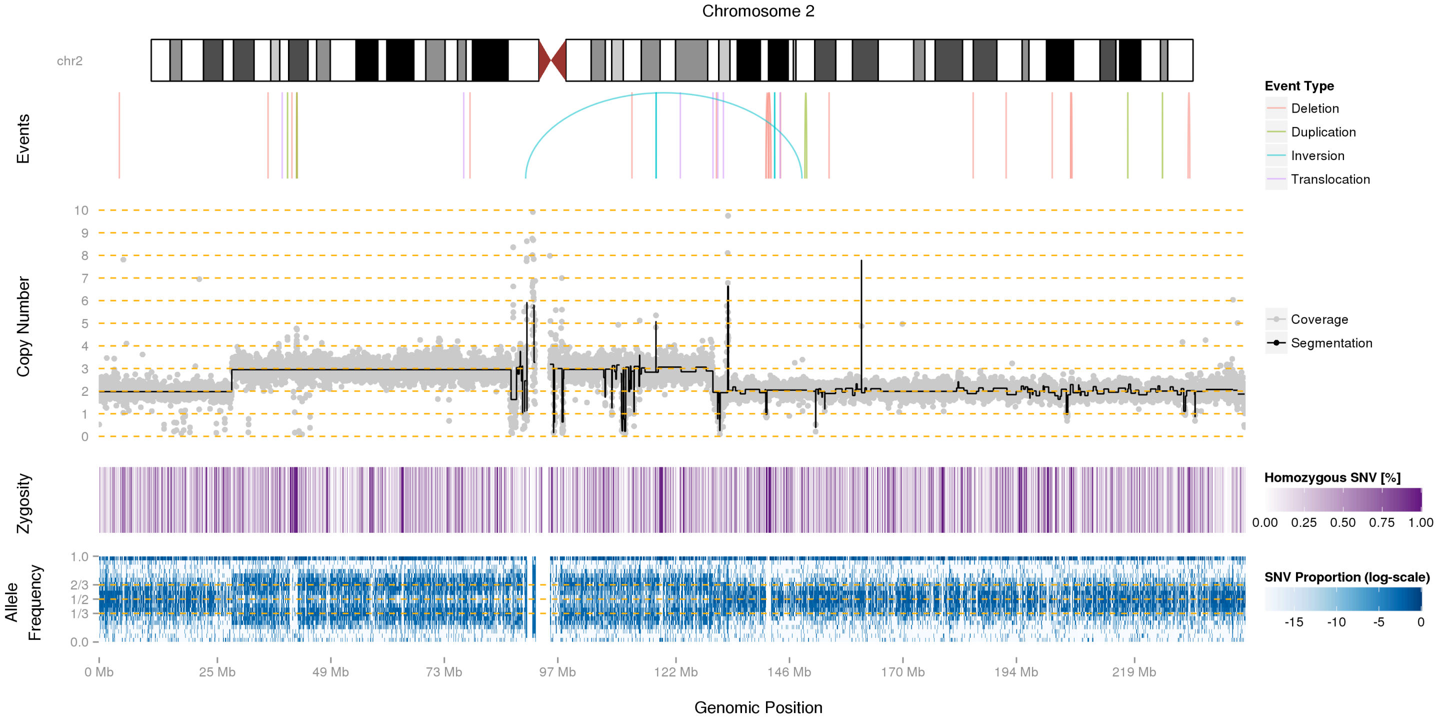
Chromosome 2
chr2
Events
Event Type
Deletion
Duplication
Inversion
Translocation
Copy Number
Coverage
Segmentation
Zygosity
Homozygous SNV [%]
0.00 0.25 0.50 0.75 1.00
Allele Frequency
SNV Proportion (log-scale)
-15 -10 -5 0
0 Mb 25 Mb 49 Mb 73 Mb 97 Mb 122 Mb 146 Mb 170 Mb 194 Mb 219 Mb
Genomic Position

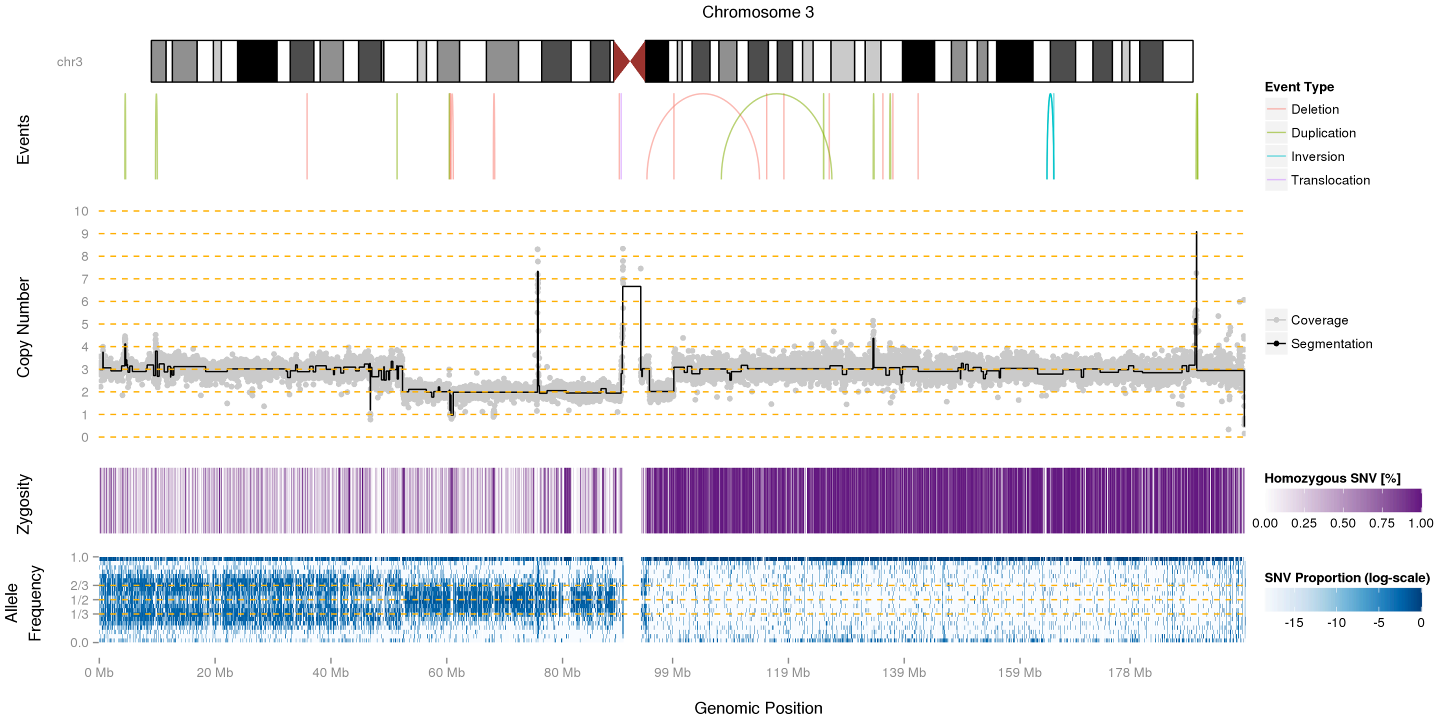
Chromosome 3
chr3
Events
Copy Number
Zygosity
Allele Frequency
Event Type
Deletion
Duplication
Inversion
Translocation
Coverage
Segmentation
Homozygous SNV [%]
0.00 0.25 0.50 0.75 1.00
SNV Proportion (log-scale)
-15 -10 -5 0
0 Mb 20 Mb 40 Mb 60 Mb 80 Mb 99 Mb 119 Mb 139 Mb 159 Mb 178 Mb
Genomic Position

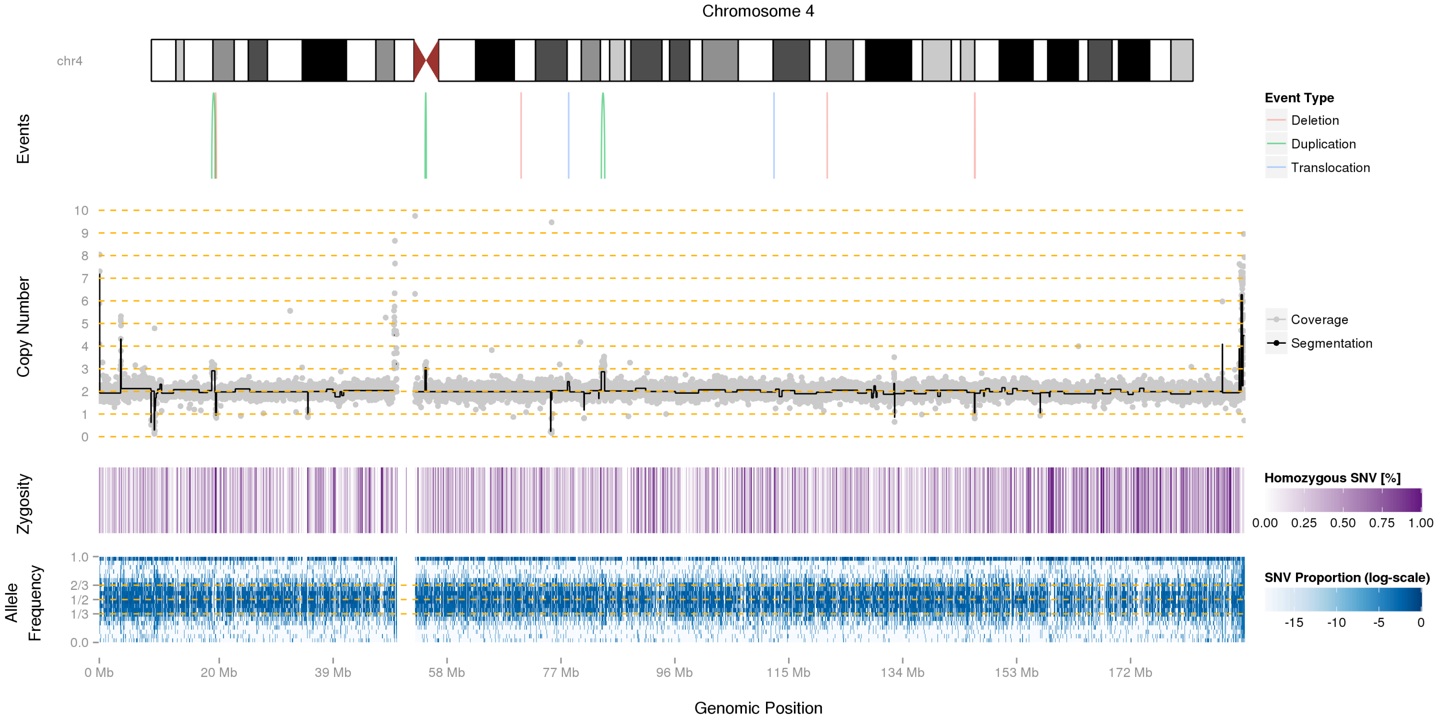
Chromosome 4
chr4
Events
Copy Number
Zygosity
Allele Frequency
Event Type
Deletion
Duplication
Translocation
Coverage
Segmentation
Homozygous SNV [%]
0.00 0.25 0.50 0.75 1.00
SNV Proportion (log-scale)
-15 -10 -5 0
0 Mb 20 Mb 39 Mb 58 Mb 77 Mb 96 Mb 115 Mb 134 Mb 153 Mb 172 Mb
Genomic Position

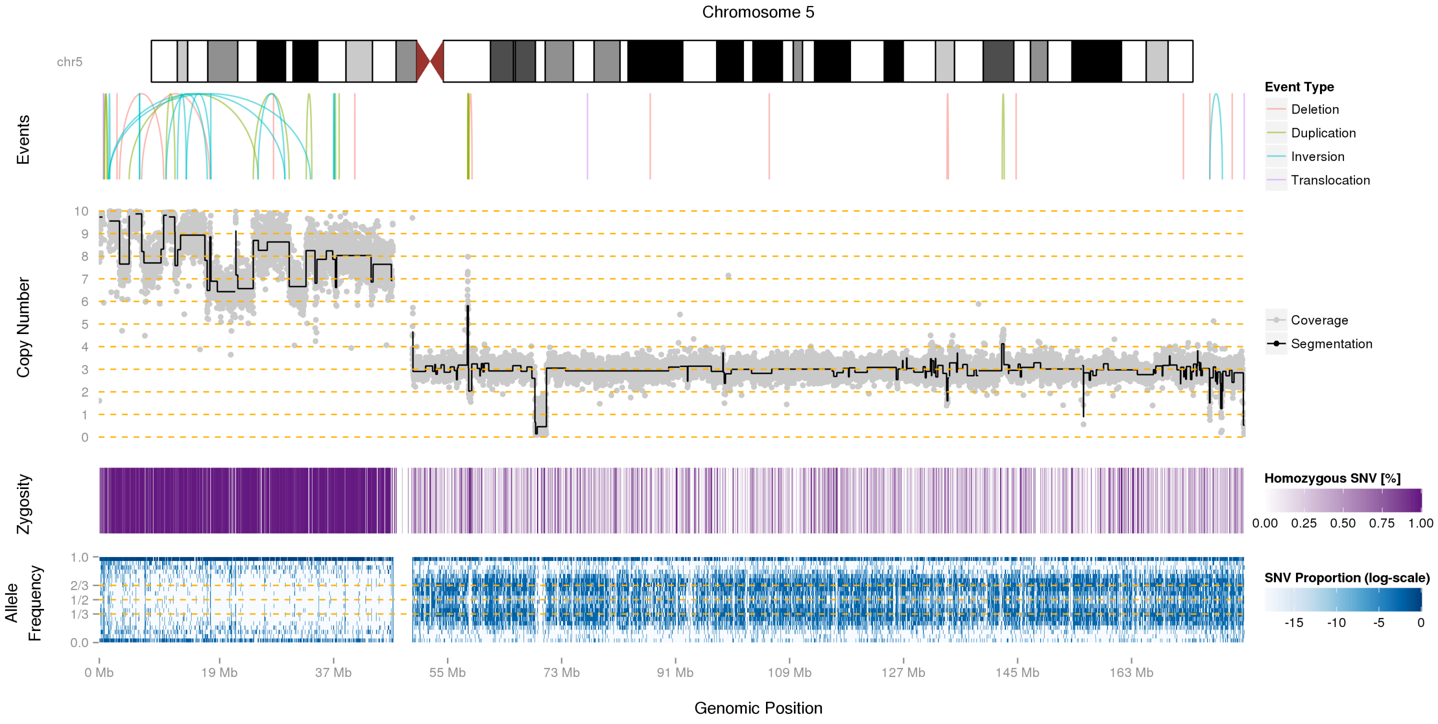
Chromosome 5
chr5
Events
Event Type
Deletion
Duplication
Inversion
Translocation
Copy Number
10
9
8
7
6
5
4
3
2
1
0
Coverage
Segmentation
Zygosity
Homozygous SNV [%]
0.00 0.25 0.50 0.75 1.00
Allele Frequency
1.0
2/3
1/2
1/3
0.0
SNV Proportion (log-scale)
-15 -10 -5 0
0 Mb 19 Mb 37 Mb 55 Mb 73 Mb 91 Mb 109 Mb 127 Mb 145 Mb 163 Mb
Genomic Position

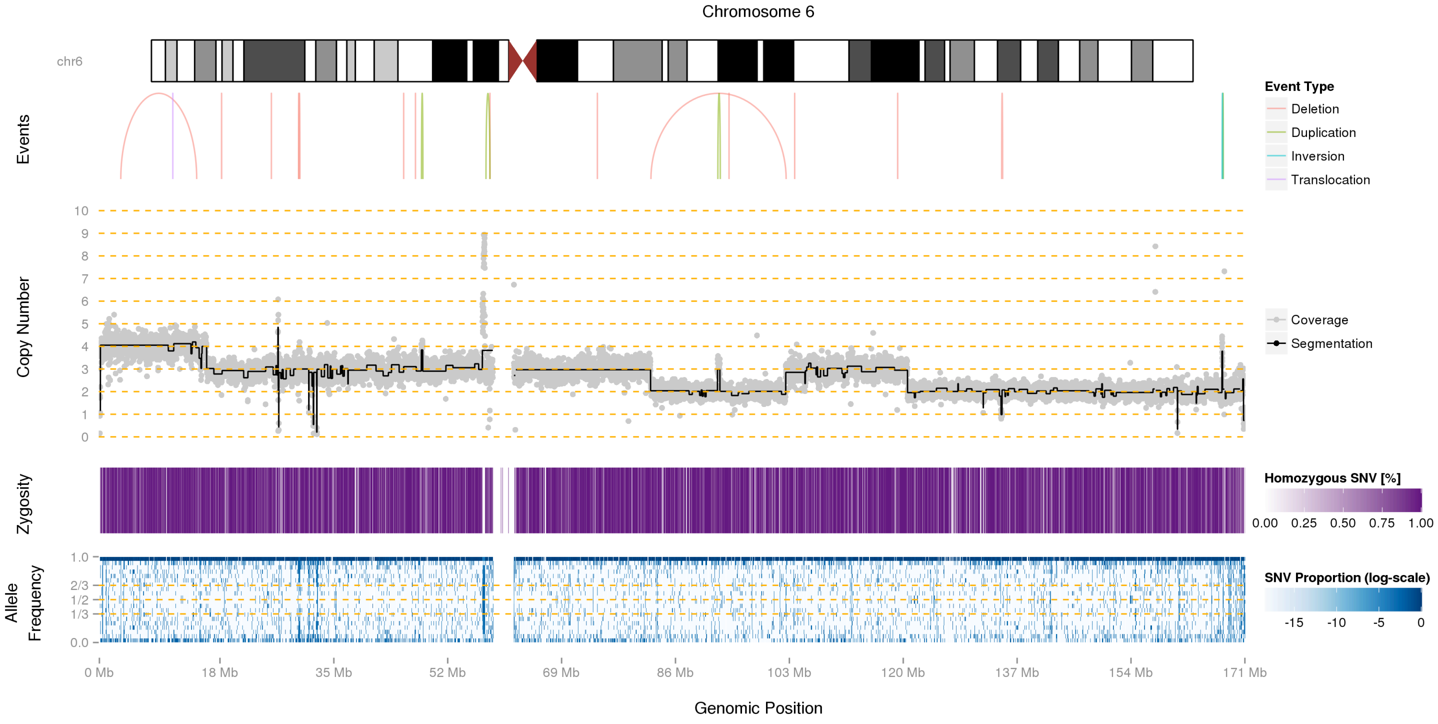
Chromosome 6
chr6
Events
Event Type
Deletion
Duplication
Inversion
Translocation
Copy Number
10
9
8
7
6
5
4
3
2
1
0
Coverage
Segmentation
Zygosity
Homozygous SNV [%]
0.00 0.25 0.50 0.75 1.00
Allele Frequency
1.0
2/3
1/2
1/3
0.0
SNV Proportion (log-scale)
-15 -10 -5 0
0 Mb 18 Mb 35 Mb 52 Mb 69 Mb 86 Mb 103 Mb 120 Mb 137 Mb 154 Mb 171 Mb
Genomic Position

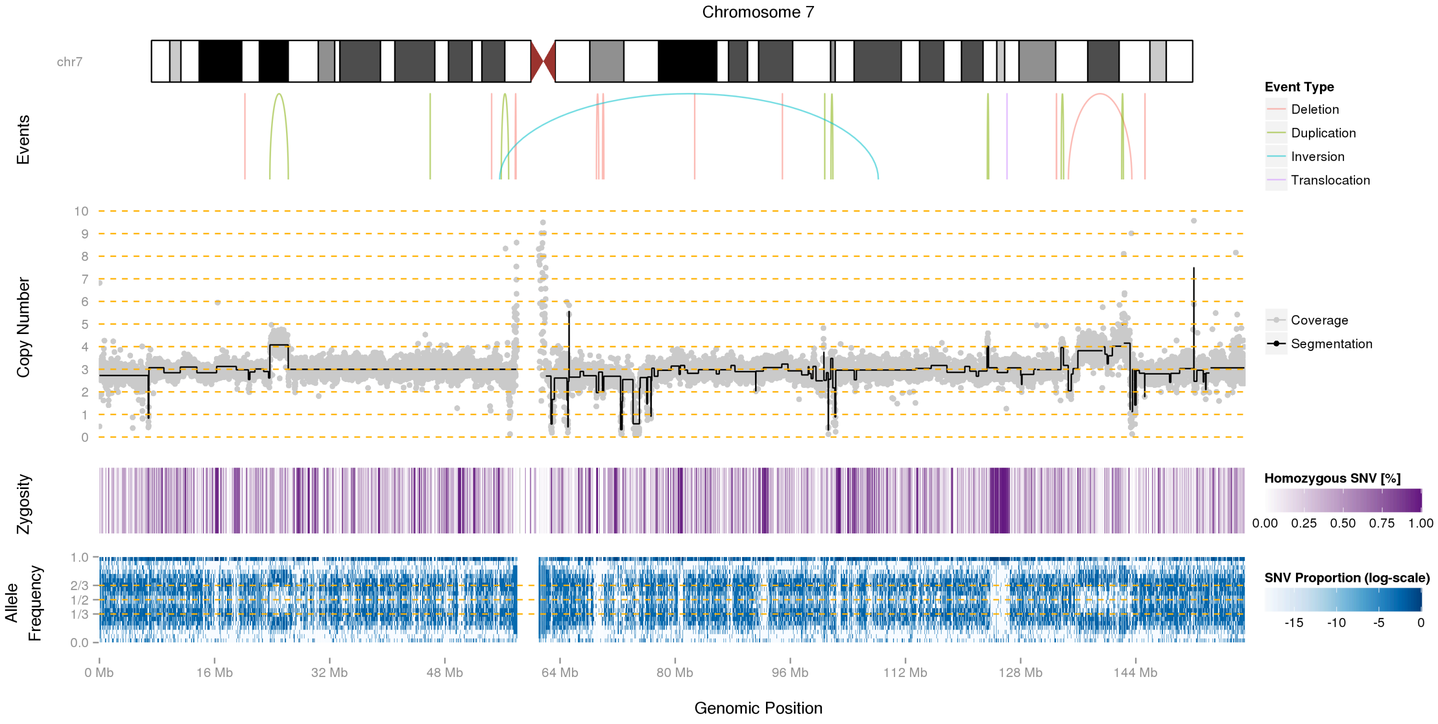
Chromosome 7
chr7
Events
Event Type
Deletion
Duplication
Inversion
Translocation
Copy Number
10
9
8
7
6
5
4
3
2
1
0
Coverage
Segmentation
Zygosity
Homozygous SNV [%]
0.00 0.25 0.50 0.75 1.00
Allele Frequency
1.0
2/3
1/2
1/3
0.0
SNV Proportion (log-scale)
-15 -10 -5 0
0 Mb 16 Mb 32 Mb 48 Mb 64 Mb 80 Mb 96 Mb 112 Mb 128 Mb 144 Mb
Genomic Position

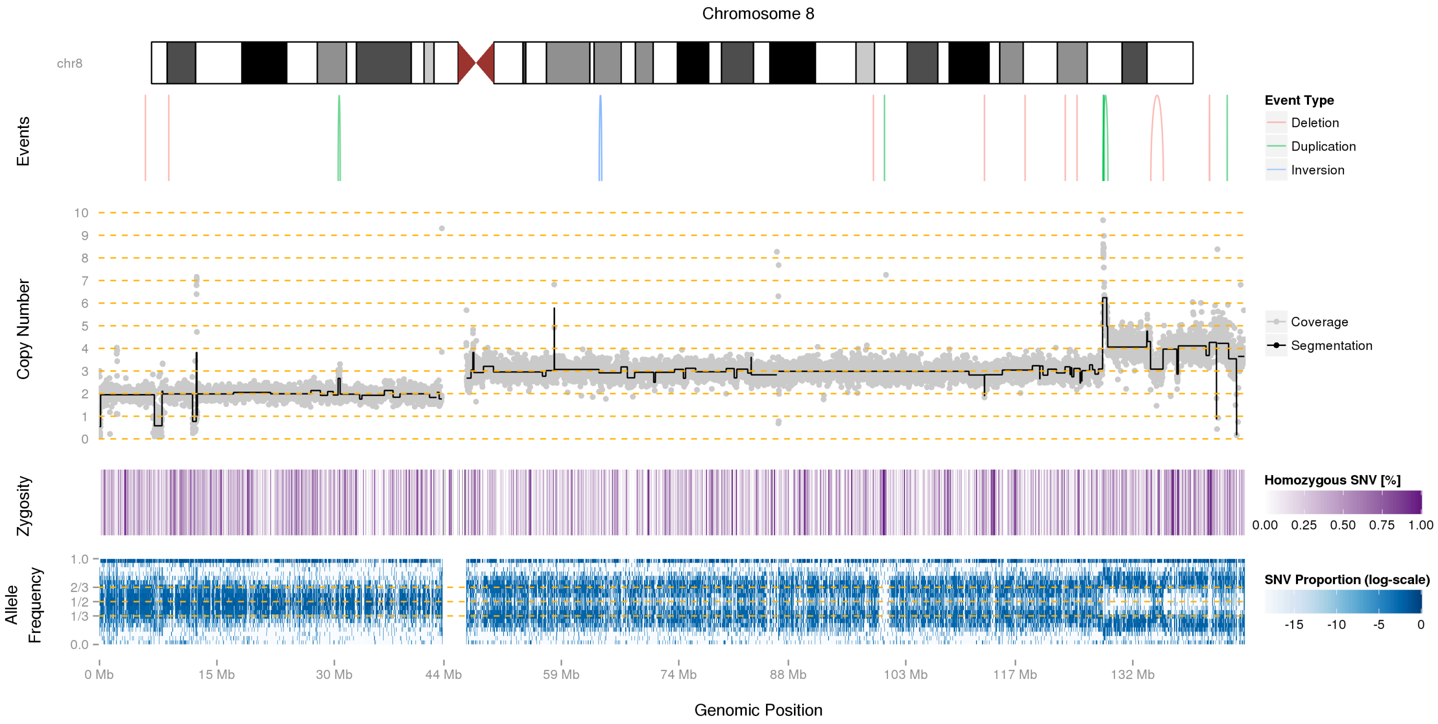
Chromosome 8
chr8
Events
Event Type
Deletion
Duplication
Inversion
Copy Number
10
9
8
7
6
5
4
3
2
1
0
Coverage
Segmentation
Zygosity
Homozygous SNV [%]
0.00 0.25 0.50 0.75 1.00
Allele Frequency
1.0
2/3
1/2
1/3
0.0
SNV Proportion (log-scale)
-15 -10 -5 0
0 Mb 15 Mb 30 Mb 44 Mb 59 Mb 74 Mb 88 Mb 103 Mb 117 Mb 132 Mb
Genomic Position

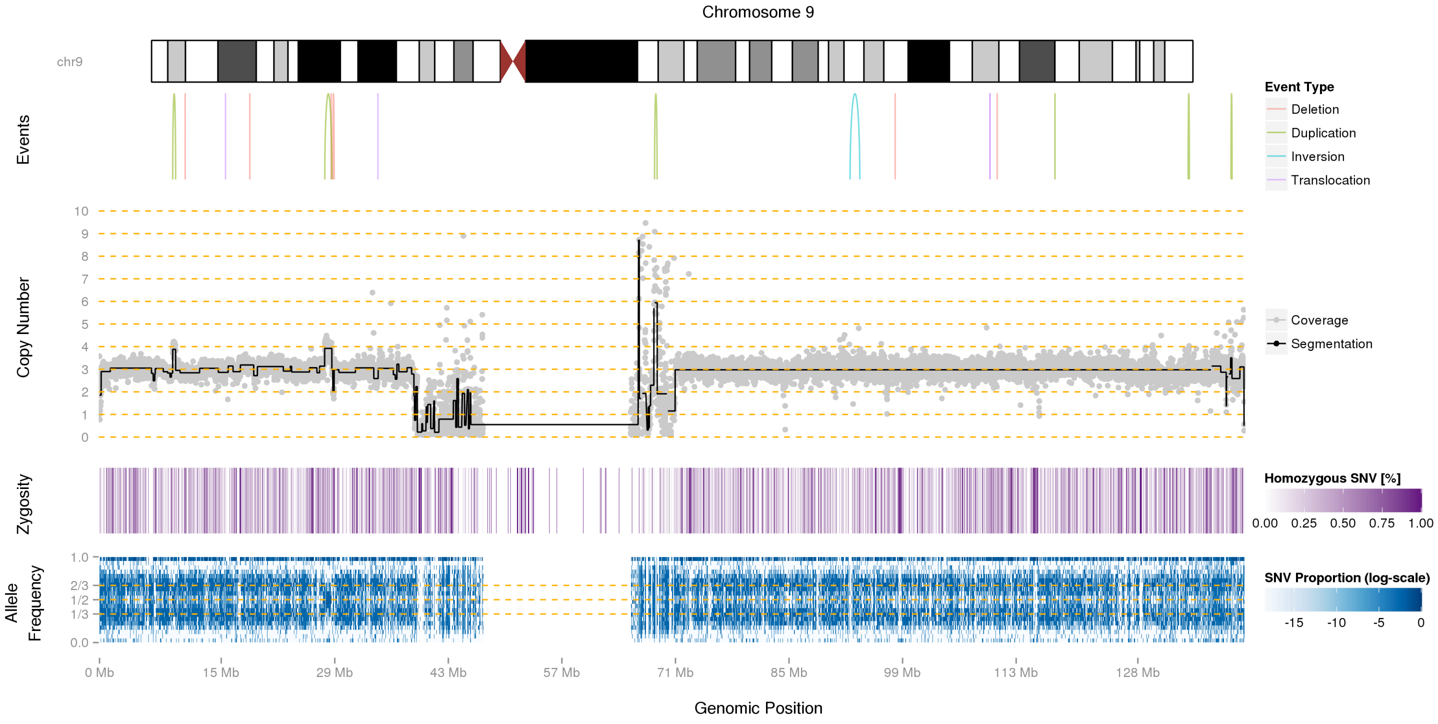

Chromosome 9
chr9
Events
Event Type
Deletion
Duplication
Inversion
Translocation
Copy Number
Coverage
Segmentation
Zygosity
Homozygous SNV [%]
0.00 0.25 0.50 0.75 1.00
Allele Frequency
SNV Proportion (log-scale)
-15 -10 -5 0
0 Mb 15 Mb 29 Mb 43 Mb 57 Mb 71 Mb 85 Mb 99 Mb 113 Mb 128 Mb
Genomic Position

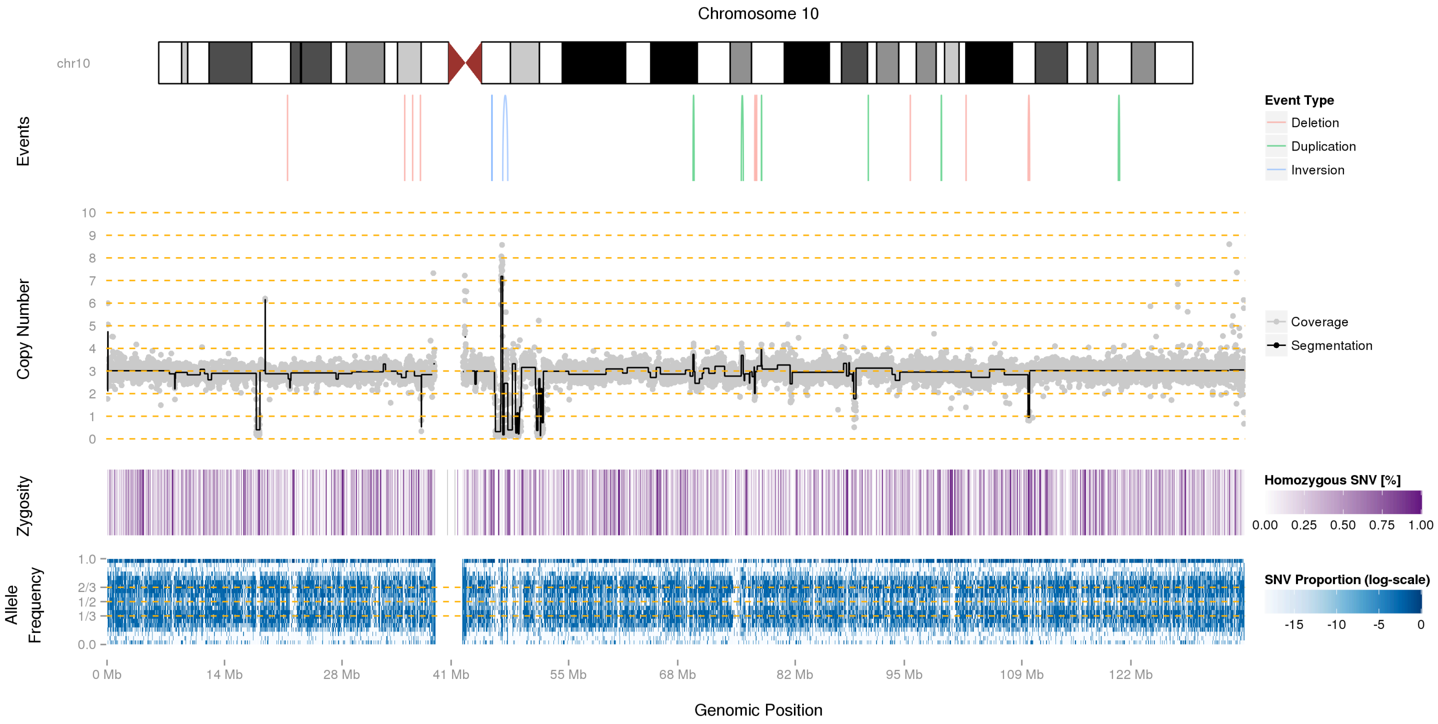

Chromosome 10
chr10
Events
Event Type
Deletion
Duplication
Inversion
Copy Number
Coverage
Segmentation
Zygosity
Homozygous SNV [%]
0.00 0.25 0.50 0.75 1.00
Allele Frequency
SNV Proportion (log-scale)
-15 -10 -5 0
0 Mb 14 Mb 28 Mb 41 Mb 55 Mb 68 Mb 82 Mb 95 Mb 109 Mb 122 Mb
Genomic Position

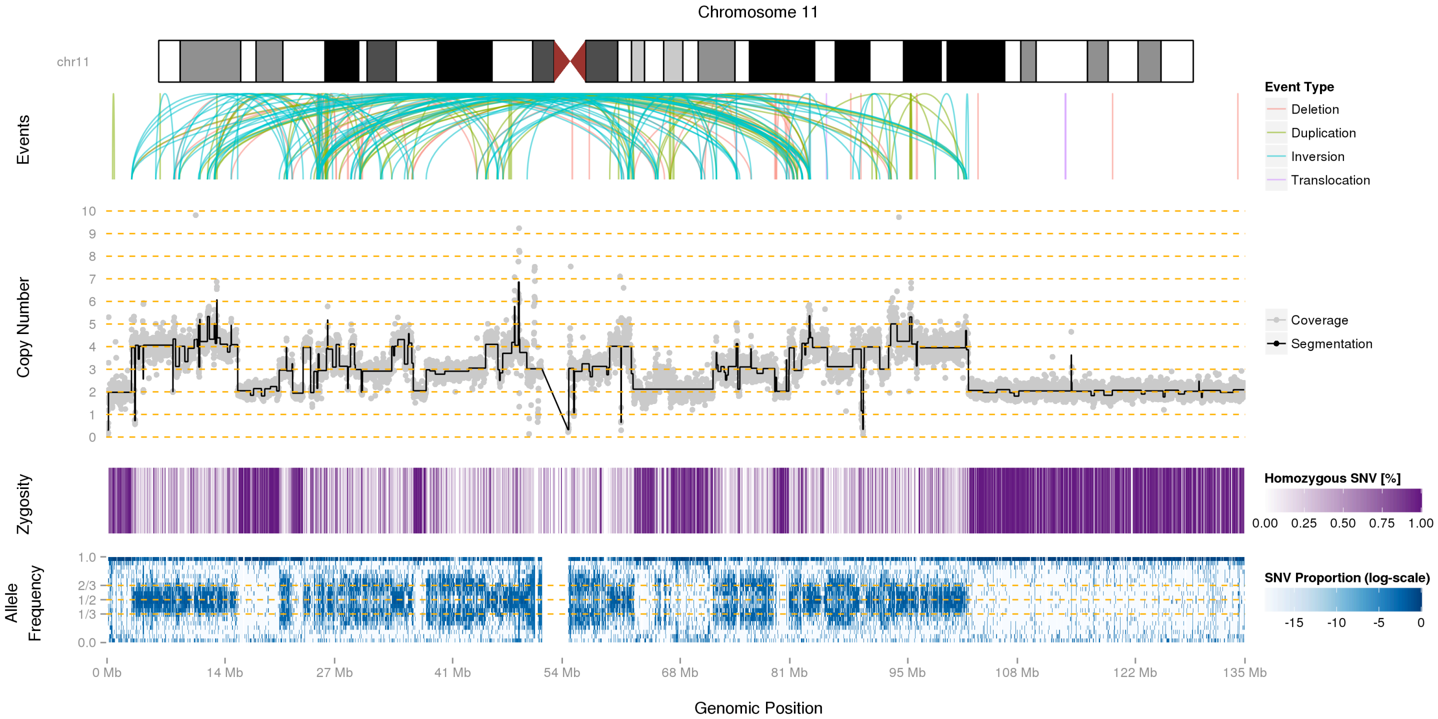
Chromosome 11
chr11
Events
Event Type
Deletion
Duplication
Inversion
Translocation
Copy Number
10
9
8
7
6
5
4
3
2
1
0
Coverage
Segmentation
Zygosity
Homozygous SNV [%]
0.00 0.25 0.50 0.75 1.00
Allele Frequency
1.0
2/3
1/2
1/3
0.0
SNV Proportion (log-scale)
-15 -10 -5 0
0 Mb 14 Mb 27 Mb 41 Mb 54 Mb 68 Mb 81 Mb 95 Mb 108 Mb 122 Mb 135 Mb
Genomic Position

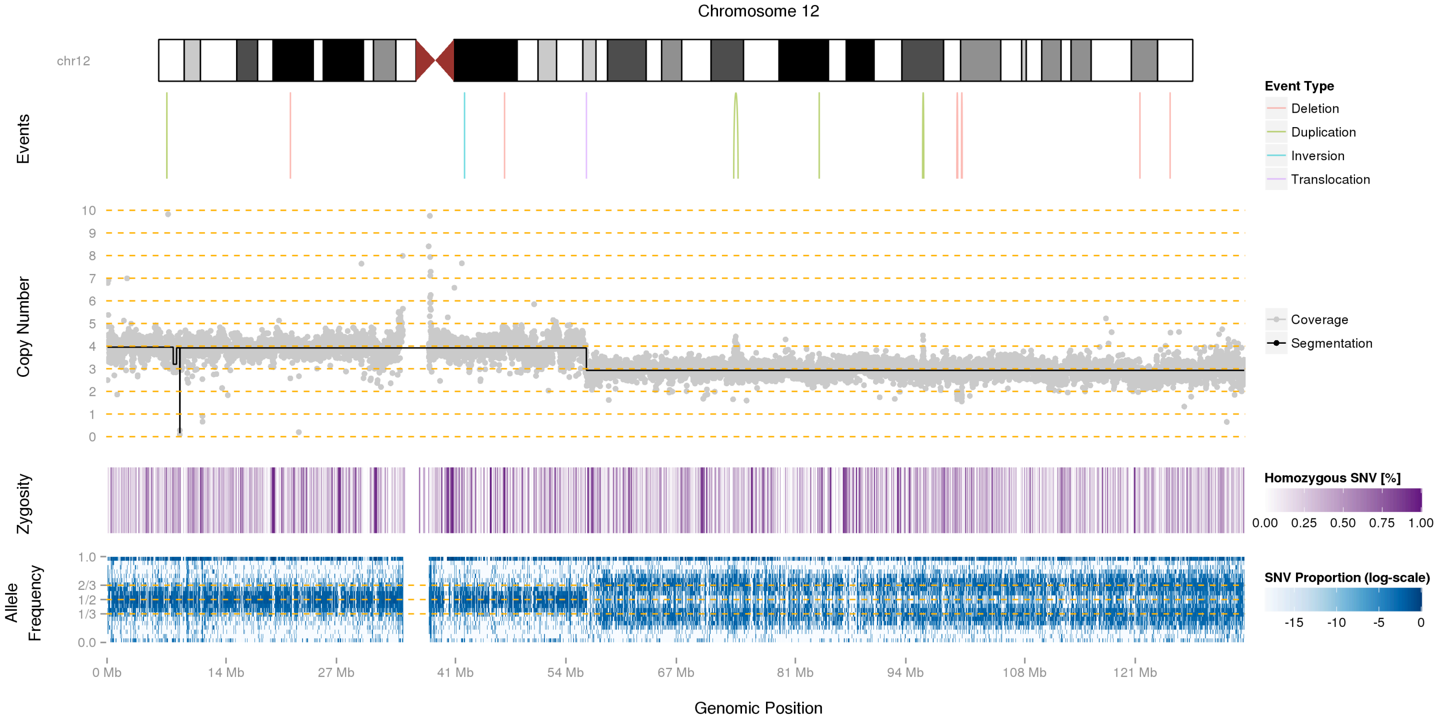
Chromosome 12
chr12
Events
Event Type
Deletion
Duplication
Inversion
Translocation
Copy Number
10
9
8
7
6
5
4
3
2
1
0
Coverage
Segmentation
Zygosity
Homozygous SNV [%]
0.00 0.25 0.50 0.75 1.00
Allele Frequency
1.0
2/3
1/2
1/3
0.0
SNV Proportion (log-scale)
-15 -10 -5 0
0 Mb 14 Mb 27 Mb 41 Mb 54 Mb 67 Mb 81 Mb 94 Mb 108 Mb 121 Mb
Genomic Position

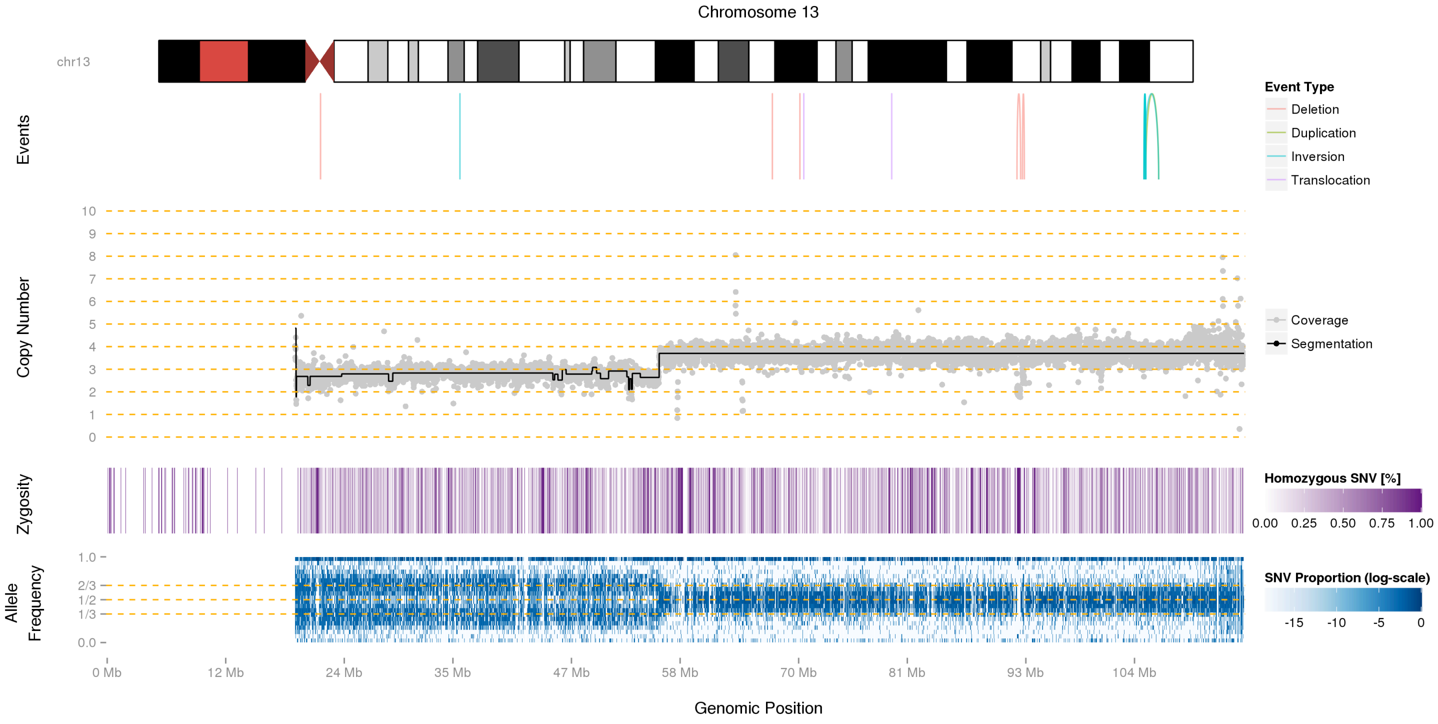
Chromosome 13
chr13
Events
Event Type
Deletion
Duplication
Inversion
Translocation
Copy Number
Coverage
Segmentation
Zygosity
Homozygous SNV [%]
0.00 0.25 0.50 0.75 1.00
Allele Frequency
SNV Proportion (log-scale)
-15 -10 -5 0
0 Mb 12 Mb 24 Mb 35 Mb 47 Mb 58 Mb 70 Mb 81 Mb 93 Mb 104 Mb
Genomic Position

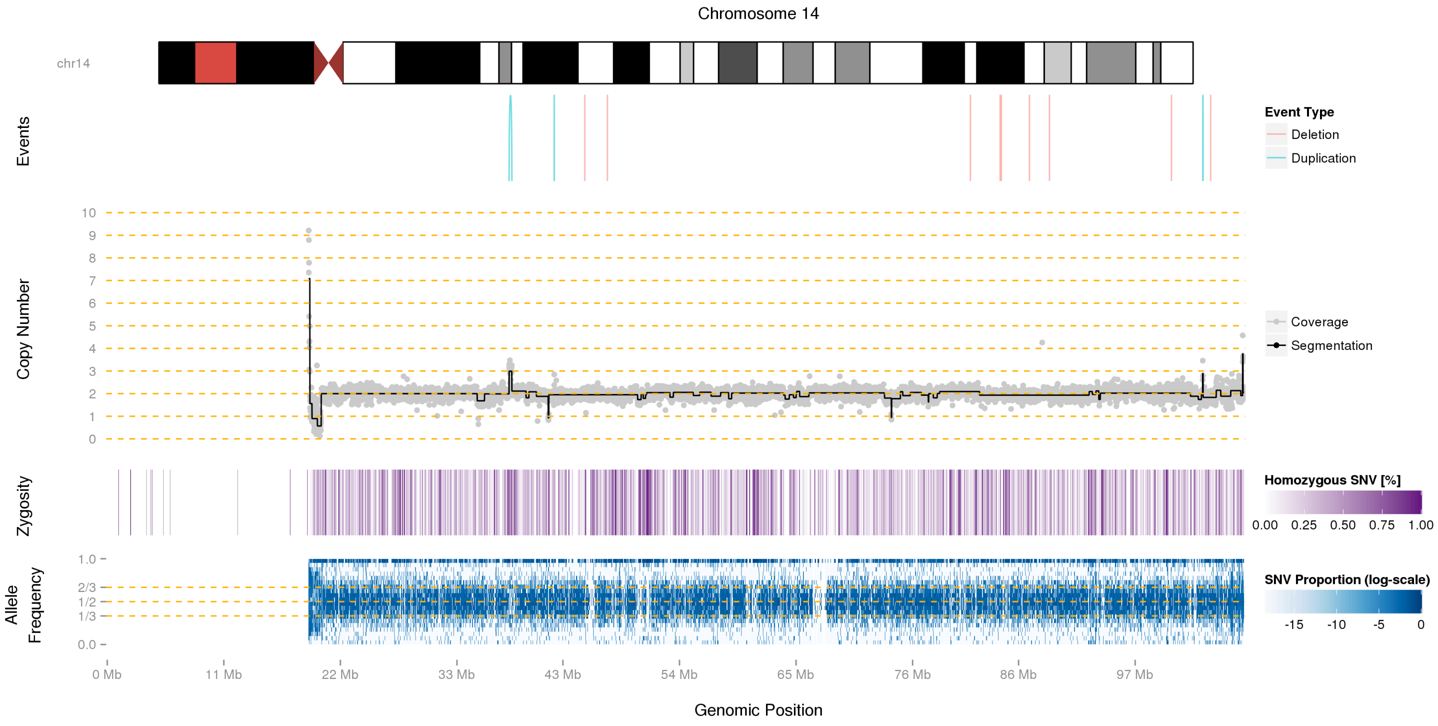
Chromosome 14
chr14
Events
Event Type
Deletion
Duplication
Copy Number
Coverage
Segmentation
Zygosity
Homozygous SNV [%]
0.00 0.25 0.50 0.75 1.00
Allele Frequency
SNV Proportion (log-scale)
-15 -10 -5 0
0 Mb 11 Mb 22 Mb 33 Mb 43 Mb 54 Mb 65 Mb 76 Mb 86 Mb 97 Mb
Genomic Position

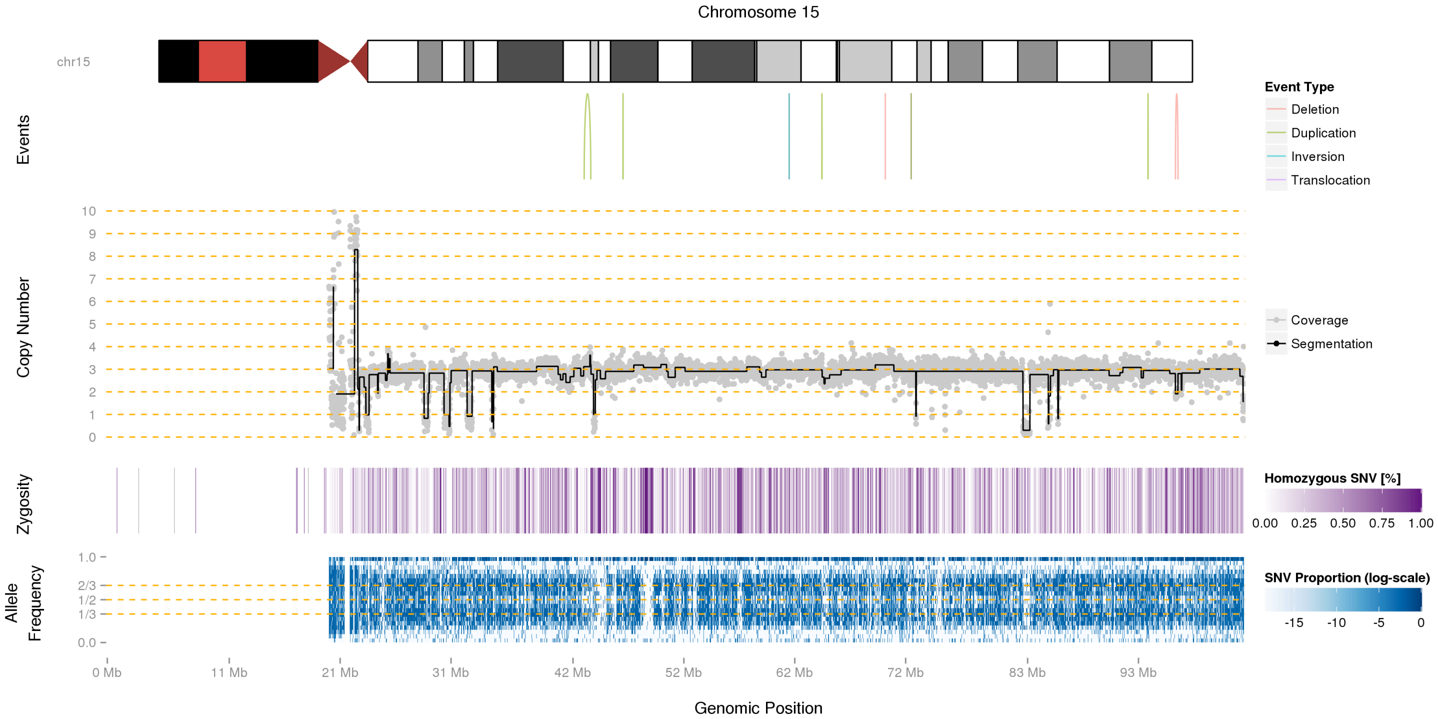
Chromosome 15
chr15
Events
Copy Number
Zygosity
Allele Frequency
Event Type
Deletion
Duplication
Inversion
Translocation
Coverage
Segmentation
Homozygous SNV [%]
0.00 0.25 0.50 0.75 1.00
SNV Proportion (log-scale)
-15 -10 -5 0
0 Mb 11 Mb 21 Mb 31 Mb 42 Mb 52 Mb 62 Mb 72 Mb 83 Mb 93 Mb
Genomic Position

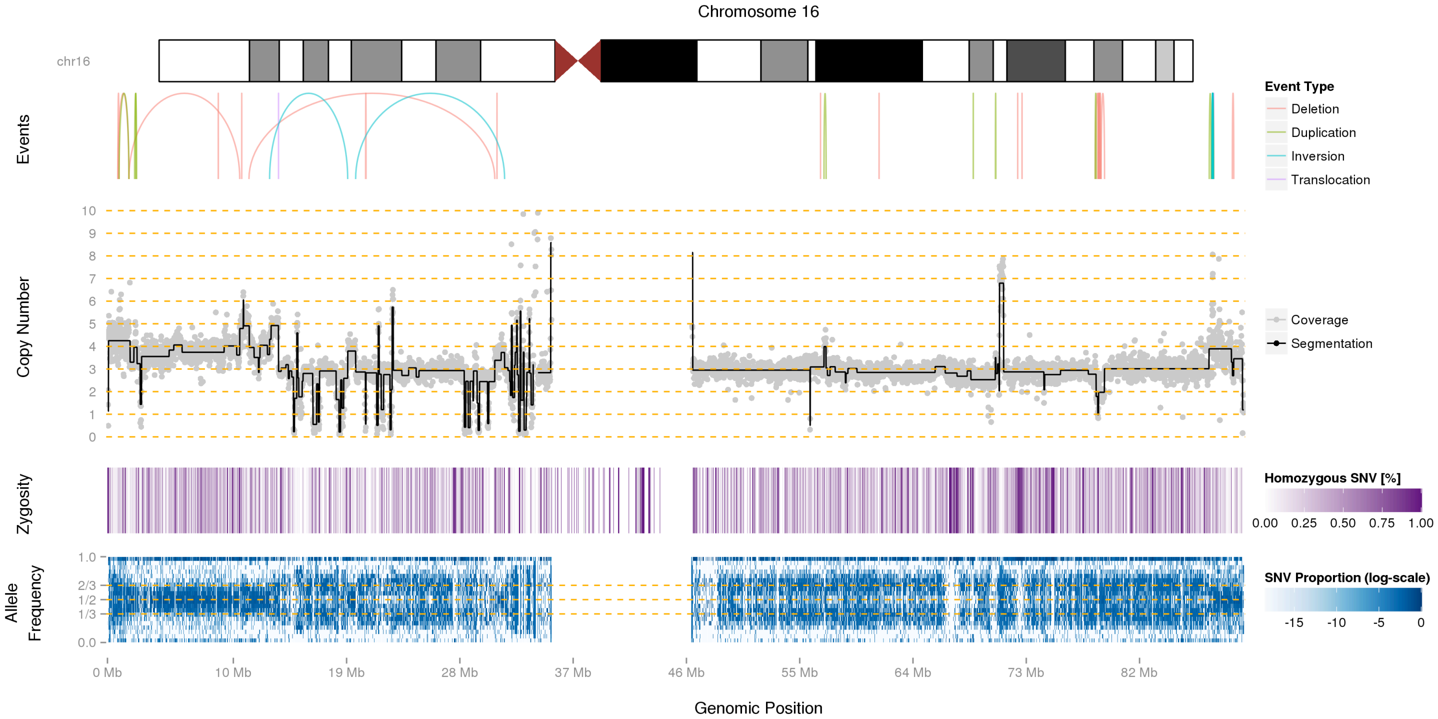
Chromosome 16
chr16
Events
Copy Number
Zygosity
Allele Frequency
Event Type
Deletion
Duplication
Inversion
Translocation
Coverage
Segmentation
Homozygous SNV [%]
0.00 0.25 0.50 0.75 1.00
SNV Proportion (log-scale)
-15 -10 -5 0
0 Mb 10 Mb 19 Mb 28 Mb 37 Mb 46 Mb 55 Mb 64 Mb 73 Mb 82 Mb
Genomic Position

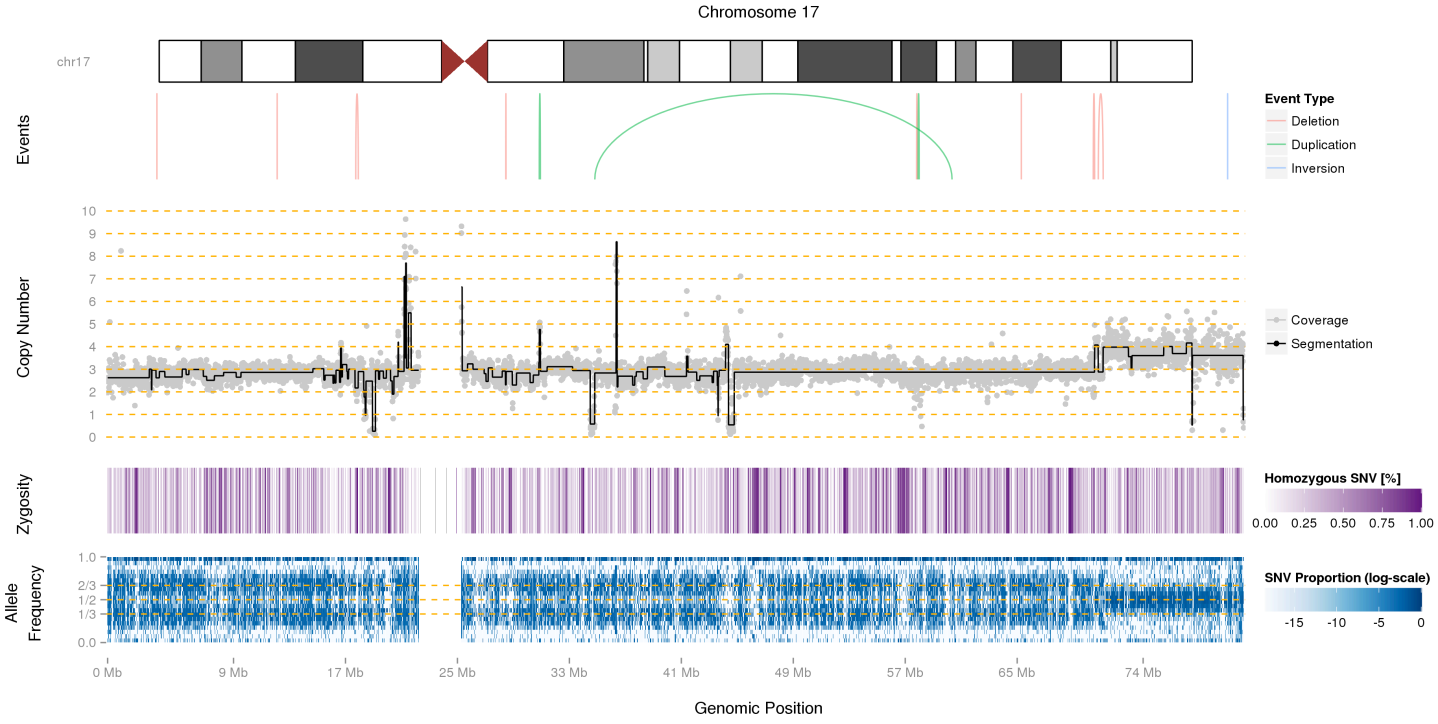
Chromosome 17
chr17
Events
Event Type
Deletion
Duplication
Inversion
Copy Number
10
9
8
7
6
5
4
3
2
1
0
Coverage
Segmentation
Zygosity
Homozygous SNV [%]
0.00 0.25 0.50 0.75 1.00
Allele Frequency
1.0
2/3
1/2
1/3
0.0
SNV Proportion (log-scale)
-15 -10 -5 0
0 Mb 9 Mb 17 Mb 25 Mb 33 Mb 41 Mb 49 Mb 57 Mb 65 Mb 74 Mb
Genomic Position

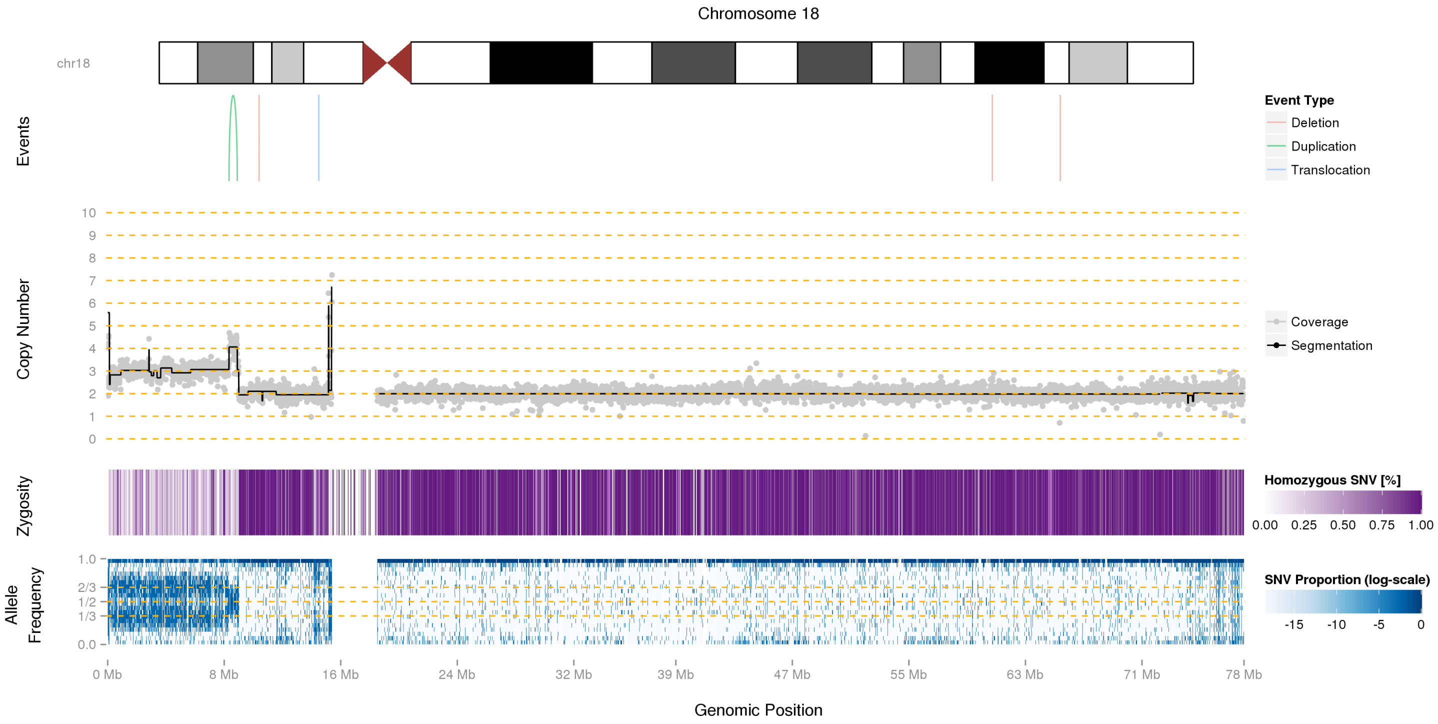
Chromosome 18
chr18
Events
Event Type
Deletion
Duplication
Translocation
Copy Number
10
9
8
7
6
5
4
3
2
1
0
Coverage
Segmentation
Zygosity
Homozygous SNV [%]
0.00 0.25 0.50 0.75 1.00
Allele Frequency
1.0
2/3
1/2
1/3
0.0
SNV Proportion (log-scale)
-15 -10 -5 0
0 Mb 8 Mb 16 Mb 24 Mb 32 Mb 39 Mb 47 Mb 55 Mb 63 Mb 71 Mb 78 Mb
Genomic Position

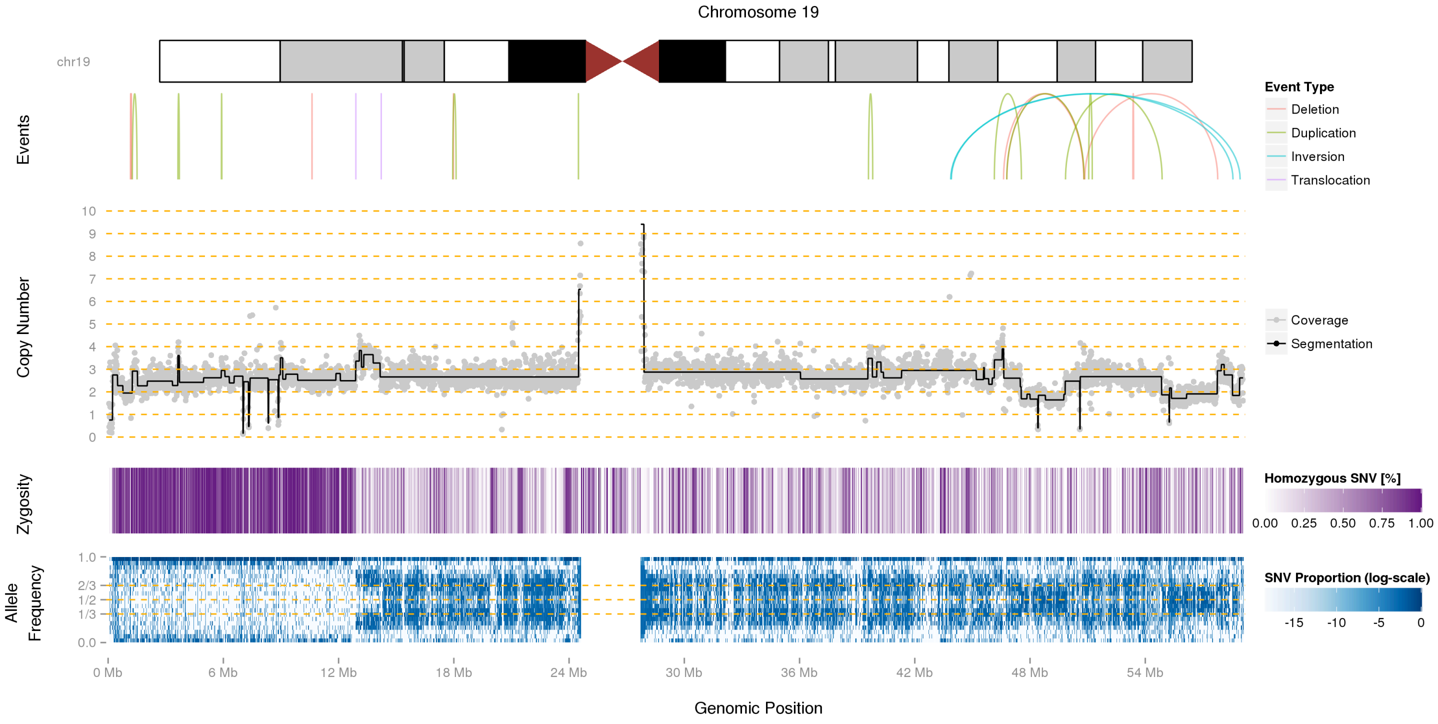
Chromosome 19
chr19
Events
Event Type
Deletion
Duplication
Inversion
Translocation
Copy Number
10
9
8
7
6
5
4
3
2
1
0
Coverage
Segmentation
Zygosity
Homozygous SNV [%]
0.00
0.25
0.50
0.75
1.00
Allele Frequency
1.0
2/3
1/2
1/3
0.0
SNV Proportion (log-scale)
-15
-10
-5
0
0 Mb
6 Mb
12 Mb
18 Mb
24 Mb
30 Mb
36 Mb
42 Mb
48 Mb
54 Mb
Genomic Position

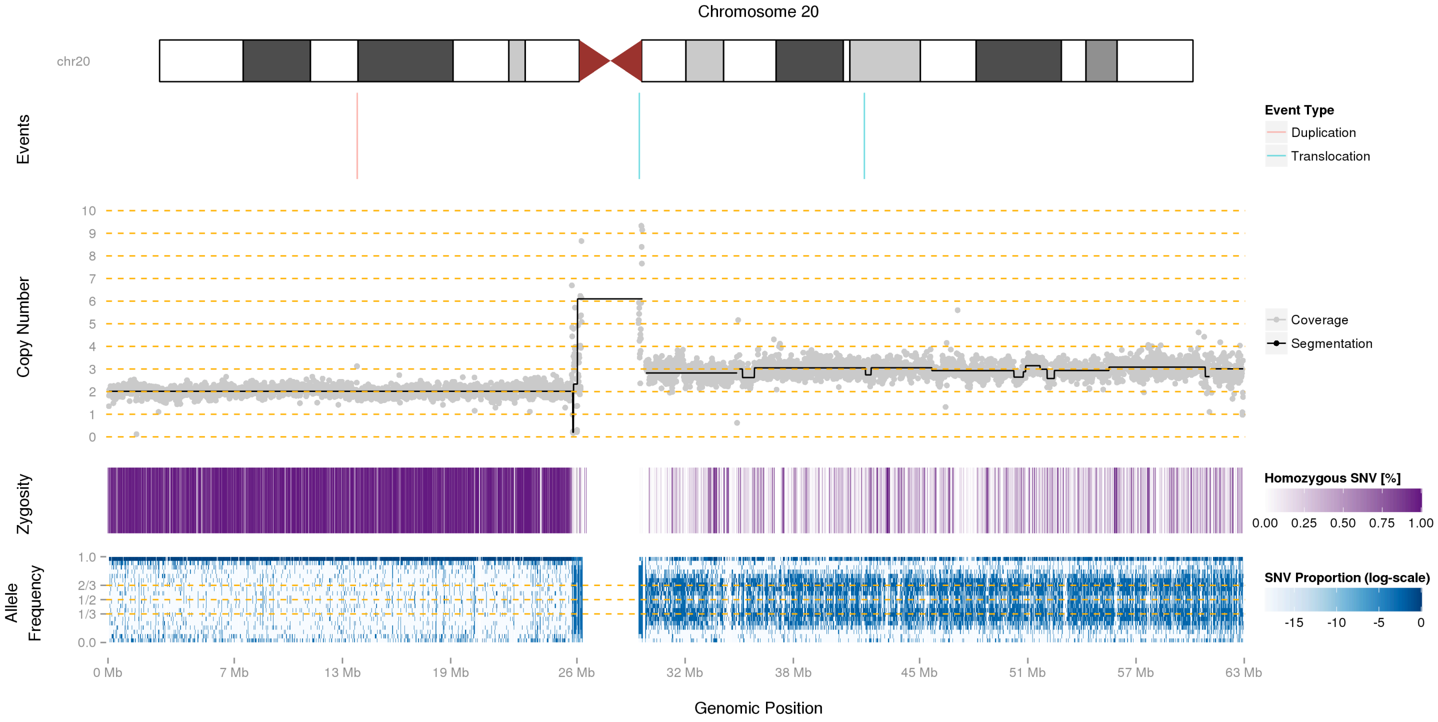
Chromosome 20
chr20
Events
Event Type
Duplication
Translocation
Copy Number
10
9
8
7
6
5
4
3
2
1
0
Coverage
Segmentation
Zygosity
Homozygous SNV [%]
0.00
0.25
0.50
0.75
1.00
Allele Frequency
1.0
2/3
1/2
1/3
0.0
SNV Proportion (log-scale)
-15
-10
-5
0
0 Mb
7 Mb
13 Mb
19 Mb
26 Mb
32 Mb
38 Mb
45 Mb
51 Mb
57 Mb
63 Mb
Genomic Position

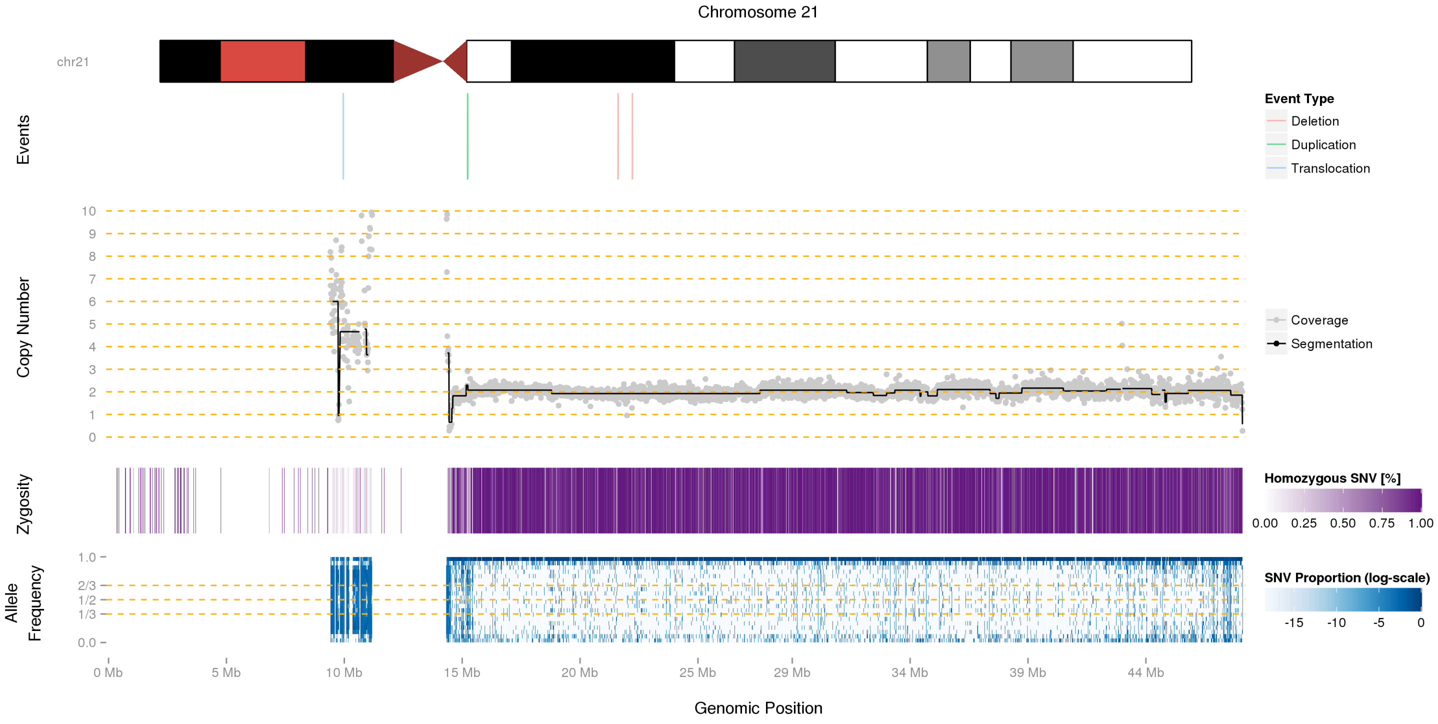
Chromosome 21
chr21
Events
Event Type
Deletion
Duplication
Translocation
Copy Number
Coverage
Segmentation
Zygosity
Homozygous SNV [%]
0.00 0.25 0.50 0.75 1.00
Allele Frequency
SNV Proportion (log-scale)
-15 -10 -5 0
0 Mb 5 Mb 10 Mb 15 Mb 20 Mb 25 Mb 29 Mb 34 Mb 39 Mb 44 Mb
Genomic Position

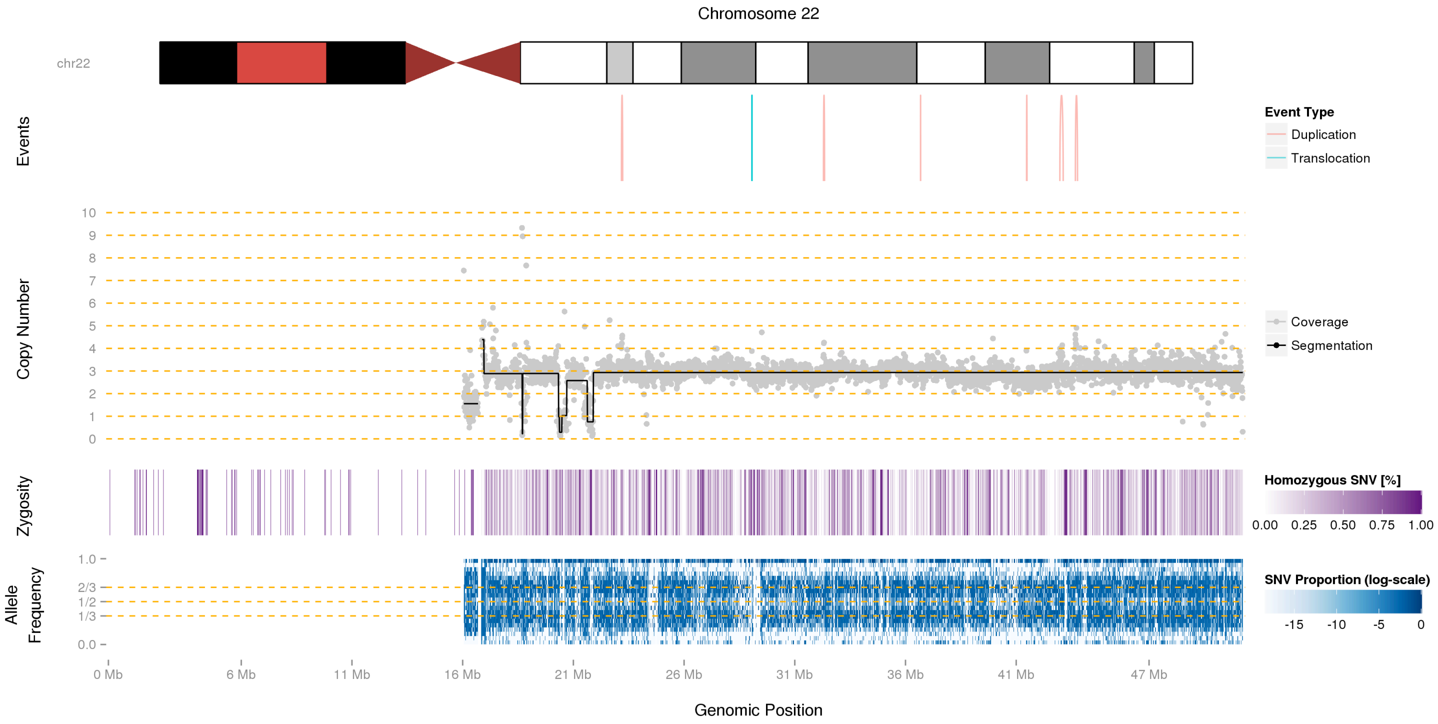
Chromosome 22
chr22
Events
Event Type
Duplication
Translocation
Copy Number
Coverage
Segmentation
Zygosity
Homozygous SNV [%]
0.00 0.25 0.50 0.75 1.00
Allele Frequency
SNV Proportion (log-scale)
-15 -10 -5 0
0 Mb 6 Mb 11 Mb 16 Mb 21 Mb 26 Mb 31 Mb 36 Mb 41 Mb 47 Mb
Genomic Position

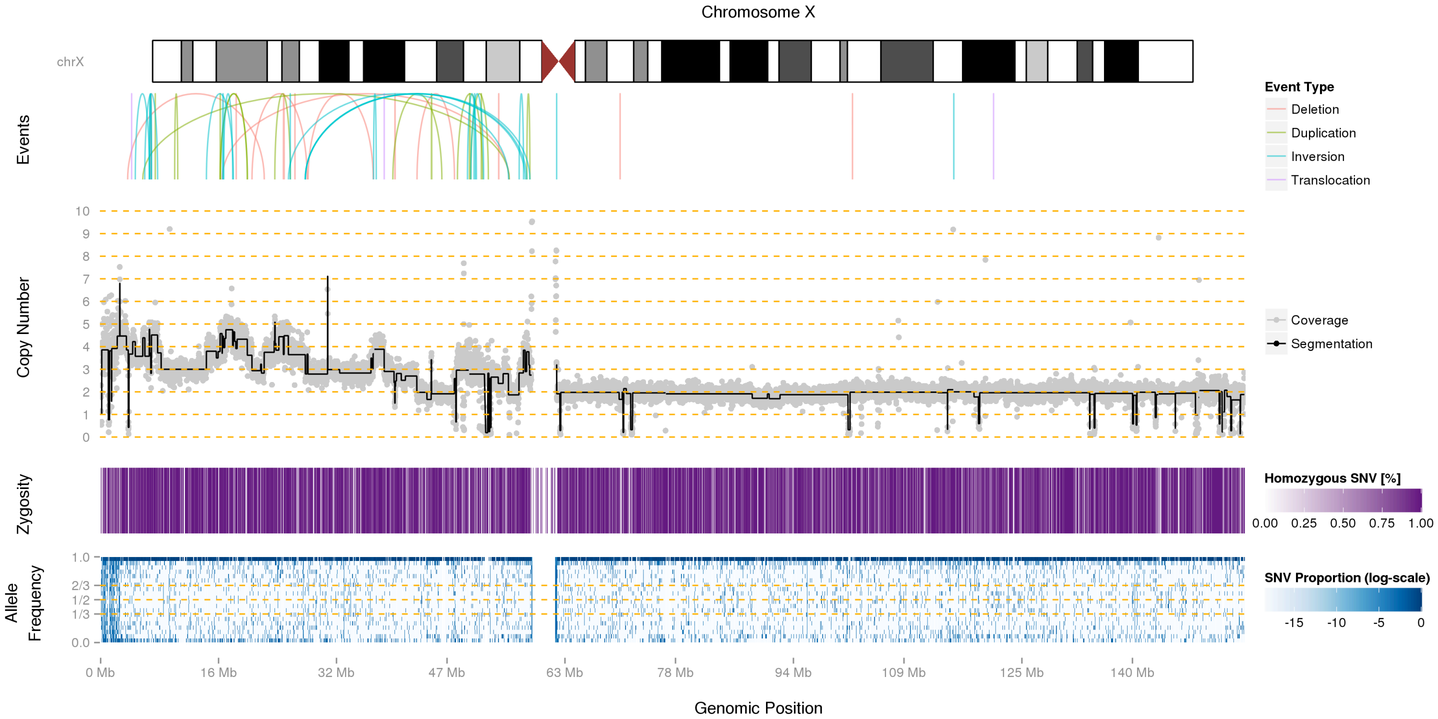

Figure S2 Structural variants, copy number and loss of heterozygosity for chromosomes 1 to 22 and X.
Arcs in the top panels labeled 'Events' represent the predicted connections between fragments derived from SV calls based on read pair orientation and spacing. Different read pair signatures indicate the following event types: deletions, tandem duplications, inversions, and interchromosomal translocations. The center panel (Copy Number) represents the copy number estimates in 10 kb bins (grey) overlaid with their segmentation (black). The associated CN is shown on the y-axis. The zygosity track shows the proportion of homozygous SNV calls in 10 kb bins, darker purple regions contain more homozygous calls (up to 100%) and indicate potential LoH. The bottom panel shows the allele frequency (AFS) distribution as a heatmap in 10 kb bins on the chromosome axis and 5% bins on the allele frequency axis; darker blue indicates more SNVs with the given AF in the corresponding 10 kb region. The color scale is according to the log of proportion of SNVs falling into the AFS bin (e.g. 10-15%, i.e. the row) in the 10 kb region (i.e. the column).

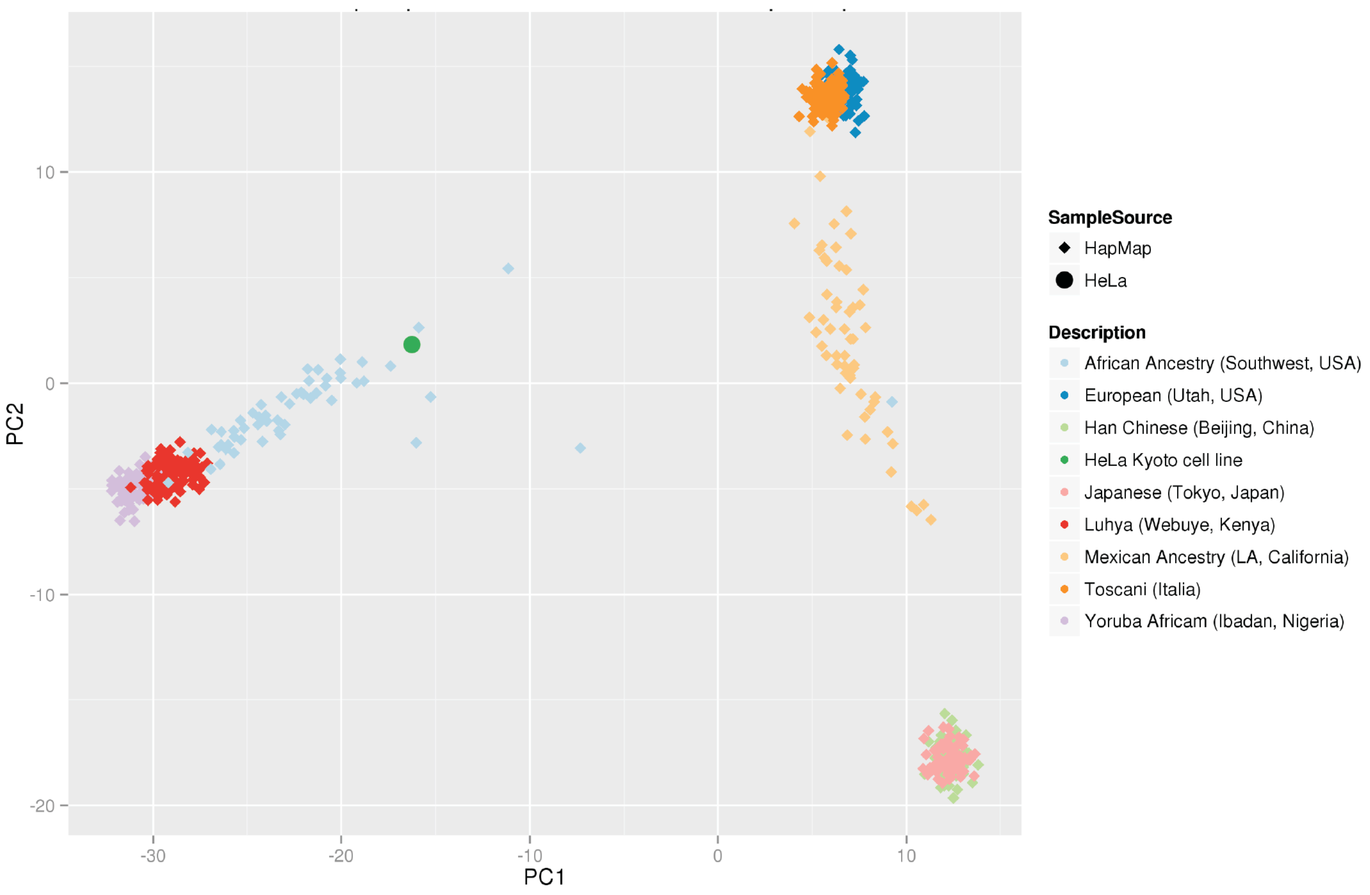

Figure S3 Principal component analysis of SNVs in HeLa Kyoto and 640 HapMap individuals from 8 different populations.
The populations are:
CEU - Northern and Western European ancestry from Utah, USA;
CHB - Han Chinese from Beijing, China;
JPT - Japanese from Tokyo, Japan;
YRI - Yoruba from Ibadan, Nigeria;
ASW - African ancestry from Southwest USA;
LWK - Luhya from Webuye, Kenya;
MXL - Mexican ancestry from Los Angeles, USA;
TSI - Toscani from Italy.

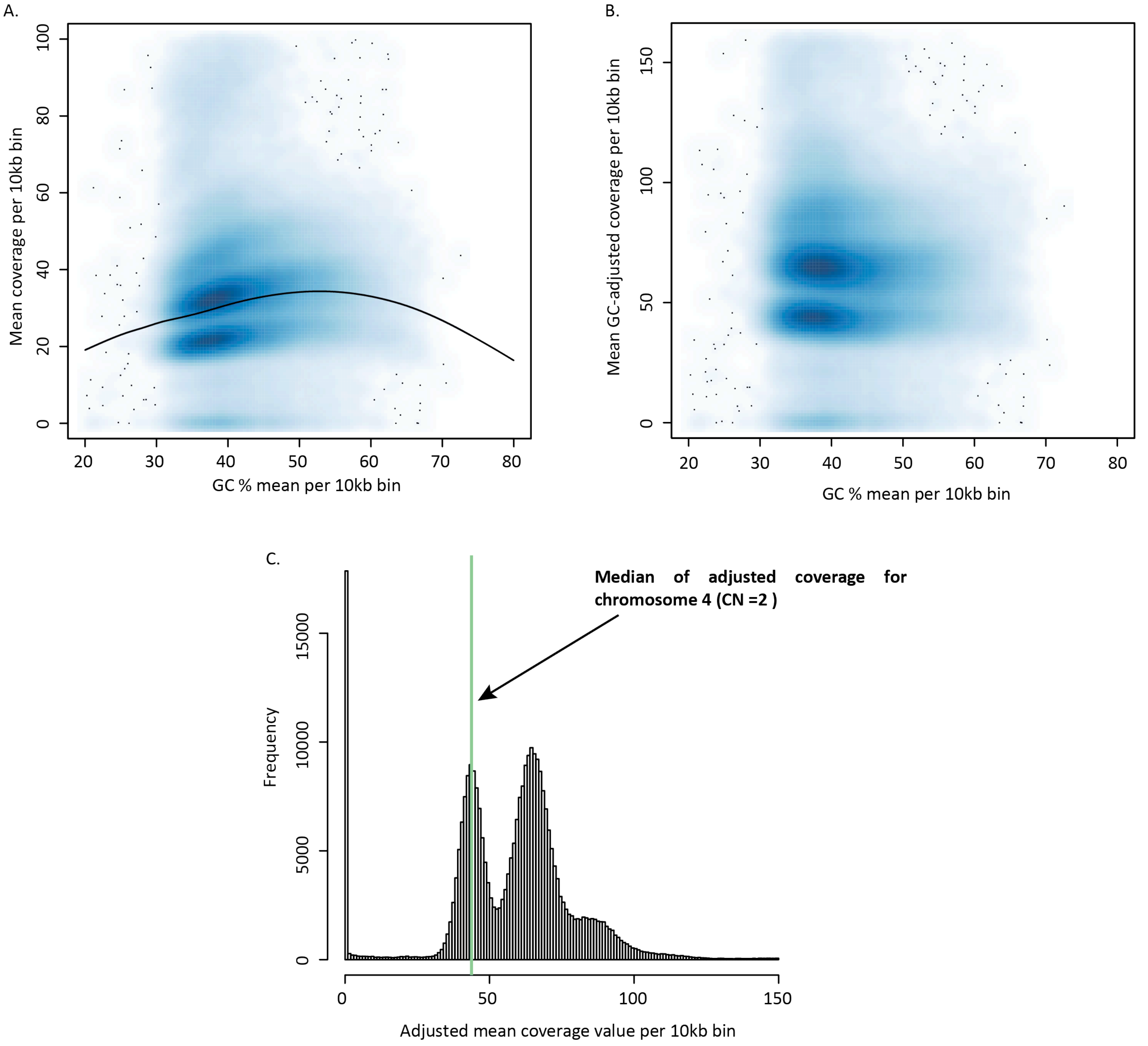

Figure S4 Effect of GC adjustment on sequencing coverage.

(A) Mean coverage value per 10 kb bin (y-axis) as a function of the mean GC % per 10 kb bin, before any adjustment was applied. The black line represents a robust local regression fitted on the data.

(B) Adjusted mean coverage value per 10 kb bin (y-axis) as a function of the mean GC % per 10 kb bin.

(C) Distribution of adjusted mean coverage value per 10 kb bins along the genome. The green line corresponds to the adjusted coverage median value for chromosome 4, which was used for normalization as representative of regions with CN 2.

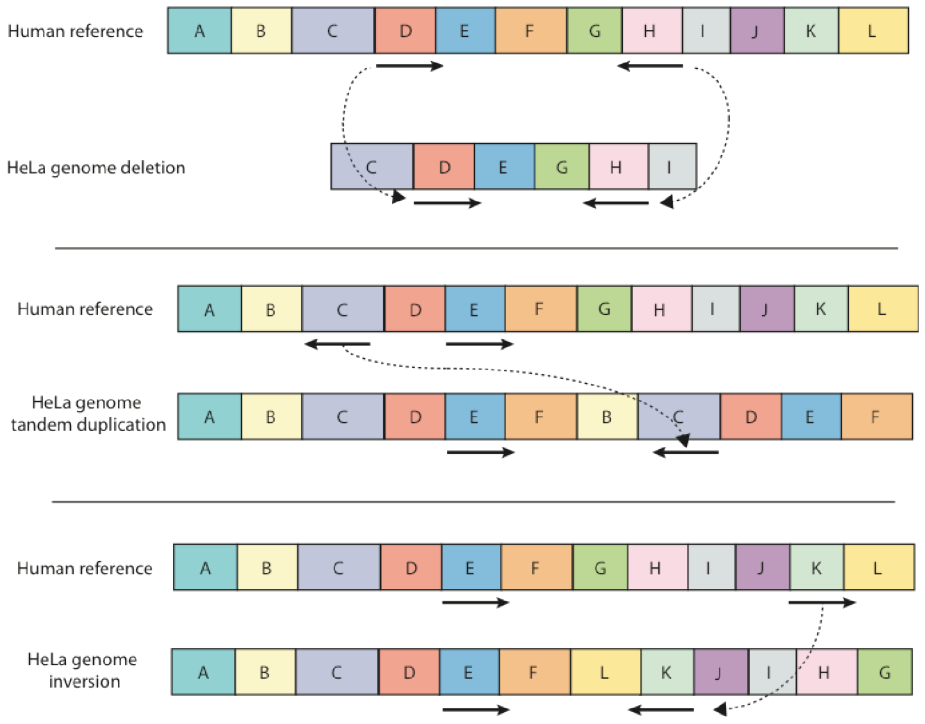

Figure S5 Primer design for detection of genomic rearrangements.
For deletions, the amplicons generated are smaller than expected size. The orientation of the primer targeting tandem duplication and inversion allow detection of an amplicon only if the event is present.

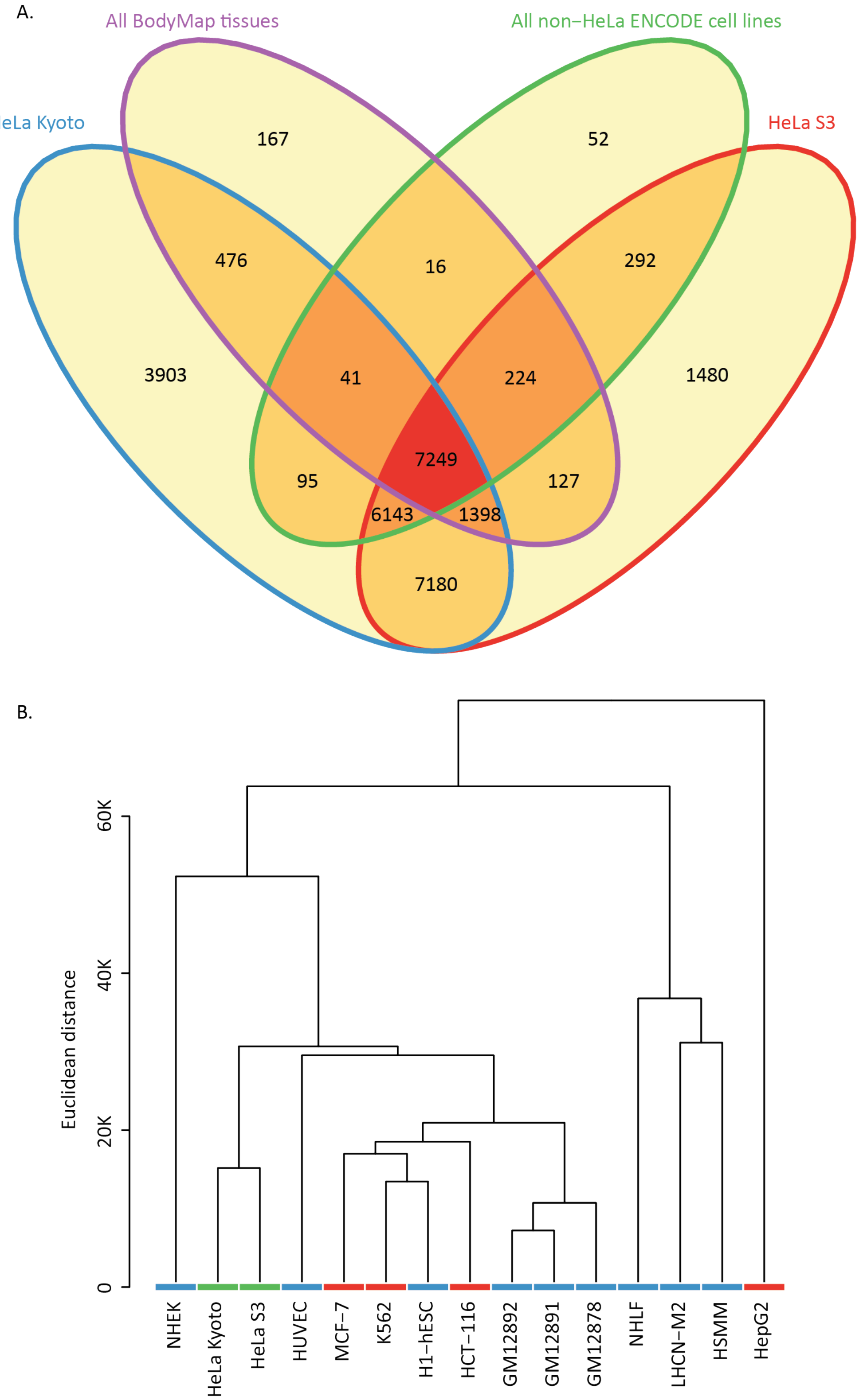

Figure S6 Comparison of HeLa transcriptome profile to Illumina Body Map tissues and ENCODE cell lines.
(A) Venn diagram of genes with nondetectable expression across all ENCODE cell lines (excluding HeLa-S3), all Body Map tissues, HeLa Kyoto and HeLa S3. The numbers indicate genes (listed in the human reference annotation file from ENSEMBL, see Methods).
(B) Dendrogram comparing the transcription profiles of all ENCODE cell lines and the HeLa Kyoto cell line. The y-axis is the Euclidean distance between cell lines.

Table S1   Potential viral insertions

Chr	Start	End	Strand	Read support	Viral species
1	10002	10118	+	12	Human herpesvirus 6B<br>Equid herpesvirus 2<br>Human herpesvirus 6A<br>Human herpesvirus 7<br>Gallid herpesvirus 2<br>Gallid herpesvirus 3<br>Meleagrid herpesvirus 1<br>Ovine herpesvirus 2<br>Cyprinid herpesvirus 3<br>Saimiriine herpesvirus 1
8	128189764	128190032	-	552	Human papillomavirus 18 and 32<br>(Alphapapillomavirus 7 and 1)
8	128192272	128192499	+	23	Human papillomavirus 18<br>(Alphapapillomavirus 7)
8	128193167	128193435	+	174	
8	128200419	128200690	+	163	
12	49659073	49659136	-	6	Human herpesvirus 5
12	95467	95567	+	9	Human herpesvirus 6B<br>Human herpesvirus 6A<br>Human herpesvirus 7<br>Gallid herpesvirus 2<br>Gallid herpesvirus 3<br>Meleagrid herpesvirus 1<br>Cyprinid herpesvirus 3
13	19648667	19648780	+	7	Taterapox virus
13	19649043	19649209	-	8	
X	155185203	155185362	-	8	Human herpesvirus 6B<br>Equid herpesvirus 2<br>Human herpesvirus 6A<br>Human herpesvirus 7<br>Gallid herpesvirus 2<br>Gallid herpesvirus 3<br>Meleagrid herpesvirus 1<br>Ovine herpesvirus 2
