# Supplementary material for: The Genomic and Transcriptomic Landscape of a HeLa Cell Line
Source: G3 (Bethesda). 2013 Mar 11;3(8):1213–24. doi: 10.1534/g3.113.005777 (PMC3737162; doi:10.1534/g3.113.005777)
Supplement: Supporting Information [file supp_g3.113.005777_FigureS1.pdf]

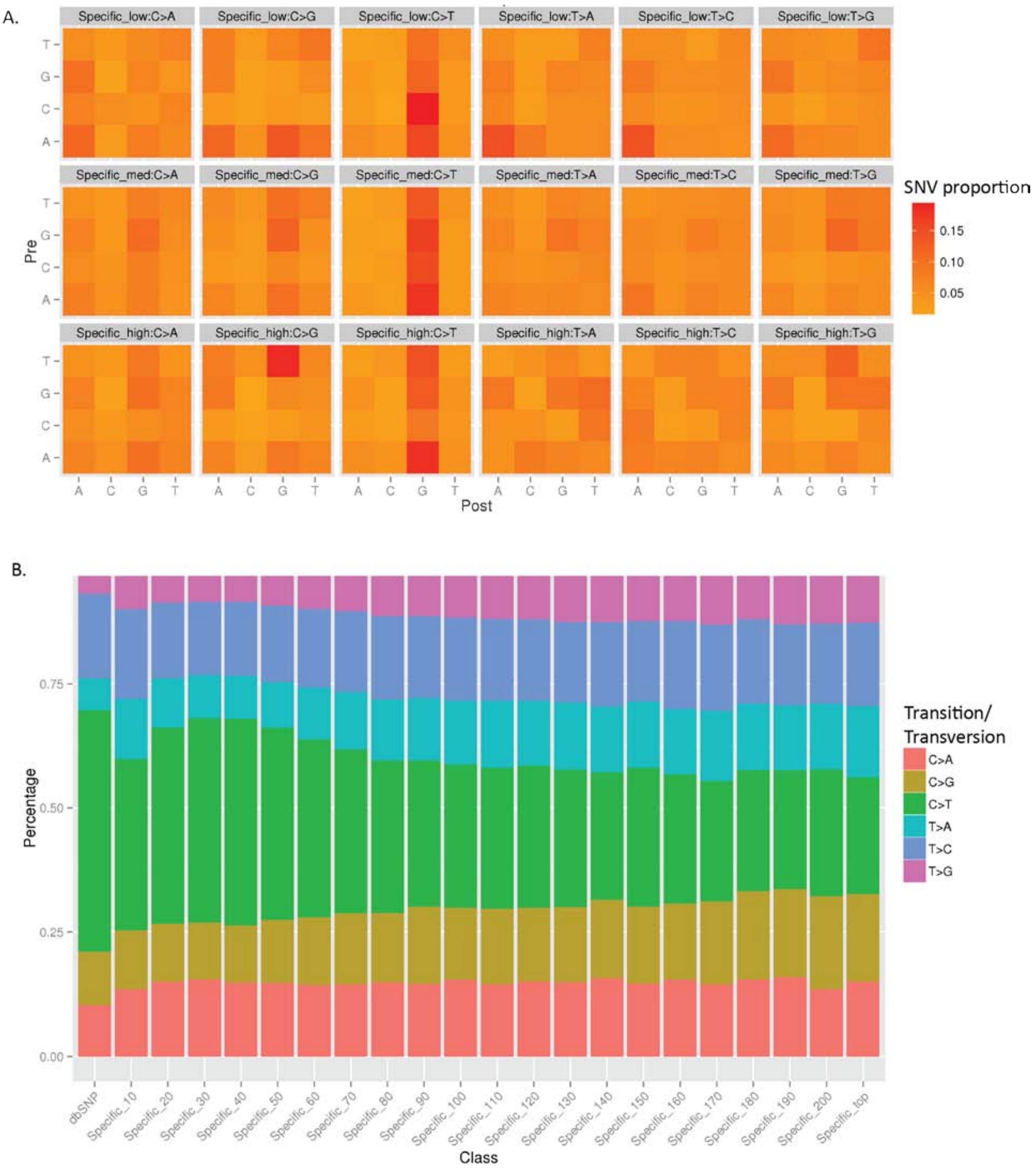

Figure S1 Mutational spectra in the HeLa Kyoto genome.

(A) Heatmap representation of mutational spectra observed in HeLa Kyoto specific calls stratified by local coverage (low: < 10; med: >=10 and < 60; high: >= 60). Each row corresponds to one group of SNVs and each column to a type of mutation (e.g.: C>A). Each heatmap shows the observed proportion of SNVs for all possible combinations of preceding ('Pre') and following ('Post') bases.

(B) Stacked barplots of the distribution of mutation types (e.g.: C>T) in observed HeLa specific SNVs, stratified by local coverage: Column "Specific_x" represents calls with coverage between x-10 and x. Column "dbSNP" shows called SNVs that have dbSNP ids, column "Specific_top" shows calls with local coverage greater than 200.
