# Supplementary material for: The Genomic and Transcriptomic Landscape of a HeLa Cell Line
Source: G3 (Bethesda). 2013 Mar 11;3(8):1213–24. doi: 10.1534/g3.113.005777 (PMC3737162; doi:10.1534/g3.113.005777)
Supplement: Supporting Information [file supp_g3.113.005777_FigureS2.pdf]

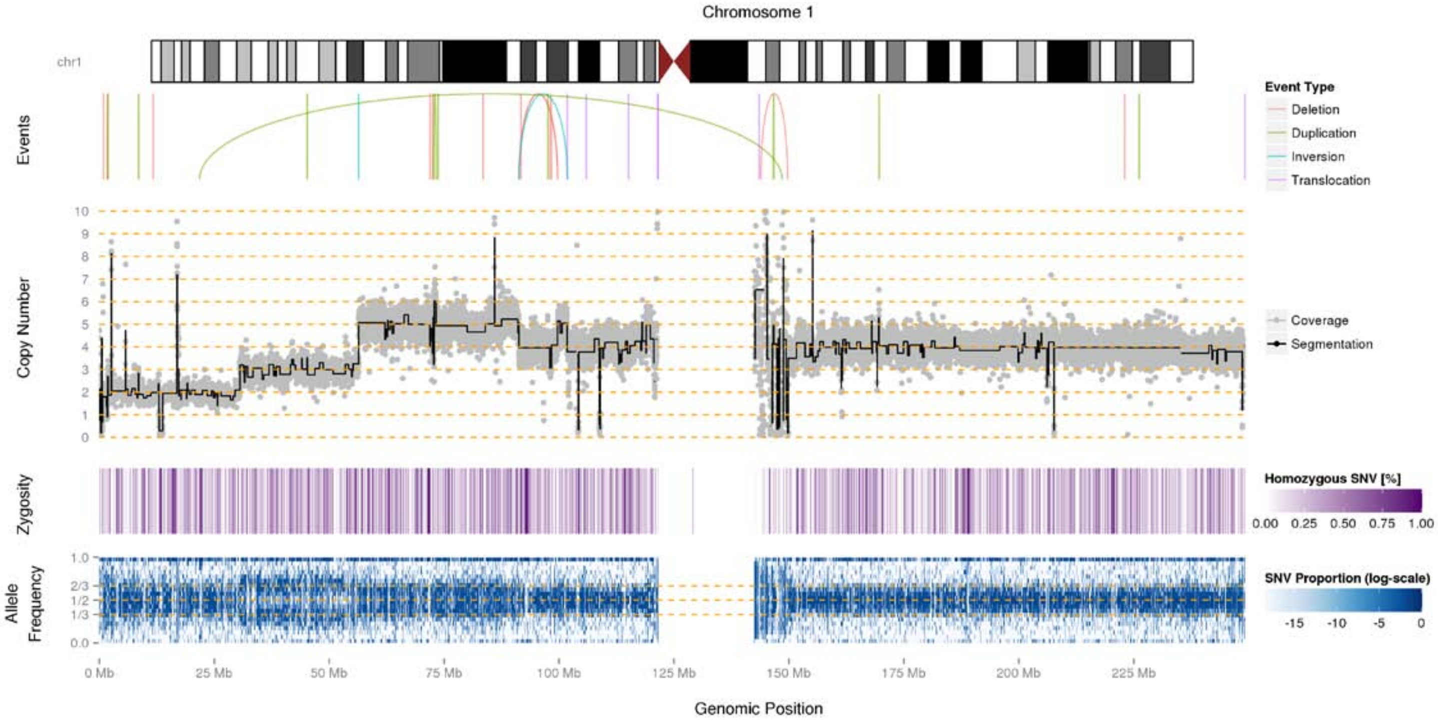
Chromosome 1
chr1
Events
Copy Number
Zygosity
Allele Frequency
Event Type
Deletion
Duplication
Inversion
Translocation
Coverage
Segmentation
Homozygous SNV [%]
0.00 0.25 0.50 0.75 1.00
SNV Proportion (log-scale)
-15 -10 -5 0
0 Mb 25 Mb 50 Mb 75 Mb 100 Mb 125 Mb 150 Mb 175 Mb 200 Mb 225 Mb
Genomic Position

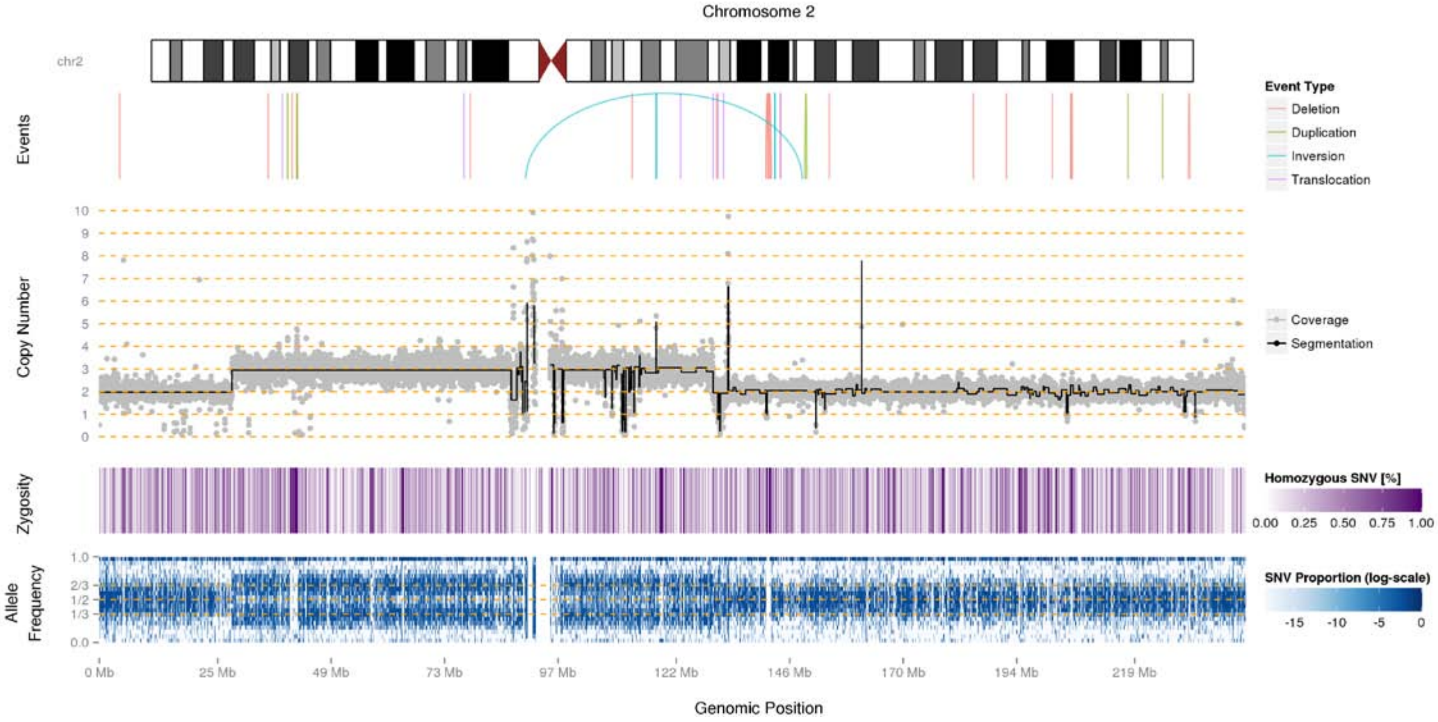
Chromosome 2
chr2
Events
Copy Number
Zygosity
Allele Frequency
Event Type
Deletion
Duplication
Inversion
Translocation
Coverage
Segmentation
Homozygous SNV [%]
0.00 0.25 0.50 0.75 1.00
SNV Proportion (log-scale)
-15 -10 -5 0
0 Mb 25 Mb 49 Mb 73 Mb 97 Mb 122 Mb 146 Mb 170 Mb 194 Mb 219 Mb
Genomic Position

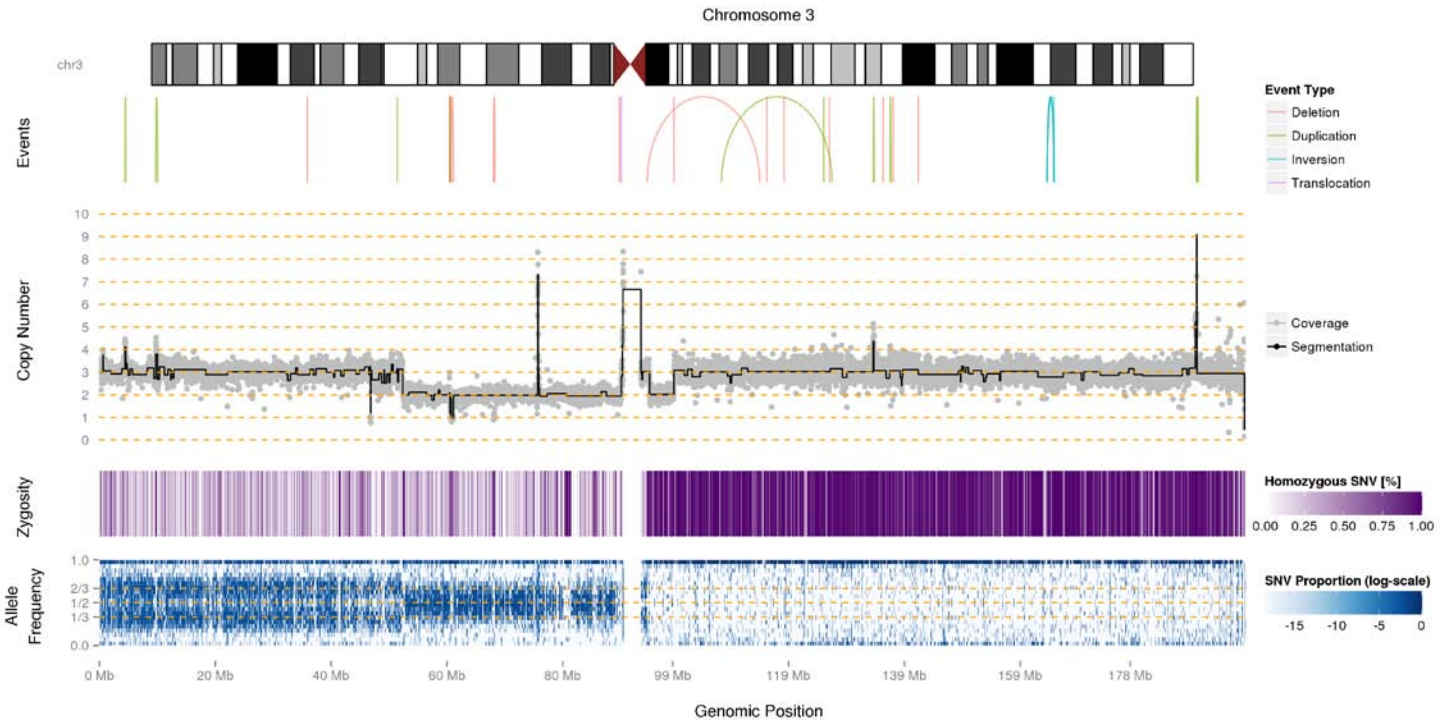
Chromosome 3
chr3
Events
Event Type
Deletion
Duplication
Inversion
Translocation
Copy Number
Coverage
Segmentation
Zygosity
Homozygous SNV [%]
0.00 0.25 0.50 0.75 1.00
Allele Frequency
SNV Proportion (log-scale)
-15 -10 -5 0
0 Mb 20 Mb 40 Mb 60 Mb 80 Mb 99 Mb 119 Mb 139 Mb 159 Mb 178 Mb
Genomic Position

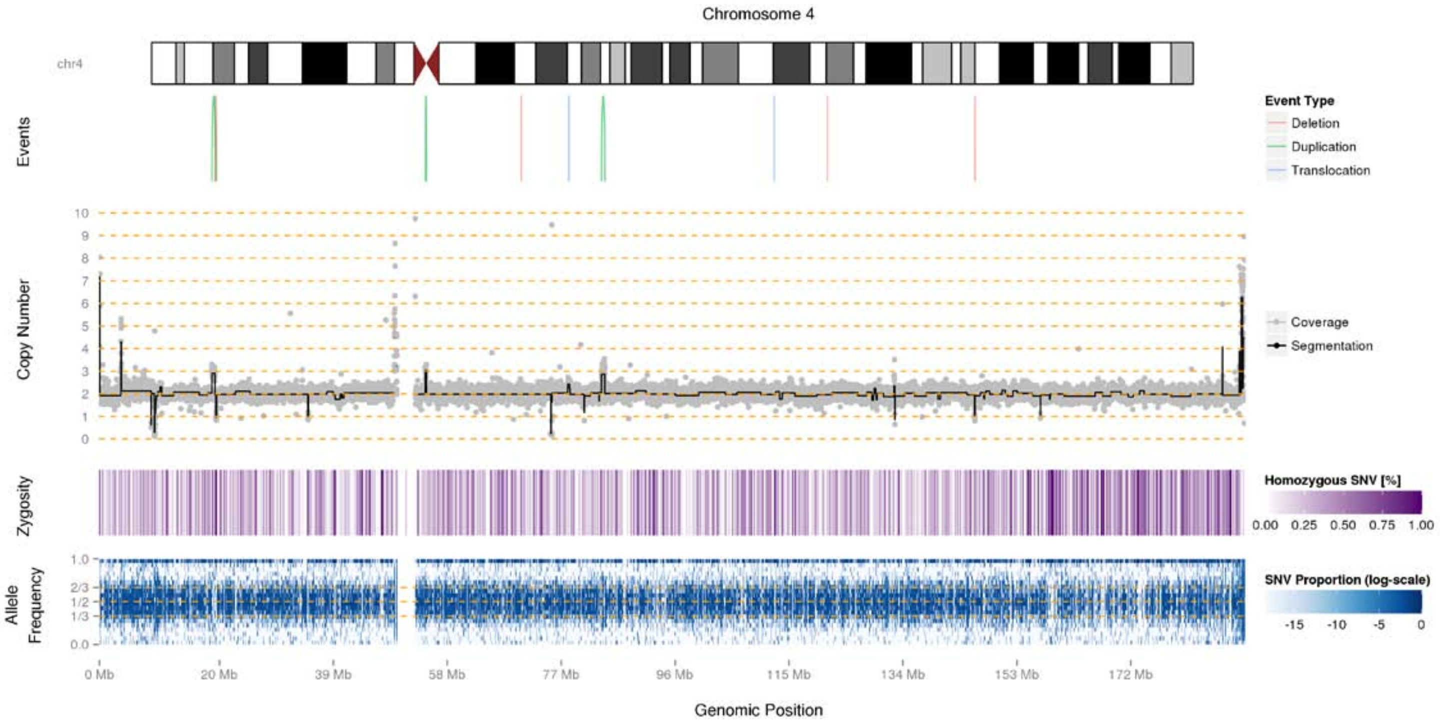
Chromosome 4
chr4
Events
Event Type
Deletion
Duplication
Translocation
Copy Number
Coverage
Segmentation
Zygosity
Homozygous SNV [%]
0.00 0.25 0.50 0.75 1.00
Allele Frequency
SNV Proportion (log-scale)
-15 -10 -5 0
0 Mb 20 Mb 39 Mb 58 Mb 77 Mb 96 Mb 115 Mb 134 Mb 153 Mb 172 Mb
Genomic Position

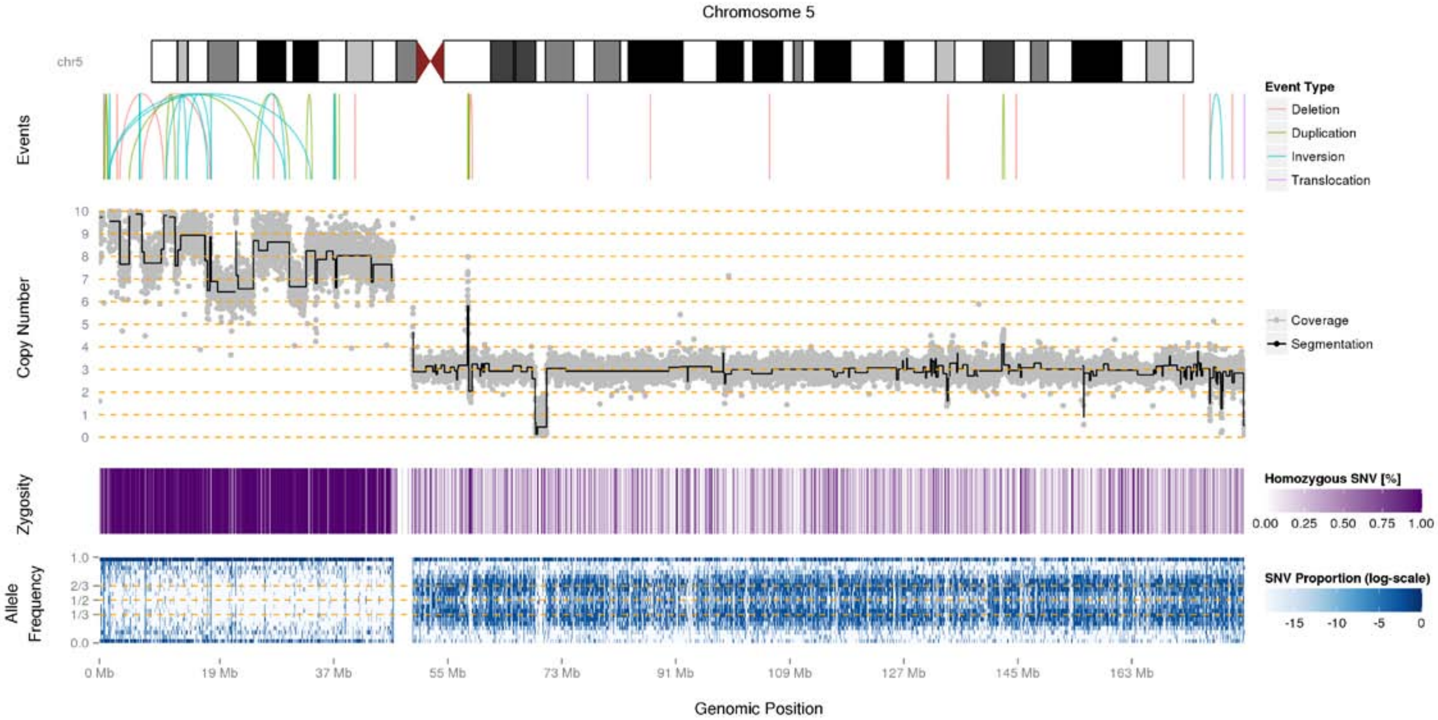
Chromosome 5
chr5
Events
Event Type
Deletion
Duplication
Inversion
Translocation
Copy Number
10
9
8
7
6
5
4
3
2
1
0
Coverage
Segmentation
Zygosity
Homozygous SNV [%]
0.00 0.25 0.50 0.75 1.00
Allele Frequency
1.0
2/3
1/2
1/3
0.0
SNV Proportion (log-scale)
-15 -10 -5 0
0 Mb
19 Mb
37 Mb
55 Mb
73 Mb
91 Mb
109 Mb
127 Mb
145 Mb
163 Mb
Genomic Position

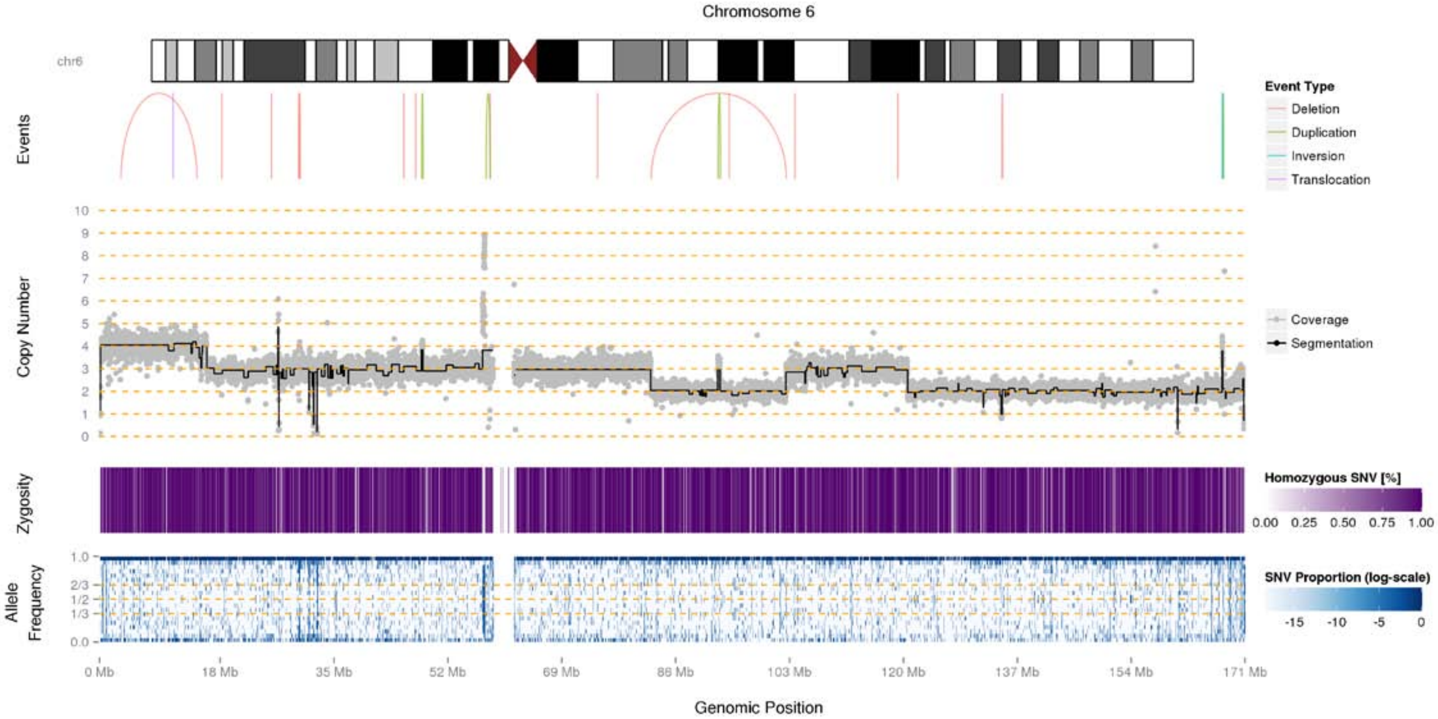
Chromosome 6
chr6
Events
Event Type
Deletion
Duplication
Inversion
Translocation
Copy Number
10
9
8
7
6
5
4
3
2
1
0
Coverage
Segmentation
Zygosity
Homozygous SNV [%]
0.00 0.25 0.50 0.75 1.00
Allele Frequency
1.0
2/3
1/2
1/3
0.0
SNV Proportion (log-scale)
-15 -10 -5 0
0 Mb
18 Mb
35 Mb
52 Mb
69 Mb
86 Mb
103 Mb
120 Mb
137 Mb
154 Mb
171 Mb
Genomic Position

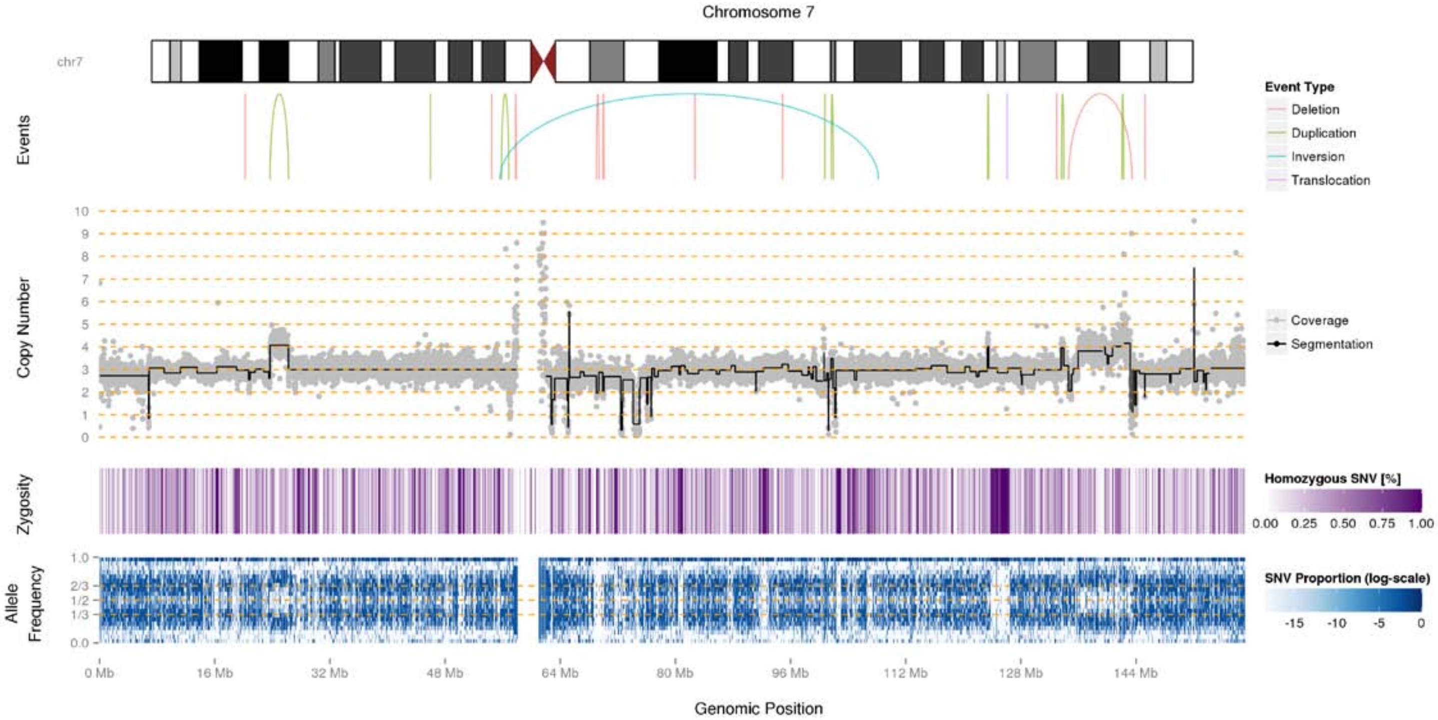
Chromosome 7
chr7
Events
Event Type
Deletion
Duplication
Inversion
Translocation
Copy Number
Coverage
Segmentation
Zygosity
Homozygous SNV [%]
0.00 0.25 0.50 0.75 1.00
Allele Frequency
SNV Proportion (log-scale)
-15 -10 -5 0
0 Mb 16 Mb 32 Mb 48 Mb 64 Mb 80 Mb 96 Mb 112 Mb 128 Mb 144 Mb
Genomic Position

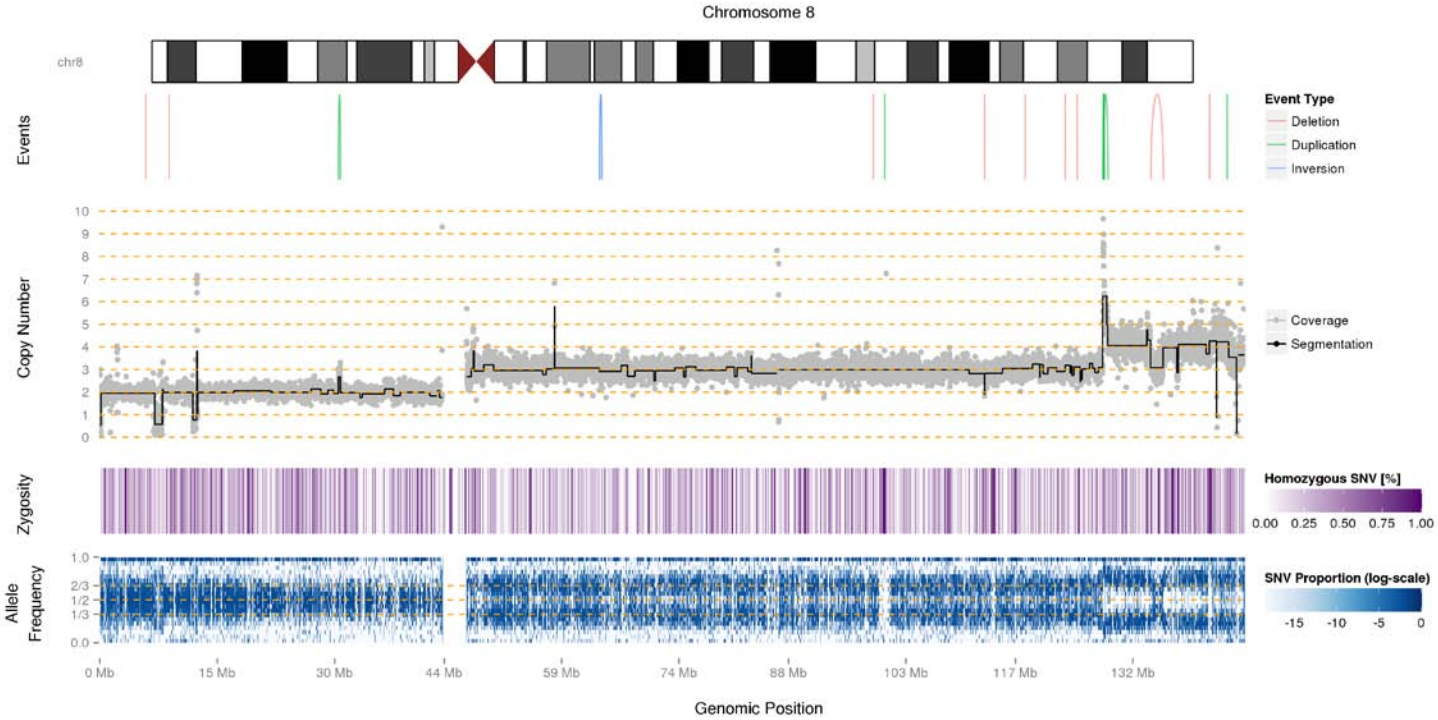
Chromosome 8
chr8
Events
Event Type
Deletion
Duplication
Inversion
Copy Number
Coverage
Segmentation
Zygosity
Homozygous SNV [%]
0.00 0.25 0.50 0.75 1.00
Allele Frequency
SNV Proportion (log-scale)
-15 -10 -5 0
0 Mb 15 Mb 30 Mb 44 Mb 59 Mb 74 Mb 88 Mb 103 Mb 117 Mb 132 Mb
Genomic Position

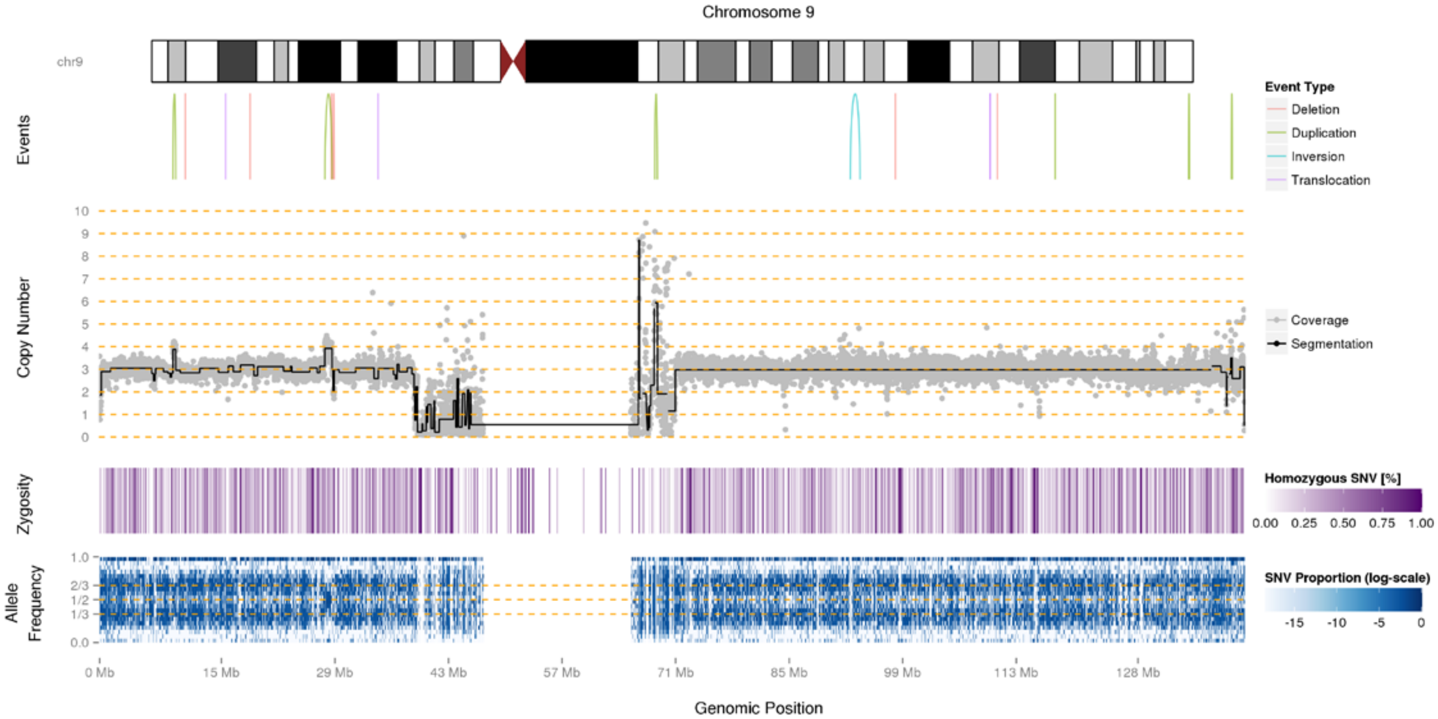
Chromosome 9
chr9
Events
Event Type
Deletion
Duplication
Inversion
Translocation
Copy Number
Coverage
Segmentation
Zygosity
Homozygous SNV [%]
0.00 0.25 0.50 0.75 1.00
Allele Frequency
SNV Proportion (log-scale)
-15 -10 -5 0
0 Mb 15 Mb 29 Mb 43 Mb 57 Mb 71 Mb 85 Mb 99 Mb 113 Mb 128 Mb
Genomic Position

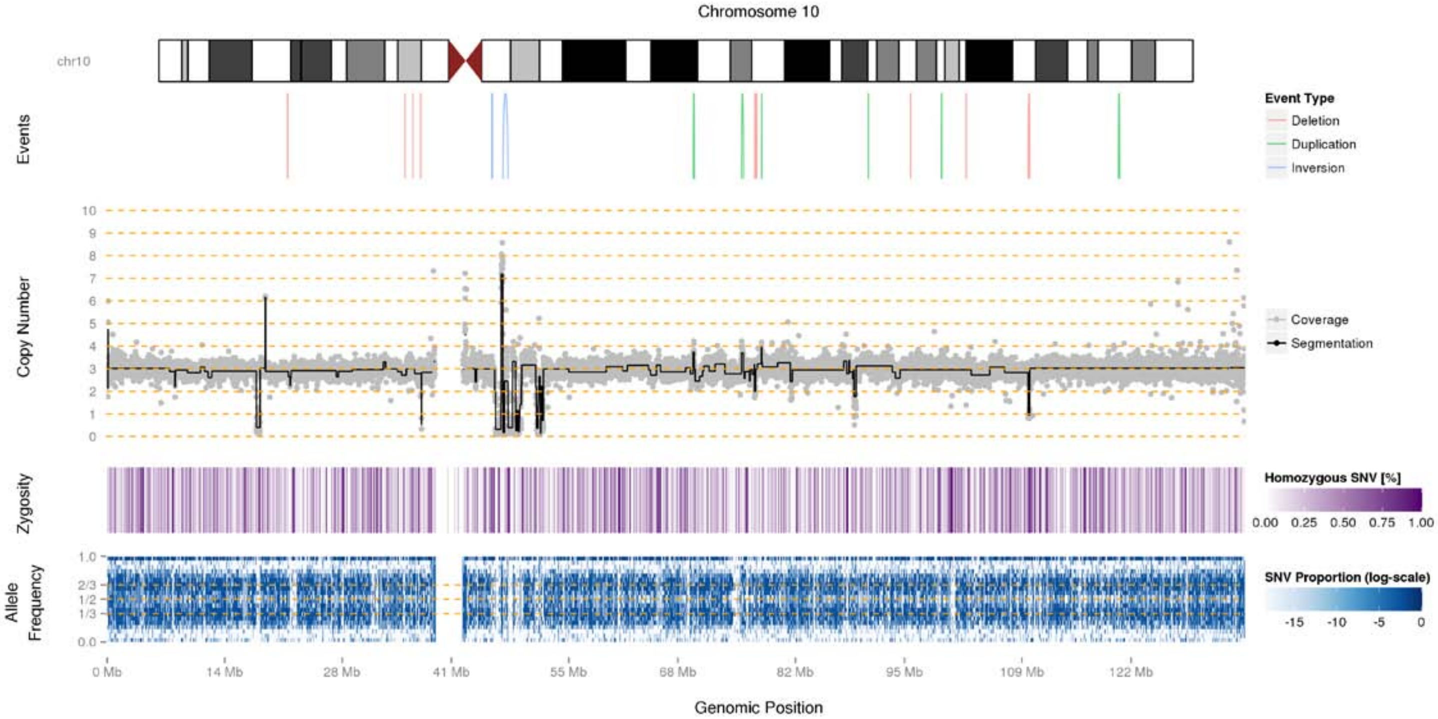
Chromosome 10
chr10
Events
Event Type
Deletion
Duplication
Inversion
Copy Number
Coverage
Segmentation
Zygosity
Homozygous SNV [%]
0.00 0.25 0.50 0.75 1.00
Allele Frequency
SNV Proportion (log-scale)
-15 -10 -5 0
0 Mb 14 Mb 28 Mb 41 Mb 55 Mb 68 Mb 82 Mb 95 Mb 109 Mb 122 Mb
Genomic Position

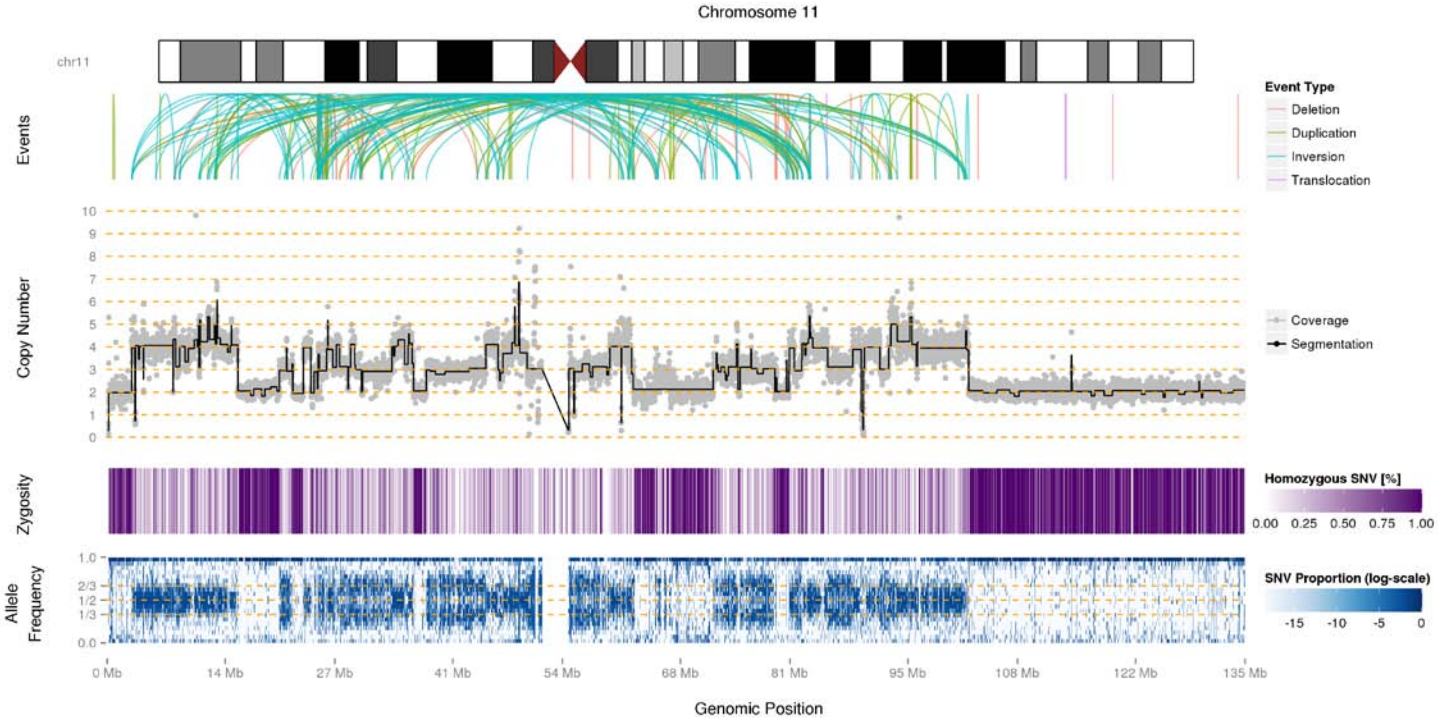

Chromosome 11
chr11
Events
Event Type
Deletion
Duplication
Inversion
Translocation
Copy Number
Coverage
Segmentation
Zygosity
Homozygous SNV [%]
0.00 0.25 0.50 0.75 1.00
Allele Frequency
SNV Proportion (log-scale)
-15 -10 -5 0
0 Mb 14 Mb 27 Mb 41 Mb 54 Mb 68 Mb 81 Mb 95 Mb 108 Mb 122 Mb 135 Mb
Genomic Position

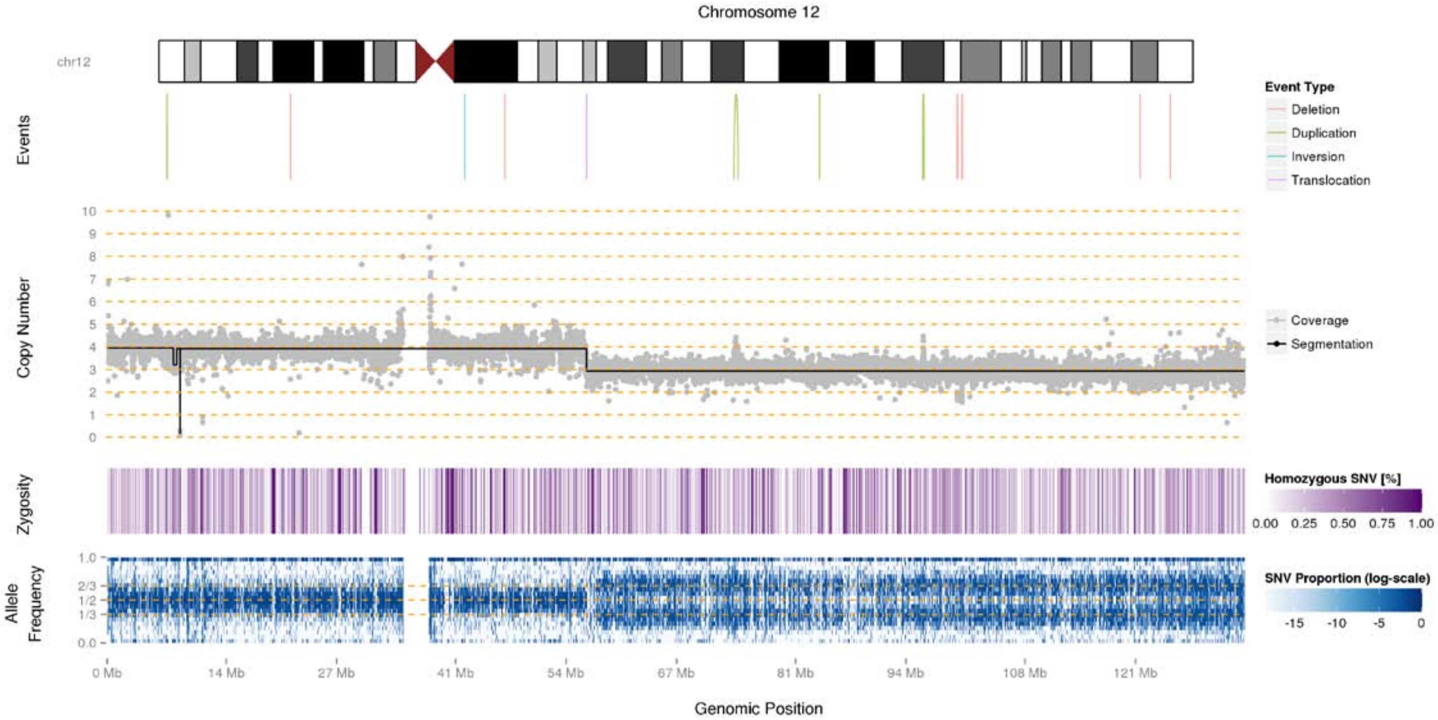

Chromosome 12
chr12
Events
Event Type
Deletion
Duplication
Inversion
Translocation
Copy Number
Coverage
Segmentation
Zygosity
Homozygous SNV [%]
0.00 0.25 0.50 0.75 1.00
Allele Frequency
SNV Proportion (log-scale)
-15 -10 -5 0
0 Mb 14 Mb 27 Mb 41 Mb 54 Mb 67 Mb 81 Mb 94 Mb 108 Mb 121 Mb
Genomic Position

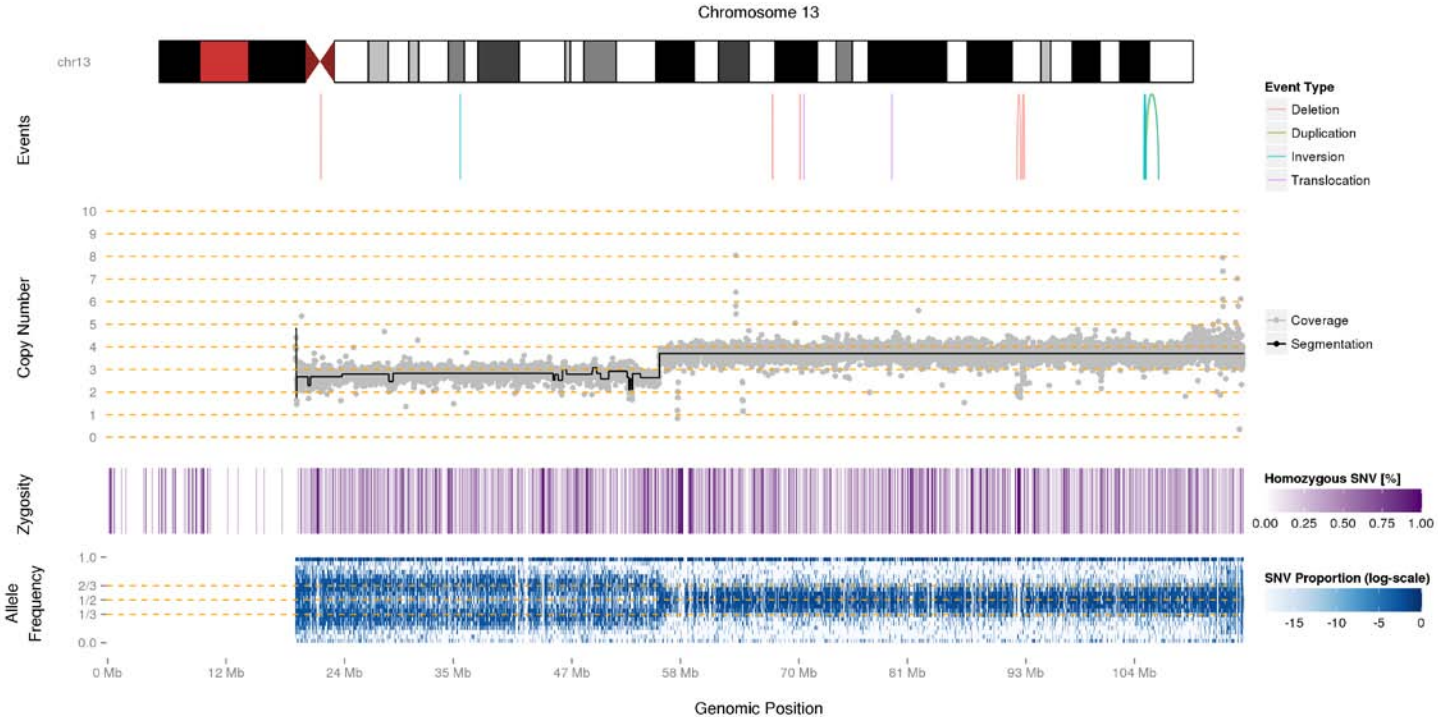
Chromosome 13
chr13
Events
Event Type
Deletion
Duplication
Inversion
Translocation
Copy Number
Coverage
Segmentation
Zygosity
Homozygous SNV [%]
0.00 0.25 0.50 0.75 1.00
Allele Frequency
SNV Proportion (log-scale)
-15 -10 -5 0
0 Mb 12 Mb 24 Mb 35 Mb 47 Mb 58 Mb 70 Mb 81 Mb 93 Mb 104 Mb
Genomic Position

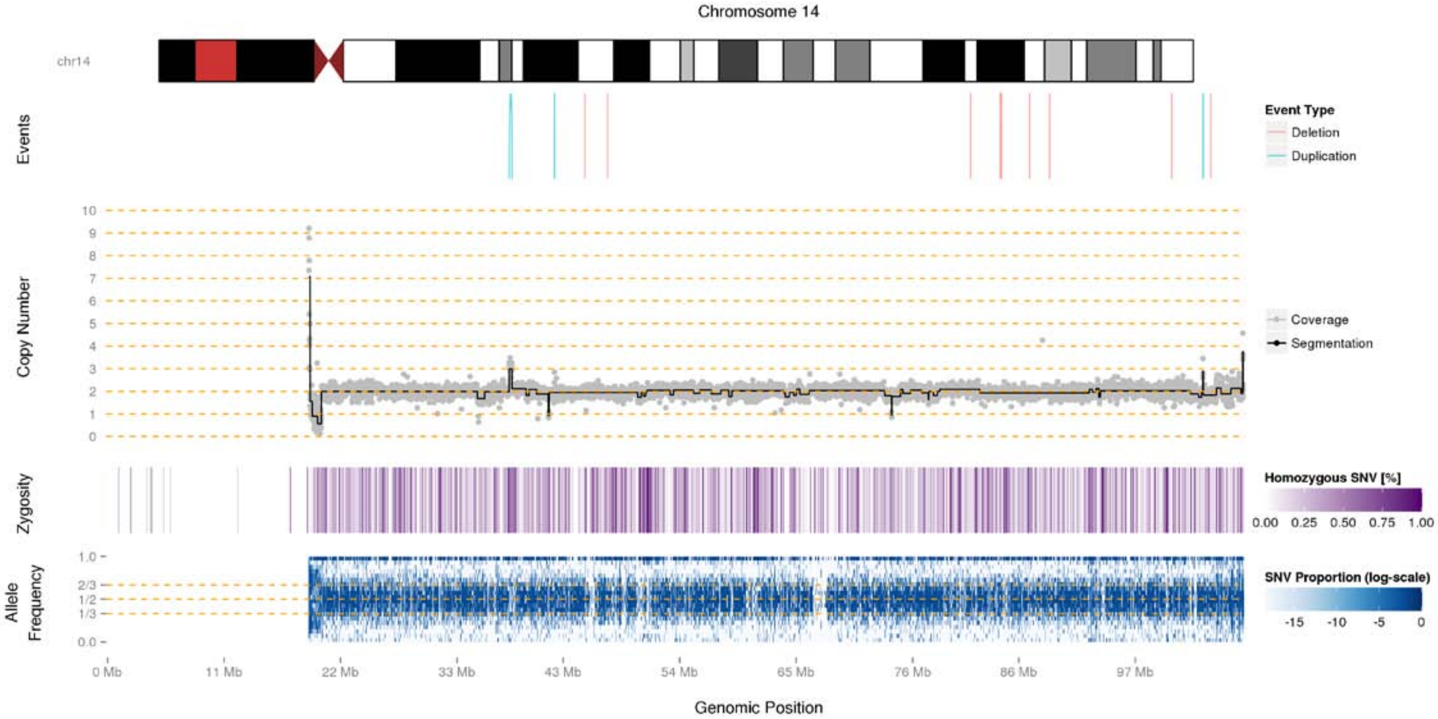
Chromosome 14
chr14
Events
Event Type
Deletion
Duplication
Copy Number
Coverage
Segmentation
Zygosity
Homozygous SNV [%]
0.00 0.25 0.50 0.75 1.00
Allele Frequency
SNV Proportion (log-scale)
-15 -10 -5 0
0 Mb 11 Mb 22 Mb 33 Mb 43 Mb 54 Mb 65 Mb 76 Mb 86 Mb 97 Mb
Genomic Position

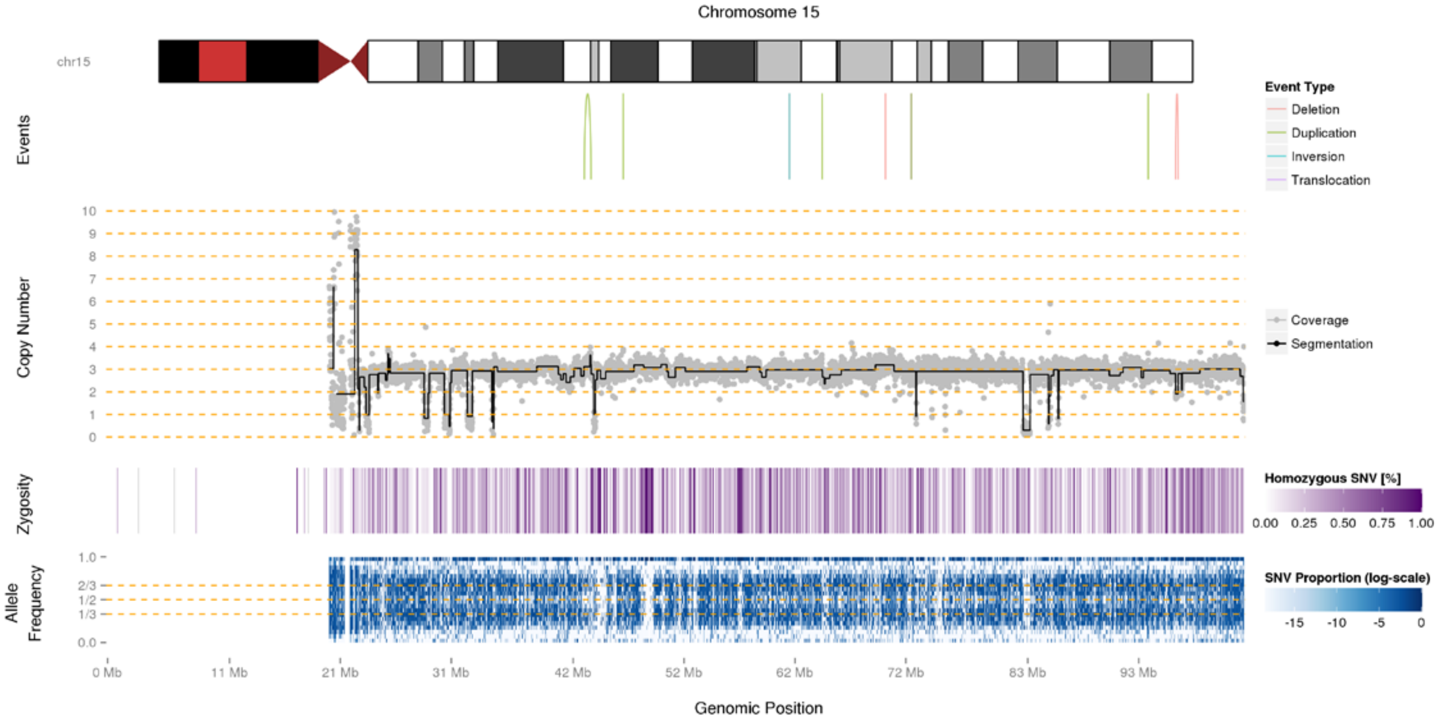
Chromosome 15
chr15
Events
Event Type
Deletion
Duplication
Inversion
Translocation
Copy Number
Coverage
Segmentation
Zygosity
Homozygous SNV [%]
0.00 0.25 0.50 0.75 1.00
Allele Frequency
SNV Proportion (log-scale)
-15 -10 -5 0
0 Mb 11 Mb 21 Mb 31 Mb 42 Mb 52 Mb 62 Mb 72 Mb 83 Mb 93 Mb
Genomic Position

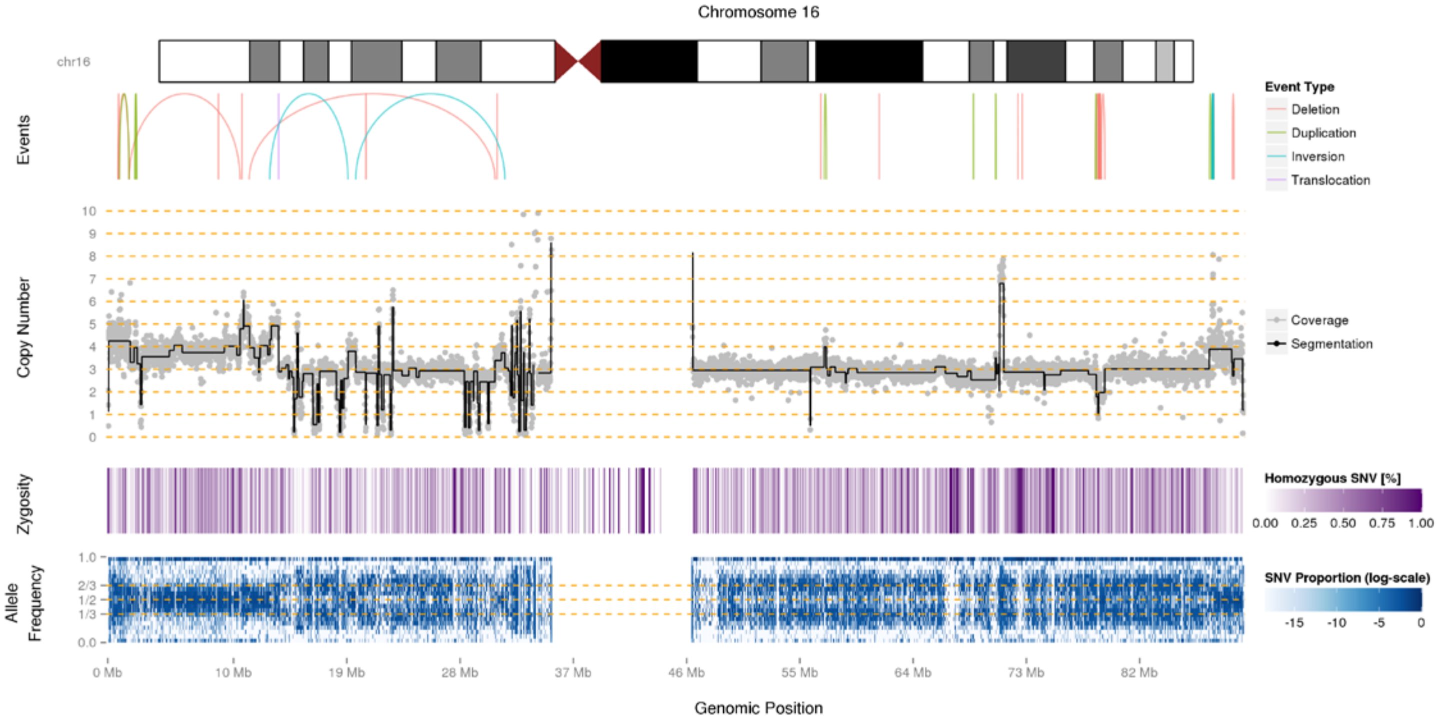
Chromosome 16
chr16
Events
Event Type
Deletion
Duplication
Inversion
Translocation
Copy Number
Coverage
Segmentation
Zygosity
Homozygous SNV [%]
0.00 0.25 0.50 0.75 1.00
Allele Frequency
SNV Proportion (log-scale)
-15 -10 -5 0
0 Mb 10 Mb 19 Mb 28 Mb 37 Mb 46 Mb 55 Mb 64 Mb 73 Mb 82 Mb
Genomic Position

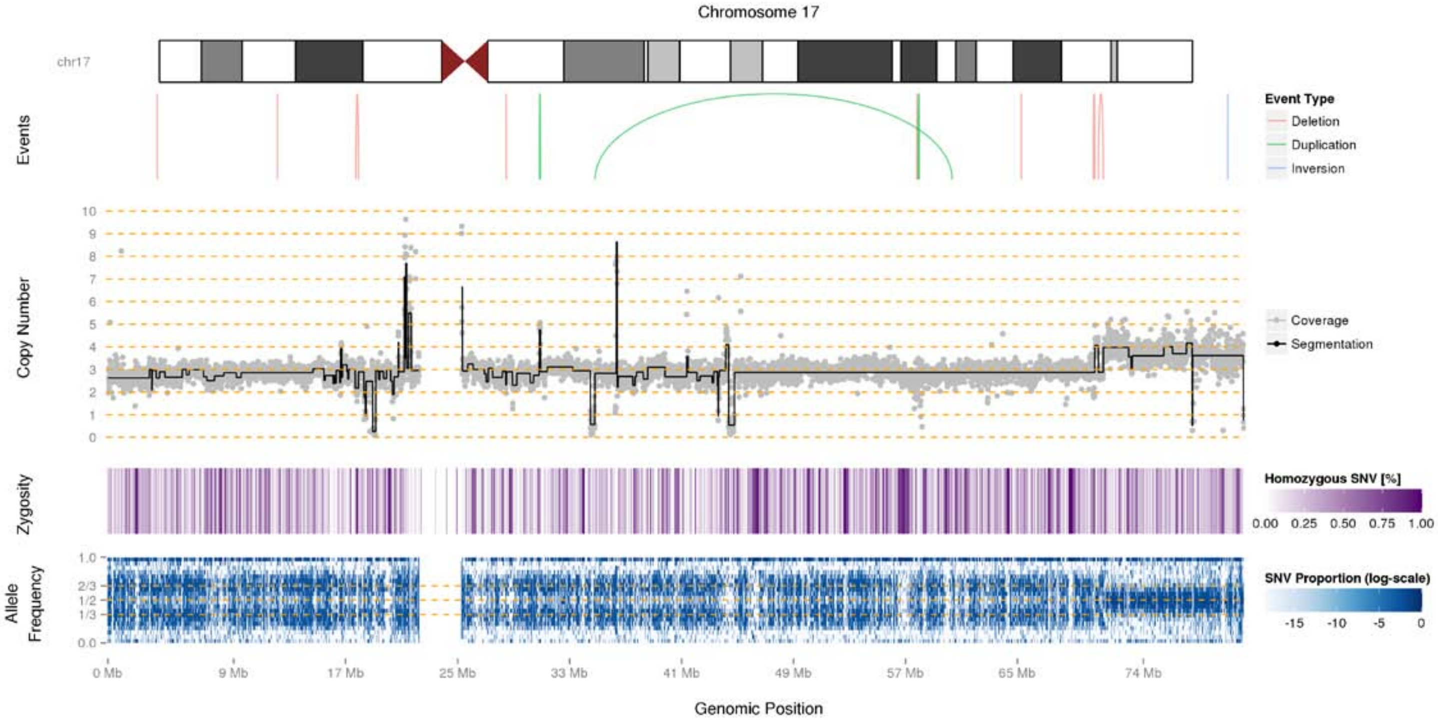
Chromosome 17
chr17
Events
Event Type
Deletion
Duplication
Inversion
Copy Number
Coverage
Segmentation
Zygosity
Homozygous SNV [%]
0.00 0.25 0.50 0.75 1.00
Allele Frequency
SNV Proportion (log-scale)
-15 -10 -5 0
0 Mb 9 Mb 17 Mb 25 Mb 33 Mb 41 Mb 49 Mb 57 Mb 65 Mb 74 Mb
Genomic Position

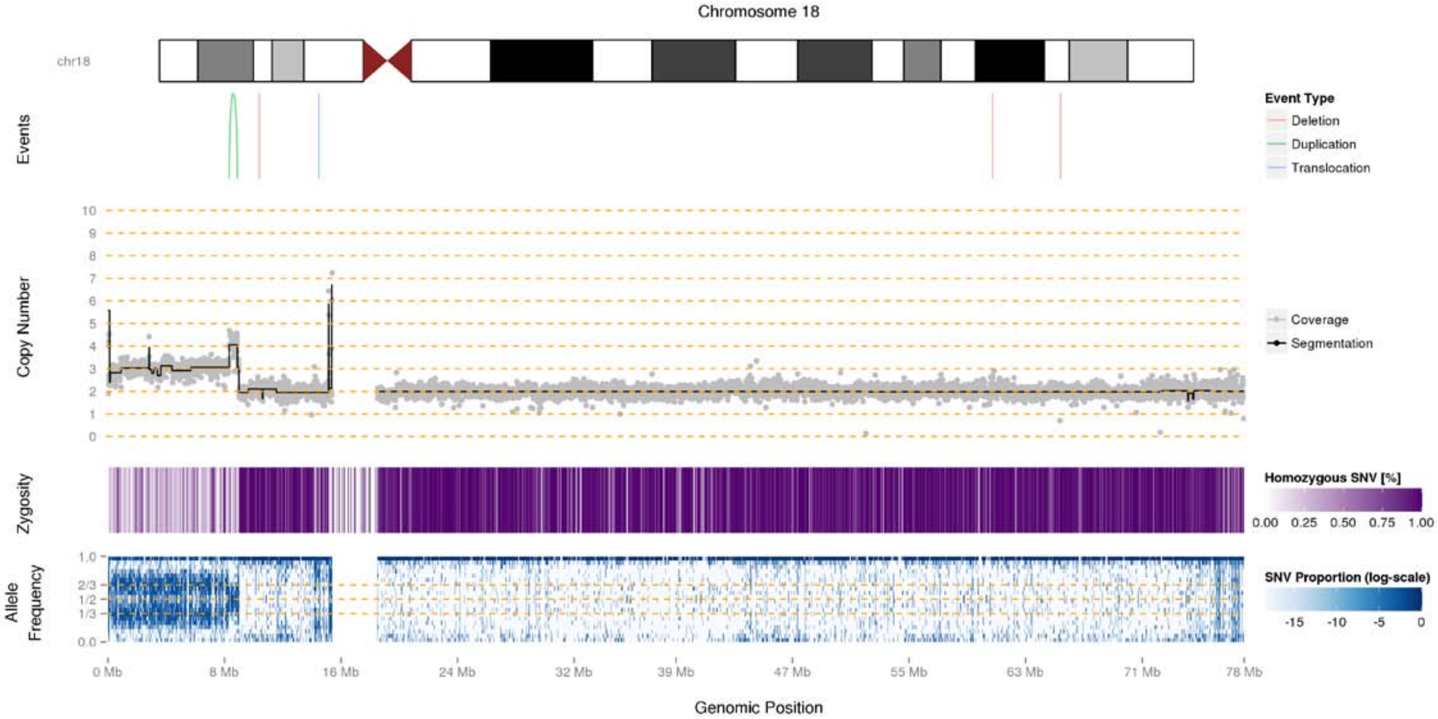
Chromosome 18
chr18
Events
Event Type
Deletion
Duplication
Translocation
Copy Number
Coverage
Segmentation
Zygosity
Homozygous SNV [%]
0.00 0.25 0.50 0.75 1.00
Allele Frequency
SNV Proportion (log-scale)
-15 -10 -5 0
0 Mb 8 Mb 16 Mb 24 Mb 32 Mb 39 Mb 47 Mb 55 Mb 63 Mb 71 Mb 78 Mb
Genomic Position

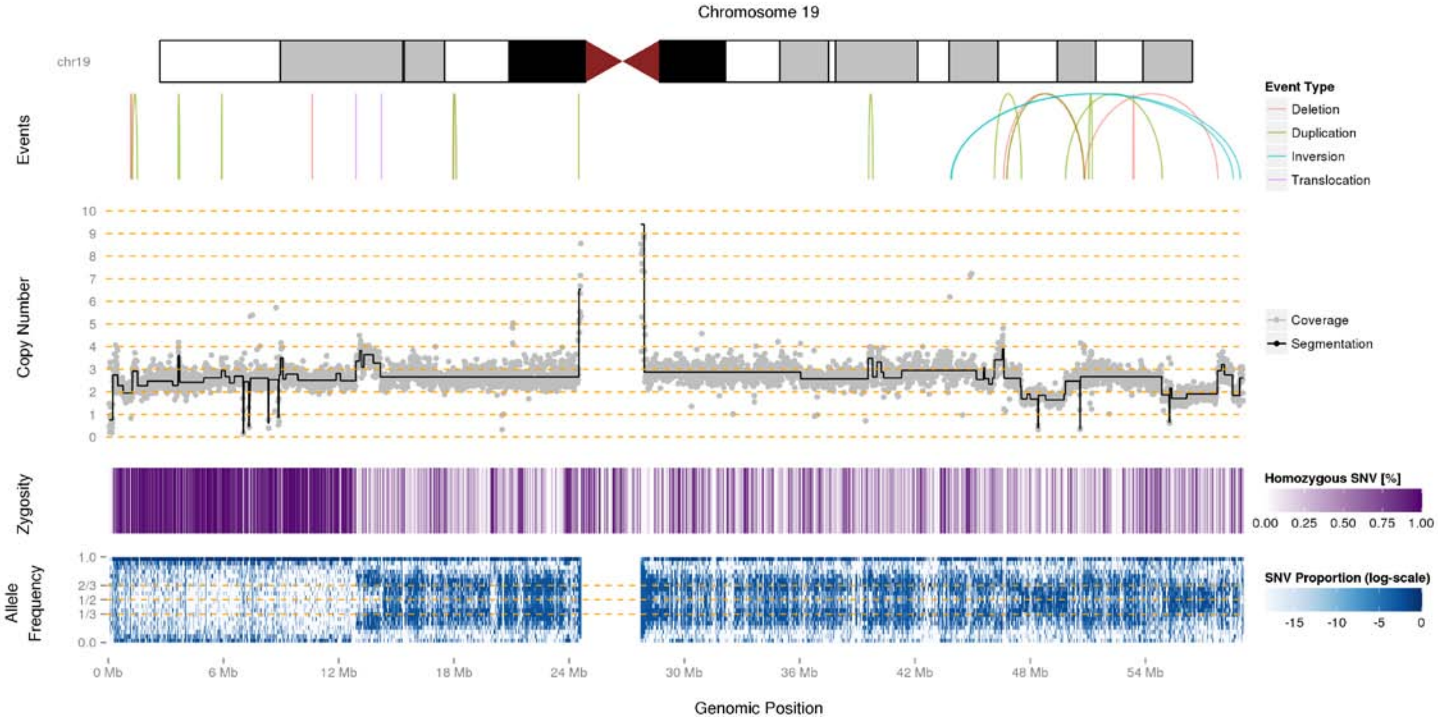

Chromosome 19
chr19
Events
Copy Number
Zygosity
Allele Frequency
Event Type
Deletion
Duplication
Inversion
Translocation
Coverage
Segmentation
Homozygous SNV [%]
0.00 0.25 0.50 0.75 1.00
SNV Proportion (log-scale)
-15 -10 -5 0
0 Mb 6 Mb 12 Mb 18 Mb 24 Mb 30 Mb 36 Mb 42 Mb 48 Mb 54 Mb
Genomic Position

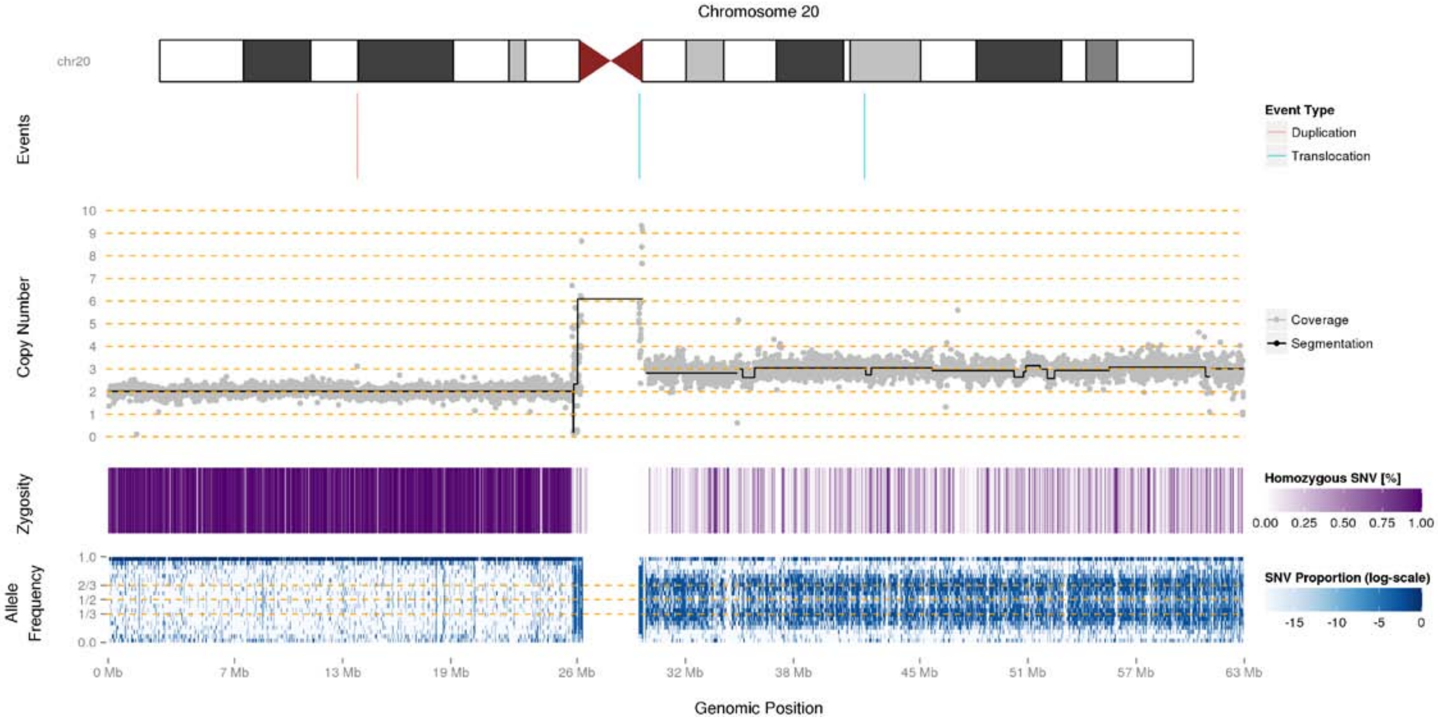

Chromosome 20
chr20
Events
Copy Number
Zygosity
Allele Frequency
Event Type
Duplication
Translocation
Coverage
Segmentation
Homozygous SNV [%]
0.00 0.25 0.50 0.75 1.00
SNV Proportion (log-scale)
-15 -10 -5 0
0 Mb 7 Mb 13 Mb 19 Mb 26 Mb 32 Mb 38 Mb 45 Mb 51 Mb 57 Mb 63 Mb
Genomic Position

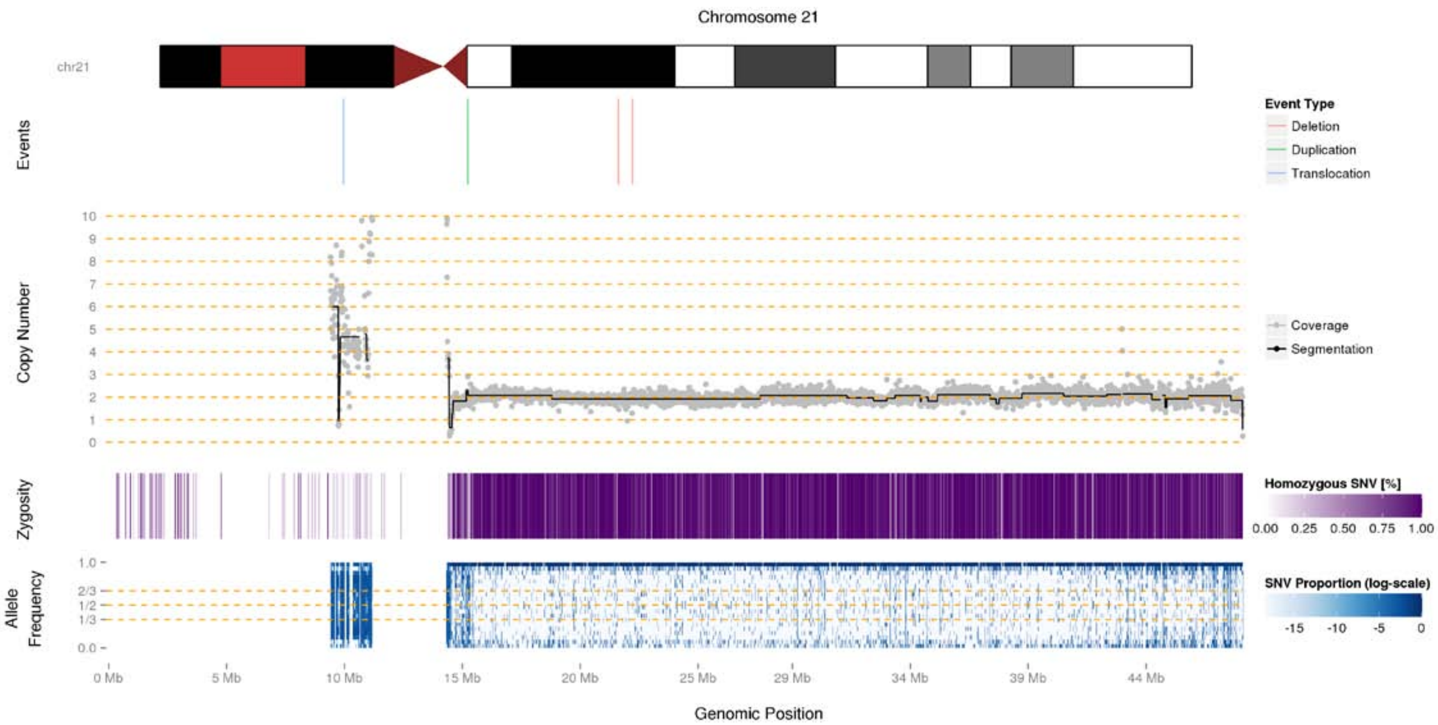
Chromosome 21
chr21
Events
Event Type
Deletion
Duplication
Translocation
Copy Number
Coverage
Segmentation
Zygosity
Homozygous SNV [%]
0.00 0.25 0.50 0.75 1.00
Allele Frequency
SNV Proportion (log-scale)
-15 -10 -5 0
0 Mb
5 Mb
10 Mb
15 Mb
20 Mb
25 Mb
29 Mb
34 Mb
39 Mb
44 Mb
Genomic Position

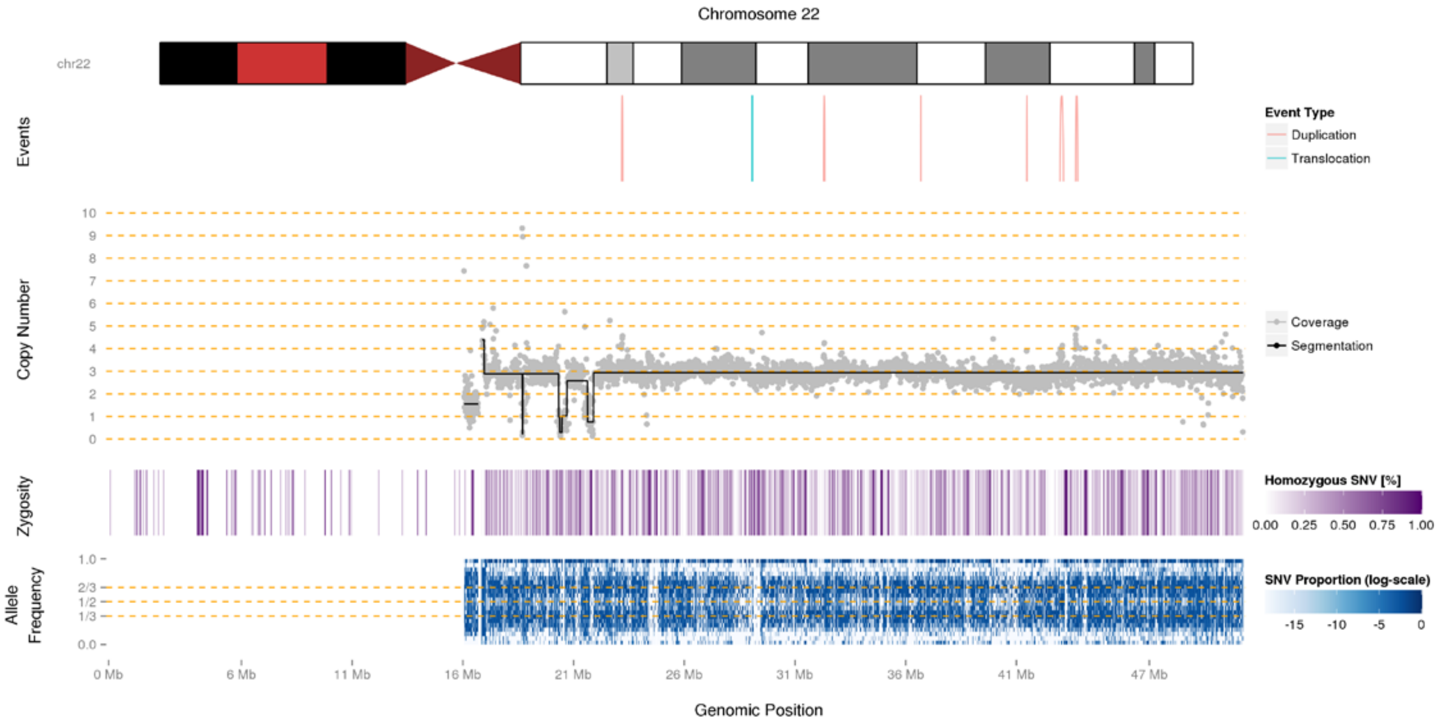
Chromosome 22
chr22
Events
Event Type
Duplication
Translocation
Copy Number
Coverage
Segmentation
Zygosity
Homozygous SNV [%]
0.00 0.25 0.50 0.75 1.00
Allele Frequency
SNV Proportion (log-scale)
-15 -10 -5 0
0 Mb
6 Mb
11 Mb
16 Mb
21 Mb
26 Mb
31 Mb
36 Mb
41 Mb
47 Mb
Genomic Position

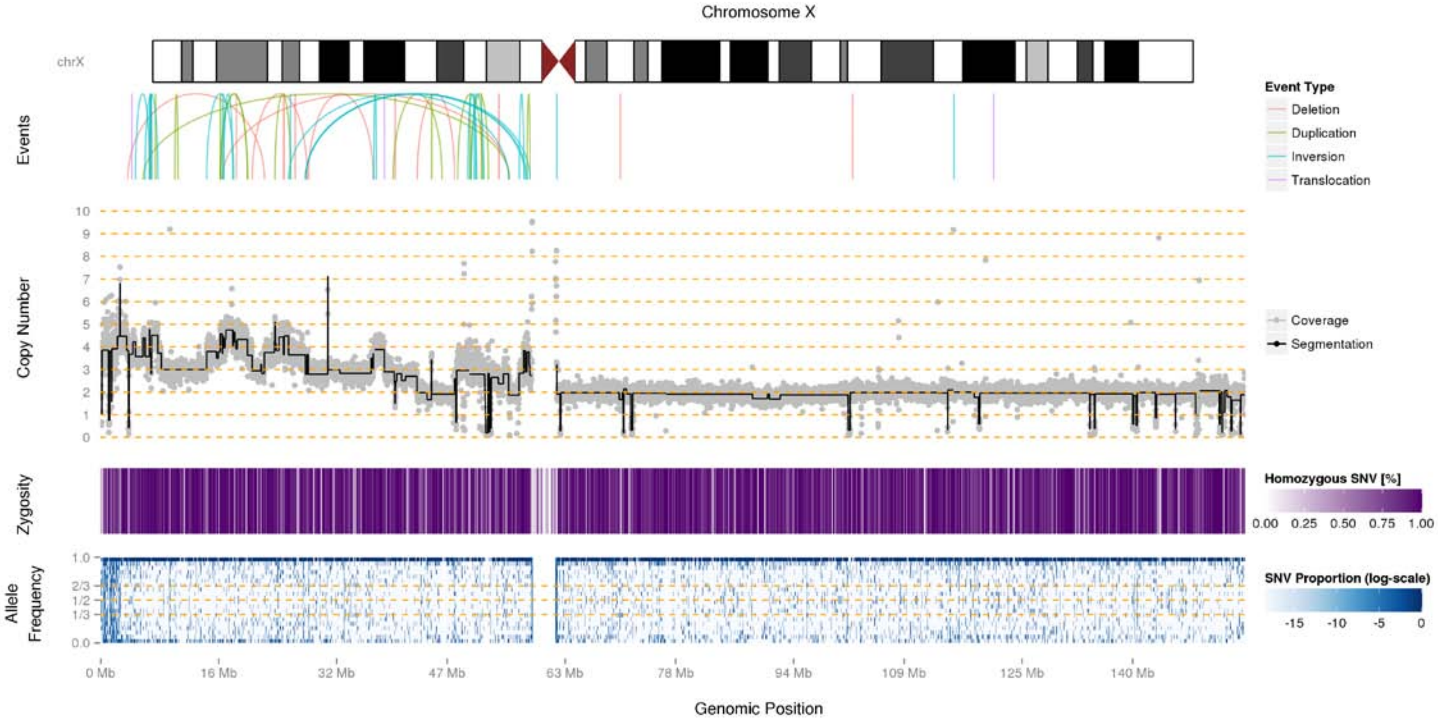

Figure S2 Structural variants, copy number and loss of heterozygosity for chromosomes 1 to 22 and X.

Arcs in the top panels labeled 'Events' represent the predicted connections between fragments derived from SV calls based on read pair orientation and spacing. Different read pair signatures indicate the following event types: deletions, tandem duplications, inversions, and interchromosomal translocations. The center panel (Copy Number) represents the copy number estimates in 10 kb bins (grey) overlaid with their segmentation (black). The associated CN is shown on the y-axis. The zygosity track shows the proportion of homozygous SNV calls in 10 kb bins, darker purple regions contain more homozygous calls (up to 100%) and indicate potential LoH. The bottom panel shows the allele frequency (AFS) distribution as a heatmap in 10 kb bins on the chromosome axis and 5% bins on the allele frequency axis; darker blue indicates more SNVs with the given AF in the corresponding 10 kb region. The color scale is according to the log of proportion of SNVs falling into the AFS bin (e.g. 10-15%, i.e. the row) in the 10 kb region (i.e. the column).
