# Supplementary material for: The Genomic and Transcriptomic Landscape of a HeLa Cell Line
Source: G3 (Bethesda). 2013 Mar 11;3(8):1213–24. doi: 10.1534/g3.113.005777 (PMC3737162; doi:10.1534/g3.113.005777)
Supplement: Supporting Information [file supp_g3.113.005777_FigureS3.pdf]

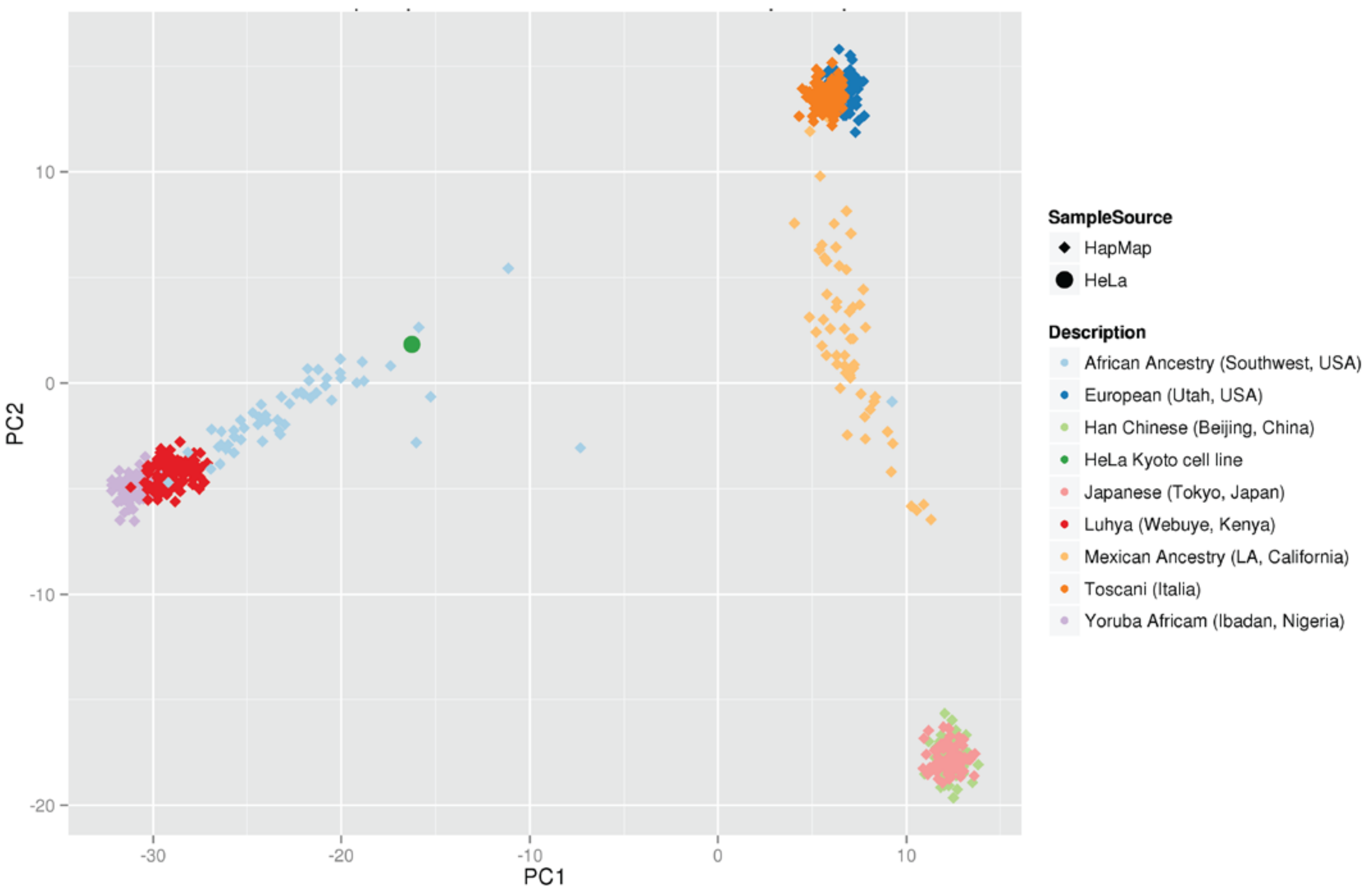

Figure S3 Principal component analysis of SNVs in HeLa Kyoto and 640 HapMap individuals from 8 different populations.
The populations are:
CEU - Northern and Western European ancestry from Utah, USA;
CHB - Han Chinese from Beijing, China;
JPT - Japanese from Tokyo, Japan;
YRI - Yoruba from Ibadan, Nigeria;
ASW - African ancestry from Southwest USA;
LWK - Luhya from Webuye, Kenya;
MXL - Mexican ancestry from Los Angeles, USA;
TSI - Toscani from Italy.
