# Supplementary material for: The Genomic and Transcriptomic Landscape of a HeLa Cell Line
Source: G3 (Bethesda). 2013 Mar 11;3(8):1213–24. doi: 10.1534/g3.113.005777 (PMC3737162; doi:10.1534/g3.113.005777)
Supplement: Supporting Information [file supp_g3.113.005777_FigureS4.pdf]

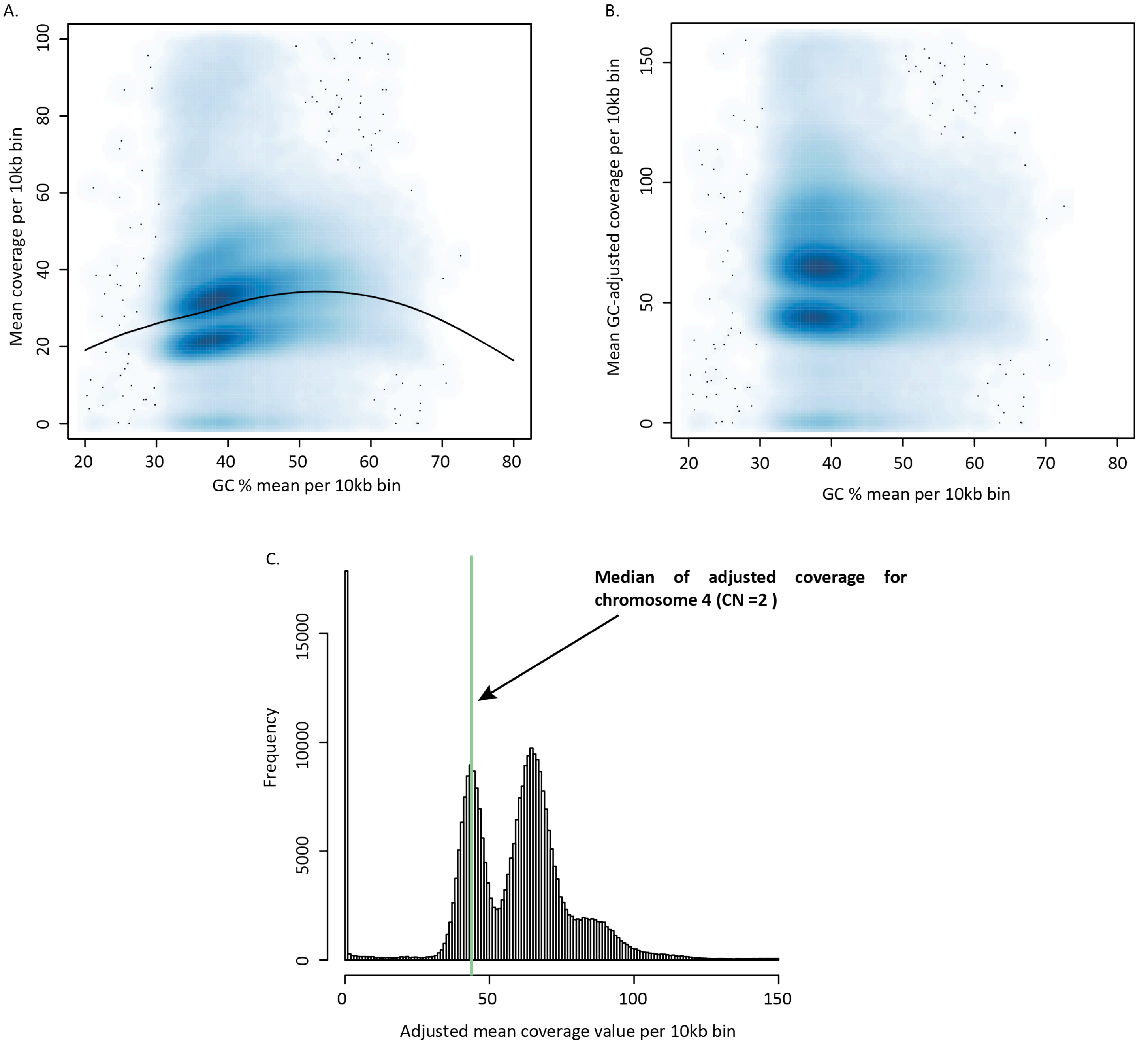

Figure S4 Effect of GC adjustment on sequencing coverage.

(A) Mean coverage value per 10 kb bin (y-axis) as a function of the mean GC % per 10 kb bin, before any adjustment was applied. The black line represents a robust local regression fitted on the data.

(B) Adjusted mean coverage value per 10 kb bin (y-axis) as a function of the mean GC % per 10 kb bin.

(C) Distribution of adjusted mean coverage value per 10 kb bins along the genome. The green line corresponds to the adjusted coverage median value for chromosome 4, which was used for normalization as representative of regions with CN 2.
