# Supplementary material for: The Genomic and Transcriptomic Landscape of a HeLa Cell Line
Source: G3 (Bethesda). 2013 Mar 11;3(8):1213–24. doi: 10.1534/g3.113.005777 (PMC3737162; doi:10.1534/g3.113.005777)
Supplement: Supporting Information [file supp_g3.113.005777_FigureS5.pdf]

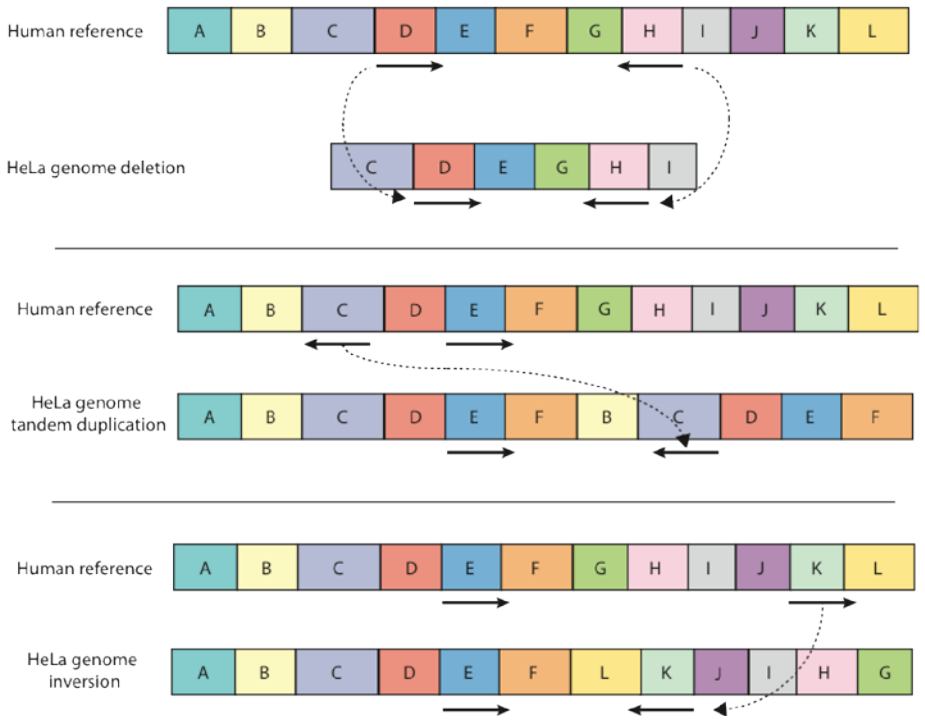

Figure S5 Primer design for detection of genomic rearrangements.
For deletions, the amplicons generated are smaller than expected size. The orientation of the primer targeting tandem duplication and inversion allow detection of an amplicon only if the event is present.
