# Supplementary material for: The Genomic and Transcriptomic Landscape of a HeLa Cell Line
Source: G3 (Bethesda). 2013 Mar 11;3(8):1213–24. doi: 10.1534/g3.113.005777 (PMC3737162; doi:10.1534/g3.113.005777)
Supplement: Supporting Information [file supp_g3.113.005777_FigureS6.pdf]

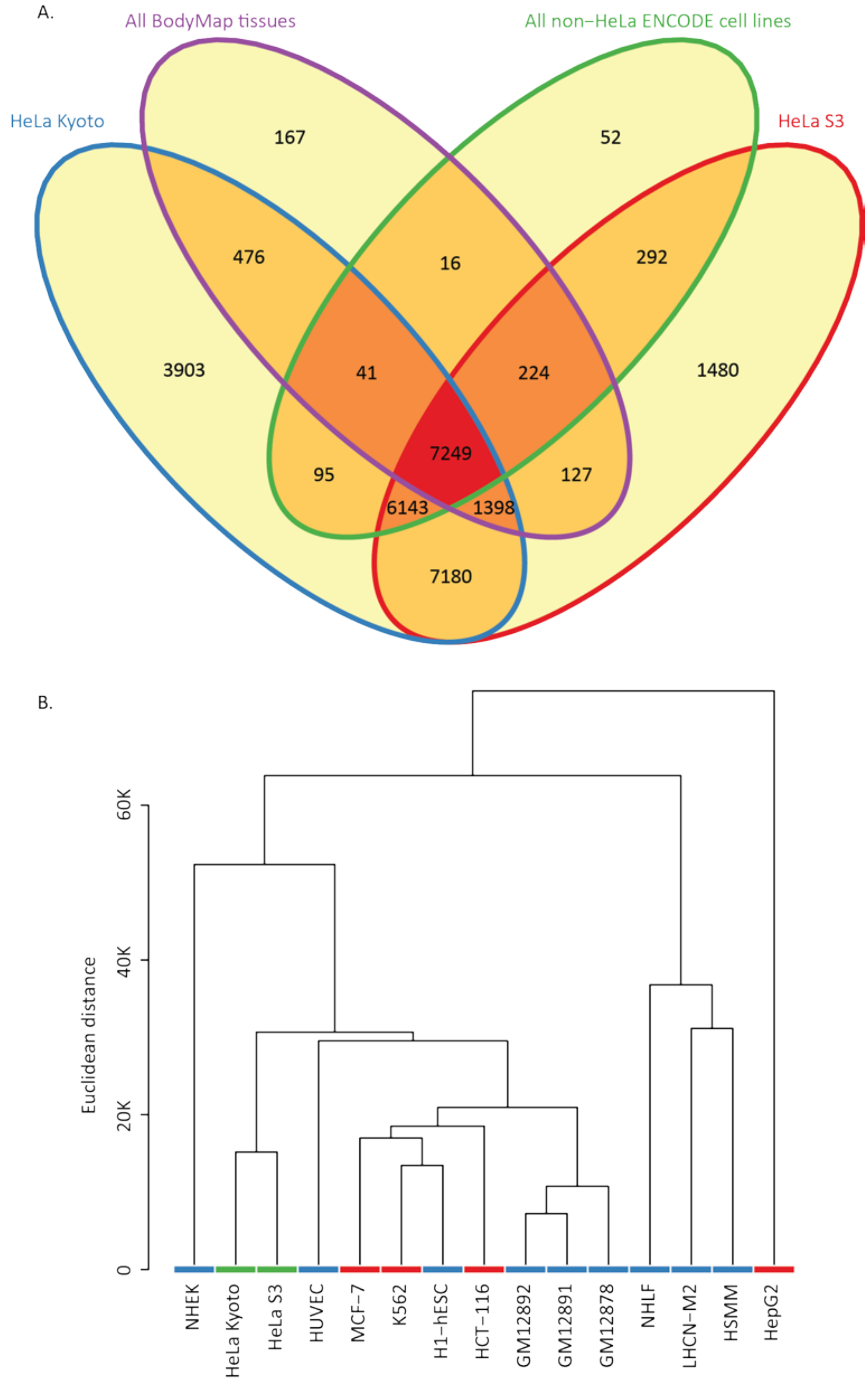

Figure S6 Comparison of HeLa transcriptome profile to Illumina Body Map tissues and ENCODE cell lines.
(A) Venn diagram of genes with nondetectable expression across all ENCODE cell lines (excluding HeLa-S3), all Body Map tissues, HeLa Kyoto and HeLa S3. The numbers indicate genes (listed in the human reference annotation file from ENSEMBL, see Methods).
(B) Dendrogram comparing the transcription profiles of all ENCODE cell lines and the HeLa Kyoto cell line. The y-axis is the Euclidean distance between cell lines.
