# Supplementary material for: The Genomic and Transcriptomic Landscape of a HeLa Cell Line
Source: G3 (Bethesda). 2013 Mar 11;3(8):1213–24. doi: 10.1534/g3.113.005777 (PMC3737162; doi:10.1534/g3.113.005777)
Supplement: Supporting Information [file supp_g3.113.005777_TableS1.pdf]

Table S1 Potential viral insertions

Chr	Start	End	Strand	Read support	Viral species
1	10002	10118	+	12	Human herpesvirus 6B<br>Equid herpesvirus 2<br>Human herpesvirus 6A<br>Human herpesvirus 7<br>Gallid herpesvirus 2<br>Gallid herpesvirus 3<br>Meleagrid herpesvirus 1<br>Ovine herpesvirus 2<br>Cyprinid herpesvirus 3<br>Saimiriine herpesvirus 1
8	128189764	128190032	-	552	Human papillomavirus 18 and 32<br>(Alphapapillomavirus 7 and 1)
8	128192272	128192499	+	23	Human papillomavirus 18<br>(Alphapapillomavirus 7)
8	128193167	128193435	+	174	
8	128200419	128200690	+	163	
12	49659073	49659136	-	6	Human herpesvirus 5
12	95467	95567	+	9	Human herpesvirus 6B<br>Human herpesvirus 6A<br>Human herpesvirus 7<br>Gallid herpesvirus 2<br>Gallid herpesvirus 3<br>Meleagrid herpesvirus 1<br>Cyprinid herpesvirus 3
13	19648667	19648780	+	7	Taterapox virus
13	19649043	19649209	-	8	
X	155185203	155185362	-	8	Human herpesvirus 6B<br>Equid herpesvirus 2<br>Human herpesvirus 6A<br>Human herpesvirus 7<br>Gallid herpesvirus 2<br>Gallid herpesvirus 3<br>Meleagrid herpesvirus 1<br>Ovine herpesvirus 2
