# Supplementary material for: The Genomic and Transcriptomic Landscape of a HeLa Cell Line
Source: G3 (Bethesda). 2013 Mar 11;3(8):1213–24. doi: 10.1534/g3.113.005777 (PMC3737162; doi:10.1534/g3.113.005777)
Supplement: Publisher's Statement 26 March 2013 [file supp_3_8_1213_v2_index.html]

The Genomic and Transcriptomic Landscape of a HeLa Cell Line — Publisher's Statement 26 March 2013 

# The Genomic and Transcriptomic Landscape of a HeLa Cell Line

## Publisher's Statement 26 March 2013

**Files in this Data Supplement:**

- Publisher's Statement 26 March 2013
