# Supplementary material for: The Genomic and Transcriptomic Landscape of a HeLa Cell Line
Source: G3 (Bethesda). 2013 Mar 11;3(8):1213–24. doi: 10.1534/g3.113.005777 (PMC3737162; doi:10.1534/g3.113.005777)
Supplement: Publisher's Statement 26 March 2013 [file supp_g3.113.005777_G3Statement_005777_FINAL.pdf]

Genetics Society of America

9650 Rockville Pike, Bethesda, MD 20814-3998
Phone: (301) 634-7300 • Fax: (301) 634-7079
society@genetics-gsa.org
www.genetics-gsa.org

26 March 2013

After serious consideration and extensive discussion among The Editors, authors, and other stakeholders, at this time, *G3: Genes|Genomes|Genetics* has elected to allow the Early Online version of Landry *et al.* (2013), The Genomic and Transcriptomic Landscape of a HeLa Cell Line, *published Early Online March 11, 2013, doi:10.1534/g3.113.005777,* to remain online. We are actively working to arrive at a solution where the final published manuscript and associated data addresses the concerns raised while maintaining the standards of the GSA journals and our data policy[1].

The article's final online publication, originally intended for April 2013, has been rescheduled for the May issue, to allow the researchers and other parties a reasonable timeframe to arrive at a mutual agreement regarding the availability of data. Please contact us if you have additional questions.

Brenda Andrews, PhD
Editor in Chief, *G3: Genes|Genomes|Genetics*
Charles H. Best Chair of Medical Research
Professor and Chair, Banting & Best Department of Medical Research
Director, The Donnelly Centre for Cellular and Biomolecular Research
University of Toronto
Rm 230 160 College Street
Toronto, Ontario
M5S 3E1
phone: 416-978-8562
brenda.andrews@utoronto.ca

Tracey DePellegrin Connelly
Executive Editor, GSA Journals
Genetics Society of America
phone: 412-760-5391
tracey.depellegrin@thegsajournals.org
www.g3journal.org
www.genetics.org

G3 · Genes | Genomes | Genetics

[1] http://www.g3journal.org/site/misc/ifora.xhtml#DATA%20AND%20REAGENT%20POLICY
