# Supplementary material for: The Genomic and Transcriptomic Landscape of a HeLa Cell Line
Source: G3 (Bethesda). 2013 Mar 11;3(8):1213–24. doi: 10.1534/g3.113.005777 (PMC3737162; doi:10.1534/g3.113.005777)
Supplement: Landry_EMBL Statement_Data 19 March 2013 [file supp_3_8_1213_v3_index.html]

The Genomic and Transcriptomic Landscape of a HeLa Cell Line — Landry\_EMBL Statement\_Data 19 March 2013 

# The Genomic and Transcriptomic Landscape of a HeLa Cell Line

## Landry\_EMBL Statement\_Data 19 March 2013

**Files in this Data Supplement:**

- Landry\_EMBL Statement\_Data 19 March 2013
