# Supplementary material for: The Genomic and Transcriptomic Landscape of a HeLa Cell Line
Source: G3 (Bethesda). 2013 Mar 11;3(8):1213–24. doi: 10.1534/g3.113.005777 (PMC3737162; doi:10.1534/g3.113.005777)
Supplement: Landry_EMBL Statement_Data 19 March 2013 [file supp_g3.113.005777_Landry_2013_EMBLStatement_Data.pdf]

19 March 2013

EMBL

EMBL Heidelberg • Meyerhofstr. 1 • 69117 Heidelberg • Germany

Our genomic data from the HeLa cell line [published with Landry et al. 2013; doi:10.1534g3.113.005777] are currently not publicly accessible by agreement between the authors and the journal *G3: Genes|Genomes|Genetics*. After the article was published, relatives of Henrietta Lacks expressed concerns about the ways in which genetic data from HeLa cells may affect their privacy. We are currently withholding these data out of respect for the Lacks family, while discussions about access to genetic or sequencing-related data of HeLa cells are ongoing.
